# Effectiveness of non-pharmaceutical interventions in nine fields of activity to decrease SARS-CoV-2 transmission (Spain, September 2020–May 2021)

**DOI:** 10.3389/fpubh.2023.1061331

**Published:** 2023-04-12

**Authors:** Inés Barbeito, Daniel Precioso, María José Sierra, Susana Vegas-Azcárate, Sonia Fernández Balbuena, Begoña Vitoriano, David Goméz-Ullate, Ricardo Cao, Susana Monge, the Study Group for Non-Pharmaceutical Interventions in Spain

**Affiliations:** ^1^Research Centre for Communication and Information Technology (CITIC), University of A Coruña (UDC), Galicia, Spain; ^2^Department of Informatics Engineering, School of Engineering, University of Cádiz, Andalusia, Spain; ^3^Centre for the Coordination of Health Alerts and Emergencies, Ministry of Health, Madrid, Spain; ^4^CIBER Infectious Diseases, Madrid, Spain; ^5^Ministry of Economic Affairs and Digital Transformation, Madrid, Spain; ^6^Institute of Interdisciplinary Mathematics, Complutense University, Madrid, Spain; ^7^School of Science and Technology, IE University, Madrid, Spain; ^8^Department of Communicable Diseases, National Centre of Epidemiology, Institute of Health Carlos III, Madrid, Spain

**Keywords:** COVID-19, SARS-CoV-2, pandemic, non-pharmaceutical interventions (NPI), effectiveness, stringency index, logarithmic return, hierarchical models

## Abstract

**Background:**

We estimated the association between the level of restriction in nine different fields of activity and SARS-CoV-2 transmissibility in Spain, from 15 September 2020 to 9 May 2021.

**Methods:**

A stringency index (0–1) was created for each Spanish province (*n* = 50) daily. A hierarchical multiplicative model was fitted. The median of coefficients across provinces (95% bootstrap confidence intervals) quantified the effect of increasing one standard deviation in the stringency index over the logarithmic return of the weekly percentage variation of the 7-days SARS-CoV-2 cumulative incidence, lagged 12 days.

**Results:**

Overall, increasing restrictions reduced SARS-CoV-2 transmission by 22% (RR = 0.78; one-sided 95%CI: 0, 0.82) in 1 week, with highest effects for culture and leisure 14% (0.86; 0, 0.98), social distancing 13% (0.87; 0, 0.95), indoor restaurants 10% (0.90; 0, 0.95) and indoor sports 6% (0.94; 0, 0.98). In a reduced model with seven fields, culture and leisure no longer had a significant effect while ceremonies decreased transmission by 5% (0.95; 0, 0.96). Models *R*^2^ was around 70%.

**Conclusion:**

Increased restrictions decreased COVID-19 transmission. Limitations include remaining collinearity between fields, and somewhat artificial quantification of qualitative restrictions, so the exact attribution of the effect to specific areas must be done with caution.

## Introduction

The rapid expansion of the COVID-19 epidemic by March 2020, forced many countries to implement stringent restrictions and lockdowns, which managed to control transmission (1–3). Studies in this first wave assessed effectiveness of different non-pharmaceutical interventions (NPIs) (1, 3–7). However, the translation of evidence into recommendations was challenging. First, the high temporal correlation in the implementation of measures made causal attribution possibly spurious (8–10). Second, the marginal benefit of restrictions could be lower in the second and successive waves, where public awareness and use of facemasks was widespread. Third, very restrictive NPIs had high economic, social and mental health costs, resulted in increased social and gender inequalities (11, 12) and could not be sustained over time.

In the successive epidemic waves, most countries in Europe implemented NPIs with more granularity guided by epidemiological indicators, and targeting different fields of activity, shaped by economic, political and socio-cultural preferences (10, 13). The more time-spaced implementation of NPIs and the varying intensity has allowed to better disentangle the effects of NPIs (10, 13–15). Cultural factors may determine different number and intensity of social contacts in different venues across the different contexts, making it relevant to investigate the effect of NPI in varied settings.

Spain is a highly decentralized 47 million people country, divided into 17 Autonomous Communities (17 AC, with 50 Provinces), and 2 Autonomous Cities. In the first COVID-19 wave, a first state of alarm imposed a total lockdown on 14 March 2020 (16). From 4 May to 21 June 2020, restrictions were progressively relaxed (17), until the only measures in place were compulsory use of facemasks, general hygiene and ventilation measures and minimum interpersonal distance of 1.5 m at work, commerce and restaurants (18). By mid-August cases started rising and a second epidemic wave became widespread by mid-September. A national risk assessment framework with alert levels and tiered restrictions was agreed by all AC on 22 October (19). However, it was adopted as a voluntary guideline. On 25 October, the Government declared a second state of alarm (20), with a minimum curfew between 00:00 and 5:00, restrictions to movements between AC, and prohibition of groups over six non-household members. AC were free to adapt these measures, which provided a natural experiment of NPIs in 50 territories. A previous study in 7 Spanish provinces drew inconsistent results, with the limitation of seating capacity showing association with reduced transmission in outdoor, but not indoor settings (21). This could be explained by the reduced number of restrictions considered, and by false attribution of the effect of unmeasured restrictions to measured ones.

The objective of this work is to analyze the effectiveness of NPIs implemented in nine different fields of activity to decrease SARS-CoV-2 transmission in Spain from 15 September 2020 (beginning of the second epidemic wave) to 9 May 2021 (end of the second state of alarm).

## Methods

### Non-pharmaceutical interventions data and creation of a stringency index

We reviewed all 17 AC Official Bulletins, extracted and coded a pre-defined list of NPIs (Supplementary Appendix I), together with the days of start and end, at the province level, weighted by the proportion of population affected when the measure did not affect the entire province. Some measures were not included due to lack of variability in the period (closure of nightclubs, recommendations to work from home, compulsory use of facemasks, or measures in schools and kindergartens, which remained open) or because they were not ruled centrally (semi-present education at Universities).

Non-pharmaceutical interventions were grouped in nine fields of activity: Indoor sports (INSP, maximum capacity of venues, number of persons that could train together, public during events, or closure of installations), Outdoor sports (OUTSP, similar to INSP but outdoors), Culture and leisure venues (CULT, maximum capacity or closure of museums, monuments, cinemas, theaters, zoos, amusement parks), Ceremonies and religious celebrations (CERE, maximum capacity or cancelation of funerals, weddings and baptisms, and activities in religious temples), Commerce (COMM, maximum capacity, opening hours or closure of retail services, close-contact activities, malls and/or street markets), Indoor bars and restaurants (INRE, maximum capacity, persons per table, prohibition to use the bar, opening hours, or total closure of indoor spaces), Outdoor bars and restaurants (OUTRE, similar to INRE but outdoors), Social distance (DIST, number of on-household members and in gatherings in public and/or private spaces, need for authorization or cancellation of events, recommendations to stay at home or mandatory home-confinement) and Mobility (MOBI, curfew and perimeter exit/entry restrictions).

Intensity of each NPI was graded as low, medium or high, respectively assigning values of 0.2, 0.5, and 1, to make the scale more sensitive to more stringent measures. NPIs in the same field were combined using weights (Supplementary Appendices II, III), agreed by the expert panel of project collaborators. The result was one stringency index from 0 to 1 per field of activity, day and province. The codified measures and stringency indices are available at the project website (22).

### Case data and specification of the outcome variable

Confirmed COVID-19 cases (diagnosed by Polymerase-Chain Reaction or Rapid Antigen Test) by day and province were extracted from the national surveillance database (23). Compulsory notification and exhaustive surveillance was in place throughout the study period. We computed 7-day cumulative incidence (IA7) as the number of COVID-19 cases over the previous 7 days per 100,000 inhabitants, to smooth week seasonality.

The growth ratio (GR) is typically used to represent the relative variation in IA7 in a week timeframe. Considering *IA7_t_* the IA7 at day *t*, we define *GR_t_* = (*IA7_t_*-*IA7_t-7_*)/*IA7_t-7_* = *IA7_t_*/*IA7_t-7_*-1. GR is not expected to behave symmetrically, since its smallest possible value is −1, while it does not have an upper limit. Just to use a symmetric analog of GR, the logarithmic return (LR) was also considered, defined as *LR_t_* = log(*IA7_t_*/*IA7_t-7_*) = log(1 + *GR_t_*). For moderate values of GR (e.g., between −20% and 20%) GR and LR are almost identical.

**Figure 1:**
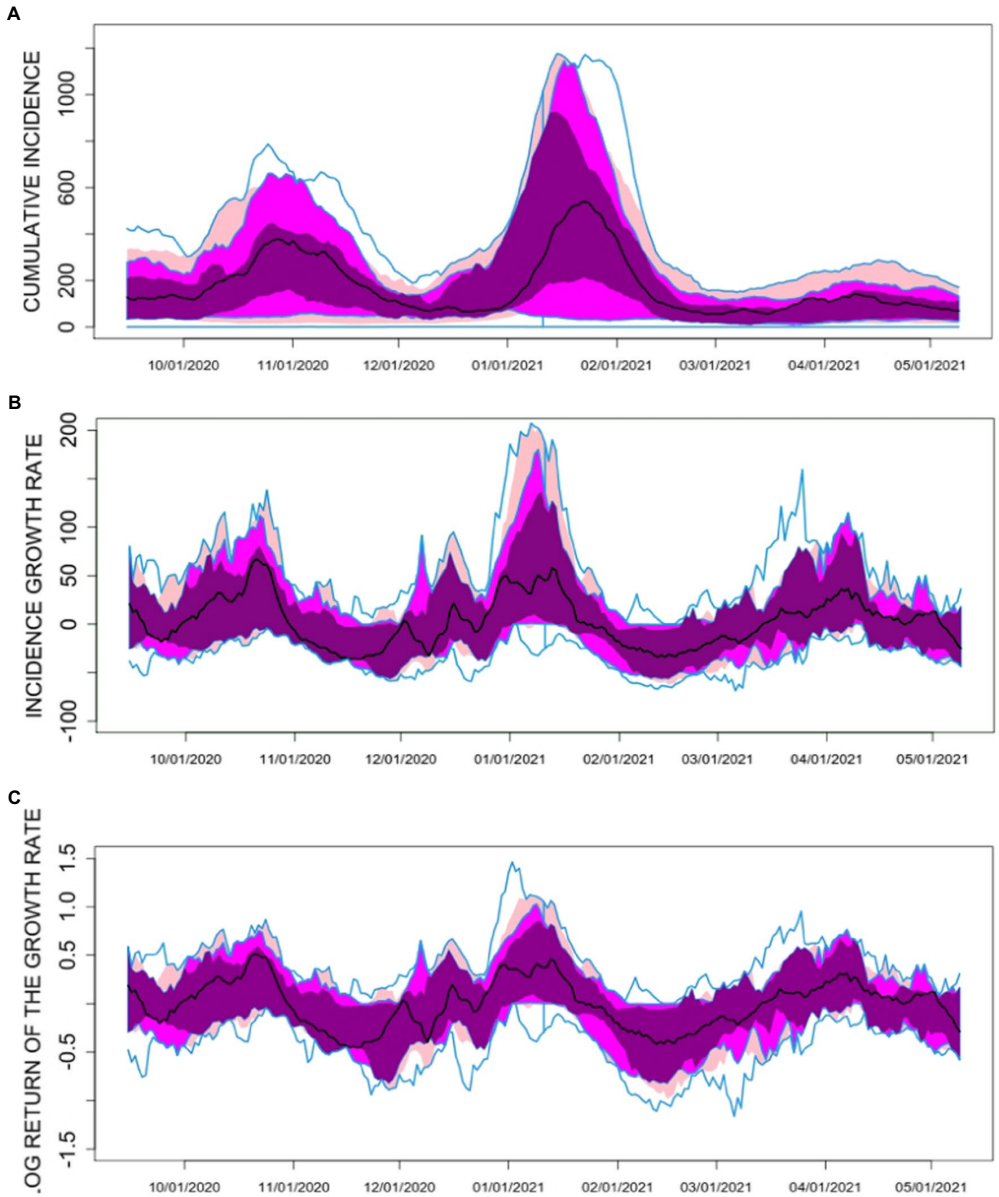
Time series boxplots for **(A)** the 7-day COVID-19 cumulative incidence (IA7), **(B)** the 7-day COVID-19 incidence growth rate (GR) and **(C)** the 7-day COVID-19 logarithmic return of the incidence growth rate (LR) in the 50 Spanish Provinces between 15 September 2021 and 9 May 2022. The black curve in the plot corresponds to the median function along the provinces. The dark magenta colored area in the plot represents the region that contains the central 25% of the curves along Spanish provinces. The magenta colored area includes the central 50% of the curves, while the pink area corresponds to the central 75% of the curves. The upper and lower blue solid lines account for the curve envelope, i.e., the most extreme values (minima and maxima) in the curves.

IA7, GR, and LR were assessed over time using time series (functional) boxplots (24) (Figure 1). These plots show the dispersion of the values over time across the 50 provinces, with the black line representing the time series median and the width variation of the colored bands the heterogeneity. IA7 showed a general overview of the pandemic evolution. However, the local maxima (peaks) of the time series median for LR and GR appeared a few days after the long weekends of 12 October and early December 2020 and after Christmas and New Year, capturing higher transmission on the days preceding case detection. LR showed less variability and more symmetry compared to GR, thus LR was considered more suitable as the response variable for the models. IA7, GR, and LR time series exhibited high autocorrelation.

This study does not involve human (expert or collaborators) or non-publicly available human data.

### Analytical methods

Since the effect of NPIs on SARS-CoV-2 transmission is not instantaneous, the series of NPI indices at day *t* (*NPI_t_*) and the lagged LR series *k* days ahead (*LR*_*t* + k_) were considered. Systematic fits of regression models for *LR*_*t* + k_ using *NPI_t_* were performed for *k* values between 0 and 30. Most models gave the largest *R*^2^ values for *k* = 12. Therefore, lagged LR values 12 days ahead (*LR*_*t* + 12_) were considered for the response variable (*Y*). The explanatory variables were the stringency indices for INSP (*X*_1_), OUTSP (*X*_2_), CULT (*X*_3_), CERE (*X*_4_), COMM (*X*_5_), INRE (*X*_6_), OUTRE (*X*_7_), DIST (*X*_8_) and MOBI (*X*_9_). NPI indices were normalized to obtain the effect of varying the index in 1 standard deviation.

Multiple linear regression models (MLR) and generalized additive models (GAM) for every province, mixed models (with random intercept, depending on the province, and fixed slope) and multiple linear and log-linear hierarchical models were used. For the GAM, a Gaussian family was considered, using thin plate regression penalized splines, with knots placed evenly throughout the covariate values. The most satisfactory model in terms of *R*^2^ was the hierarchical multiple linear model (adjusted *R*^2^ = 0.7049):

*Y* = 𝛃_0_ + 𝛃_1_·*X*_1_ + ··· + 𝛃_9_·*X*_9_ + 𝛆.

where *Y* = *LR*_*t* + 12_, 𝛃_0_,..., 𝛃_9_ are random coefficients that change from province to province, associated to the intercept and to the nine indices: *X*_1_,…,*X*_9_. Since *LR_t_* = log(*IA7_t_*/*IA7_t-7_*) the model can be rewritten in terms of the weekly IA7 ratio, thus obtaining an estimation of the Risk Ratio (*RR* = *IA7_t_*/*IA7_t-7_*):

*RR*= 𝛂_0_ · 𝛂_1_^*X*1^ · ··· · 𝛂_9_^*X*9^ · 𝛕.

where 𝛕 = exp.(𝛆) is a multiplicative error term and 𝛂_0_ = exp.(𝛃_0_), ..., 𝛂_9_ = exp.(𝛃_9_). This hierarchical multiplicative model (HMM) accounts for province variability (the coefficients 𝛂_i_ are random, since they depend on the province) and the NPI indices enter the model in a simple way (the *X*_i_ is just the exponent of 𝛂_i_). If 𝛂_i_ < 1, an increase in the stringency index, *X*_i_, would imply a reduction in the mean of the cumulative incidence weekly ratio. So 𝛂_i_ < 1 (or equivalently 𝛃_i_ < 0) implies that the NPIs summarized in *X*_i_ are associated to COVID-19 incidence reduction. Since the HMM depends on 500 parameters (10 coefficients for 50 provinces), significance of the estimated parameters have to be done with caution, avoiding multiple testing problems. Methods for controlling the FWER, family-wise error rate (25), and FDR, false discovery rate (26), have been used for *p*-value correction due to multiple testing. Model diagnostics have been performed based on the residuals, using statistical tests and exploratory plots to check normality, independence and homoscedasticity of the error terms in the models as well as outlier tests. Normality and homoscedasticity are accepted for most of the models, while independence is rejected.

To summarize the effect of every NPI index in the model, median values, along provinces, for every coefficient, 𝛂_i_, were estimated. The significance of the hypotheses Median (𝛂_i_) < 1, *i* = 1,...,9, were examined using the bootstrap method, which was also used to derive one-sided 95% confidence intervals (95% CI).

Finally, the average stringency index, *X_av_* = (*X*_1_ + ··· + *X*_9_)/9, was computed to explore the overall effect of NPIs. This is consistent with preliminary principal component analysis (not shown here) performed for the nine indices, in which a first principal component, accounting for about 60% of the total variability, exhibited balanced weights along the nine fields of activity, for most of the provinces. We described its correlation with *LR*_*t* + 12_ and performed scatterplots *X_av_*-*LR*_*t* + 12_ for each province and total Spain. *X_av_* was then introduced in a HMM similar to the one described above, to estimate its effect on *LR*_*t* + 12_. The degree to which the effect for *X_av_* approximates the sum of the individual effects for *X*_1_ + ··· + *X*_9_ depends on the correlation between them; therefore the comparison of the effects estimated for the average index and for the individual components is not straightforward.

## Results

### Description of the stringency index

**Figure 2:**
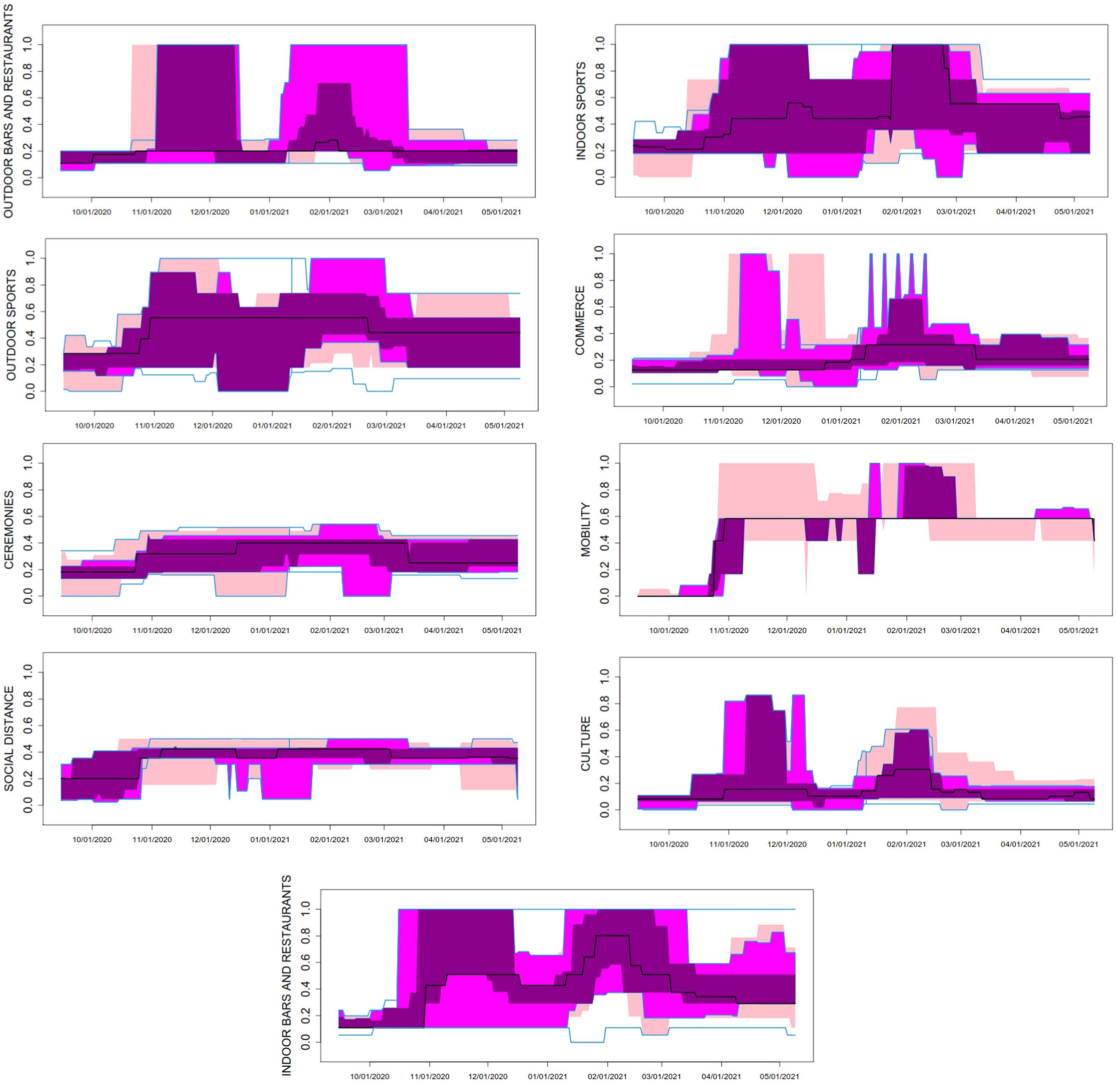
Time series boxplots for the daily stringency indices in nine fields of activity, in the 50 Spanish Provinces between 15 September 2021 and 9 May 2022. The black curve in the plot corresponds to the median function along the provinces. The dark magenta colored area in the plot represents the region that contains the central 25% of the curves along Spanish provinces. The magenta colored area includes the central 50% of the curves, while the pink area corresponds to the central 75% of the curves. The upper and lower blue solid lines account for the curve envelope, i.e., the most extreme values (minima and maxima) in the curves.

The daily stringency index for each field of activity and province can be found at the project website (22) and is summarized in the functional boxplots in Figure 2. Static boxplots for the distribution of the median and interquartile range of the nine stringency indexes can be found in Supplementary Appendix IV.

The fields with highest stringency index across the study period and the 50 provinces were MOBI (mean index = 0.4808), INSP (0.4784), INRE (0.4509), and OUTSP (0.4187); while mean stringency index was lower in DIST (0.3531), CERE (0.2997), OUTRE (0.2879), COMM (0.2346), and CULT (0.1680) (Table 1 and Supplementary Appendix IV). Most mean indices lay below 0.5, showing that restrictions were more frequently of medium intensity, compared to the theoretical maximum. Since ceremonies were never prohibited, nor places of worship closed, nor home-confinement imposed, some fields such as CERE and DIST were never above 0.5–0.6. AC most often fine-tuned NPIs by increasing or decreasing restrictions in INRE, OUTRE, INSP, and OUTSP, while other fields were more constant over time.

**Table 1:** Results of the HMM for the effect of the stringency index overall and in nine fields of activity over the Logarithmic Return (LR_t + 12_) of the weekly COVID-19 incidence growth rate.

Field of activity	Mean index value	Standard Deviation	RR (Median(α_i_))	One-sided bootstrap 95% confidence interval
All fields combined^*^	0.35	0.17	0.78	(0,0.82)
Outdoor sports	0.42	0.24	1.12	(0, 1.32)
Indoor sports	0.48	0.28	0.94	(0, 0.98)
Culture	0.17	0.19	0.86	(0, 0.98)
Ceremonies and religious events	0.30	0.13	0.97	(0, 1.19)
Commerce	0.23	0.16	0.93	(0, 1.02)
Bars and restaurants indoors	0.45	0.32	0.90	(0, 0.95)
Bars and restaurants outdoors	0.29	0.25	0.92	(0, 1.02)
Social distance	0.35	0.11	0.87	(0, 0.95)
Mobility	0.48	0.29	1.02	(0, 1.07)

Results interpretable as Risk Ratio (RR) for the cumulative incidence in the current day vs. 1 week ago for a change in one standard deviation in the stringency index.^*^Simple mean across fields of activity. OUTSP, outdoor sports; INSP, indoor sports; CUL, culture; CERE, ceremonies; COMM, commerce; INRE, indoor bars and restaurants; OUTRE, outdoor bars and restaurants; DIST, social distance; MOBI, mobility; HMM, hierarchical multiplicative model. For every province, in the HMM model, the coefficients 𝛂_i_ = exp.(𝛃_i_).

Results show high heterogeneity in the intensity and type of NPIs imposed in the different Provinces (Supplementary Appendix V). For example, provinces in Castilla-La Mancha had the highest restrictions in INSP, OUTSP, CULT, and CERE, but the lowest in COMM, INRE, OUTRE, and DIST, while others such as Murcia or the Balearic Islands, mainly imposed restrictions in INRE, OUTRE, and DIST. The provinces in Catalonia experienced high stringency in most fields except DIST and CULT, while Madrid scored equally low in all nine fields. High correlation is found between specific fields, such as INSP and OUTSP sports and INRE and OUTRE, with correlation profiles varying by province (Supplementary Appendix VI).

### Effect of NPIs on SARS-CoV-2 transmission

**Figure 3:**
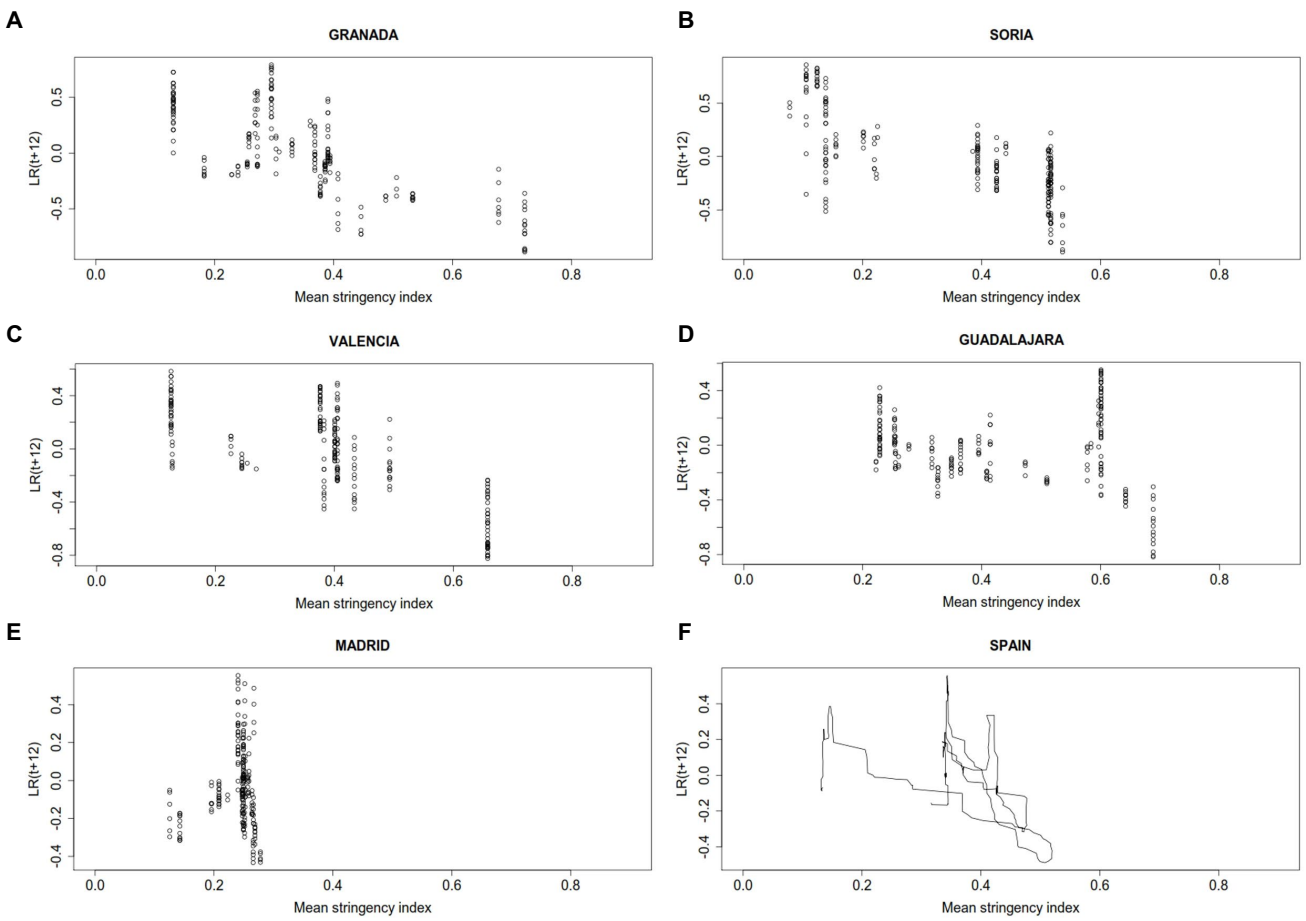
Scatter plots for the mean strigency index and the 7-day COVID-19 logarithmic return of the incidence growth rate 12 days delayed (LRt + 12R) in **(A)** Granada, **(B)** Soria, **(C)** Valencia, **(D)** Guadalajara, **(E)** Madrid, and **(F)** Spain. For the total of Spain consecutive data are joined with segments to show evolution in time.

Scatter plots for the mean stringency index versus the logarithmic 12-day return for 7-day cumulative incidence (LR_*t* + 12_) by province assess visually the association between making NPIs more stringent and decreasing SARS-CoV-2 transmission (Supplementary Appendix VII). There is high variation across provinces, with illustrative cases included in Figure 3. The corresponding correlation coefficients are included in Table 3. While Madrid (***r*** = 0.13) and Guadalajara (***r*** = −0.14) exhibit slightly positive or very moderate negative association, Granada (***r*** = −0.73), Soria (***r*** = −0.72) and Valencia (***r*** = −0.72) show an important negative association. For the whole of Spain the association is negative and quite relevant (***r*** = −0.58).

**Table 3:** Correlation between the variable (LR_t + 12_) and the mean stringency index for every province and for the total of Spain.

Province	Cor(LR_t + 12_, Mean strigency index)
Álava	−0.31
Albacete	−0.29
Alicante	−0.66
Almería	−0.5
Asturias	−0.61
Ávila	−0.53
Badajoz	−0.63
Illes Balears	−0.31
Barcelona	−0.71
Bizkaia	−0.52
Burgos	−0.66
Cáceres	−0.61
Cádiz	−0.51
Cantabria	−0.51
Castellón	−0.62
Ciudad Real	−0.32
Córdoba	−0.37
A Coruña	−0.66
Cuenca	−0.29
Girona	−0.66
Granada	−0.73
Guadalajara	−0.14
Guipuzkoa	−0.54
Huelva	−0.57
Huesca	−0.57
Jaén	−0.65
León	−0.69
Lleida	−0.64
Lugo	−0.6
Madrid	0.13
Málaga	−0.46
Murcia	−0.69
Navarra	−0.62
Ourense	−0.49
Palencia	−0.57
Las Palmas	−0.21
Pontevedra	−0.61
La Rioja	−0.51
Salamanca	−0.59
Santa Cruz de Tenerife	−0.42
Segovia	−0.61
Sevilla	−0.61
Soria	−0.72
Tarragona	−0.67
Teruel	−0.32
Toledo	−0.29
Valencia	−0.72
Valladolid	−0.68
Zamora	−0.57
Zaragoza	−0.48
Spain	−0.58

The estimated coefficients of the HMM for each province are shown in Supplementary Appendix VIII. To summarize the effect of every NPI, median values across provinces, for every coefficient 𝛂_i_, along with one-sided bootstrap 95% confidence intervals (95% CI) have been collected in Table 1. For a one-dimensional model, with just the average stringency index, the effect of NPIs is significant in the median across provinces, with estimated SARS-CoV-2 transmission reduction of 22% while, with 95% confidence, the reduction is, at least, of 18% (RR = 0.78; 95% CI:0, 0.82). In the full model, a significant effect of restrictions on SARS-CoV-2 transmission was found for CULT (RR = 0.86; 95% CI: 0, 0.98), DIST (RR = 0.87; 95% CI: 0, 0.95), INRE (RR = 0.90; 95% CI: 0, 0.95), and INSP (RR = 0.94; 95% CI: 0, 0.98). Of note, the effect of the average stringency index was higher than the effect of any single field of activity, but lower than the total sum of their effects. Using FWER and FDR methods for *p*-value correction to handle multiple testing, we computed the percentage of significant coefficients (𝛂_i_ < 1) (Supplementary Appendix IX). For the significance level 𝛂 =0.05 and using FRD, these percentages are in the range 45–70%.

Since the correlation between INSP and OUTSP, and INRE and OUTRE, was large for the majority of provinces (Supplementary Appendix VI), they were combined to avoid heavy collinearity in the model: SP = (INSP+OUTSP)/2 and RE = (INRE+OUTRE)/2. Results for this new HMM, just considering seven fields, are shown in Table 2. In this model RE (indoor or outdoor) still had a significant effect (RR = 0.88; 95% CI: 0, 0.94) but not SP (indoor or outdoor; RR = 0.97; 95% CI: 0, 1.04). Also DIST consistently showed association with a significant reduction in SARS-CoV-2 transmission (RR = 0.91; 95% CI: 0, 0.99) and a small magnitude effect was found for CERE (RR = 0.95; 95% CI: 0, 0.96).

**Table 2:** Results of the HMM for the effect of the stringency index overall and in seven fields of activity over the Logarithmic Return (LR_t + 12_) of the weekly COVID-19 incidence growth rate.

Field of activity	Mean index value	Standard deviation	RR [Median(α_i_)]	One-sided bootstrap 95% confidence interval
All fields combined^*^	0.34	0.15	0.77	(0, 0.82)
Sports (indoors and outdoors)	0.45	0.25	0.97	(0, 1.04)
Culture	0.17	0.19	1.01	(0, 1.18)
Ceremonies and religious events	0.30	0.13	0.95	(0, 0.96)
Commerce	0.23	0.16	0.89	(0, 1.01)
Bars and restaurants (indoors and outdoors)	0.37	0.26	0.88	(0, 0.94)
Social distance	0.35	0.11	0.91	(0, 0.99)
Mobility	0.48	0.29	1.02	(0, 1.07)

Results interpretable as Risk Ratio (RR) for the cumulative incidence in the current day vs. 1 week ago for a change in one standard deviation in the stringency index.*Simple mean across fields of activity. SP, indoor and outdoor sports; CUL, culture; CERE, ceremonies; COMM, commerce; RE, indoor and outdoor bars and restaurants; DIST, social distance; MOBI, mobility; HMM, hierarchical multiplicative model. For every province, in the HMM model, the coefficients 𝛂_i_ = exp.(𝛃_i_).

## Discussion

Our results show that non-pharmaceutical interventions were effective in decreasing transmission, with an overall estimated decrease in the 7-day cumulative incidence of 22% in the week starting 5 days after an increase in restrictions of 1 standard deviation. Our models assigned the highest and most consistent effects to interventions in social distance and bars and restaurants, particularly indoors, which each decreased incidence by 9–13%, depending on the model. Inconsistent associations with decreased COVID-19 transmission were found for culture and leisure venues, ceremonies and religious celebrations, and indoor sports, possibly explained by a certain collinearity remaining in the model. For any model, no effect of outdoor sports, commerce or mobility restrictions was found in decreasing transmission.

Our study has some limitations. First, the definition of a quantitative index entails discretionary decisions. For example, restrictions in bars and restaurants included different measures, some of them qualitative, such as the prohibition of using the bar or stay standing-up, limitations to number of persons per table, to the capacity or in opening hours. Further, the resulting scale is being compared to NPI applied, for example, to sport activities. Decisions were taken by a panel of expert collaborators and are documented and freely available (22). Moreover, NPIs were graded from a theoretical maximum that in some cases was not achieved. Indexes were normalized to improve comparability, but still, an increase of one standard deviation may not have an equivalent meaning in all fields. Second, there was important correlation between different fields, meaning that they tended to increase or decrease together. This could make it difficult to identify individual effects (8, 27). The attribution of effect to NPIs in specific fields should be done with caution, as it could be sensitive to analytical choices and model specification. Third, we analyzed official restrictions, but not adherence to them, nor precautions decided by individuals on top of existing recommendations. Some studies point to difficulties of the population in understanding complex and changing norms (28, 29), while others show how people may increase precautions and decrease mobility by their own decision (30). Lack of compliance could have underestimated the effectiveness of NPIs, and our results may not be valid for populations with different compliance levels. Of note, our results are specific to a context with low population immunity, either through infection or vaccination, and it is plausible that the effect of NPIs could vary depending on the proportion of susceptible population. Finally, we left out some NPIs, such as measures in betting and gaming venues, or in swimming pools, to avoid noise in the index, since they represent a small fraction of interactions in the field. Any effect of these measures would be spuriously attributed to restrictions implemented simultaneously, for example, in bars and restaurants or in sports facilities in general.

As a strength, the study was conducted over 8 months and three epidemic waves, with on and off measures across 50 territories, providing a rich natural experiment. There were no significant changes in the testing and surveillance recommendations, making the time series reliable. Moreover, many cultural, socioeconomic factors and other contextual variables are shared by the territories, being more homogeneous than international comparisons, and facilitating the attribution of differences to NPIs (13, 31).

Some previous studies have estimated the effectiveness of NPIs in the second and successive COVID-19 epidemic waves. A study in 114 regions of 7 European countries using subnational data and analyzing 17 different NPIs found a combined effect of all NPIs of 66% reduction in the reproduction number Rt (10). However, it evaluated strict measures, such as closure of leisure and entertainment venues, gastronomy, retail and close-contact services, night clubs or educational institutions, with an expected higher impact transmission than the softer restrictions considered in our study. More in line with our results, in Italy, implementation of the less stringent “yellow” tier was associated to decreases in Rt after 3 weeks of 13–19%, the “orange” tier, including closure of restaurants and restrictions to intra-municipality mobility, to 27–38%, and the strictest “red” tier to 36–45% (15). However, when measures are implemented in tiered levels, it is difficult to disentangle the relative contribution of the different NPIs (13–15).

Regarding the effectiveness of specific NPIs, in Switzerland, business closures, recommendations to work from home and restrictions on gatherings were particularly effective (32). In 7 European countries (10), closing non-essential business had the highest effect, decreasing COVID-19 transmission by 35% (including night clubs, restaurants, retail and close-contact services and, leisure and entertainment venues). Limiting gatherings to 2 people reduced transmission by 26%, and curfew by 13%. A study in Spain in 7 provinces, also found no effect of regional mobility restrictions but found that more strict curfews were associated with increased transmission (21). However, the range of NPIs included was limited, making spurious associations a greater threat. This same study found an effect of limiting gatherings, the intervention that is found associated with decreased transmission more consistently (in our study, social distance decreased transmission between 9 and 13%).

In conclusion, our results indicate that increasing restrictions had a considerable effect in decreasing COVID-19 transmission, with interventions in social distance, bars and restaurants having the higher and more consistent effect. Our results can contribute to the corpus of evidence that will help inform future decisions in response to COVID-19 resurgence or to future pandemics. However, the mortality and morbidity preventable by the implementation of NPIs, will also depend on the severity of the averted infections, which needs to be balanced against the potential negative effects of NPIs. Continued partnership and collaboration between epidemiologists in the public administration and scientists, particularly mathematicians and data scientists, is crucial to ensure adequate and timely analysis of data that can be used for evidence-based recommendations.

## Data availability statement

The datasets presented in this study can be found in online repositories. The names of the repository/repositories and accession number(s) can be found below: https://npispain.org.

## Ethics statement

Ethical review and approval was not required for the study on human participants in accordance with the local legislation and institutional requirements. Written informed consent for participation was not required for this study in accordance with the national legislation and the institutional requirements.

## Members of the Study Group for Non-Pharmaceutical Interventions in Spain

Adrian Abeal, Francisco Amor, Elena Plans, Isabel Peya, José Cuesta, Pedro Álvarez, Fernando Simón, Alfonso Gordaliza, Manuel Oviedo, Adriana Soto, Paola Sánchez, Francisco Arenal, Adrián Aginagalde-Llorente, Jacobo Mendioroz, Eva Martínez Ochoa, Magdalena Salom Castell, Juan Pablo Alonso, María-Dolores Chirlaque, Fernando González Carril, Nicola Lorusso, María del Carmen Pacheco, Pilar Peces Jiménez.

## Author contributions

RC, SM, DG-U, MJS, and SV-A conceived the idea and initiated the study. SFB, BV, SM, and members of the collective author defined the NPI coding book, and operationally defined the stringency index. SFB, SM, and members of the collective author collected and validated the NPI data. IB and DP analyzed the data under the supervision of RC and DG-U. RC, IB, and SM wrote the first draft with inputs from DP and DG-U. All authors participated in the interpretation of results and critically reviewed the content of the manuscript, read and approved the final version of the manuscript.

## Funding

This work has been partially supported by GAIN (Galician Innovation Agency) and the Regional Ministry of Economy, Employment and Industry, Xunta de Galicia IN845D 2020/26 (Grant COV20/00604) through the ERDF (2014-2020). The research of DGU has been financed in part by the Spanish Agencia Estatal de Investigación under grants PID2021-122154NB-I00 and TED2021-129455B-I00, and by a 2021 BBVA Foundation project for research in Mathematics. He also acknowledges support from the EU under the 2014-2020 ERDF Operational Programme and the Department of Economy, Knowledge, Business and University of the Regional Government of Andalusia (FEDER-UCA18-108393).

## Conflict of interest

The authors declare that the research was conducted in the absence of any commercial or financial relationships that could be construed as a potential conflict of interest.

## Publisher’s note

All claims expressed in this article are solely those of the authors and do not necessarily represent those of their affiliated organizations, or those of the publisher, the editors and the reviewers. Any product that may be evaluated in this article, or claim that may be made by its manufacturer, is not guaranteed or endorsed by the publisher.

## Supplementary material

The Supplementary material for this article can be found online at: https://www.frontiersin.org/articles/10.3389/fpubh.2023.1061331/full#supplementary-material
