# Supplementary material for: Effectiveness of non-pharmaceutical interventions in nine fields of activity to decrease SARS-CoV-2 transmission (Spain, September 2020–May 2021)
Source: Front Public Health. 2023 Apr 12;11:1061331. doi: 10.3389/fpubh.2023.1061331 (PMC10131688; doi:10.3389/fpubh.2023.1061331)
Supplement: Supplementary file 1 [file Data_Sheet_1.PDF]

Supplementary material

Effectiveness of non-pharmaceutical interventions in nine fields of activity to decrease SARS-CoV-2 transmission (Spain, September 2020 - May 2021)

Inés Barbeito, Daniel Precioso, David Goméz-Ullate, María José Sierra, Susana Vegas, Sonia Fernández Balbuena, Begoña Vitoriano, Susana Monge*, Ricardo Cao* and the Study Group for Non-Pharmaceutical Interventions in Spain**

Contents

Appendix I. Dictionary of coded non-pharmaceutical interventions. **2**

Appendix II. Creation of the stringency index in each of the 9 fields of activity, based on values assigned to coded Non-Pharmaceutical Interventions and weighting criteria. **4**

Appendix III. Taxonomy of Non-Pharmaceutical Interventions and weighting criteria. **5**

Appendix IV. Boxplot of the distribution across the 50 Spanish Provinces of the mean, median, standard deviation, interquartile range and coefficient of variation of the stringency index between 15 September 2021 and 9 May 2022, by field of activity. **14**

Appendix V. Radar charts depicting restriction level by field of activity (in the different axis) between 15 September 2021 and 9 May 2022 in Spanish provinces: (a) template for the median, numbers in blue are the range endpoints for every field (b) and radar plots showing the median stringency index by province, normalized to the values in the template, so the level of restriction on each field is relative to the maximum and minimum median across all provinces. **15**

Appendix VI. Correlation matrices between stringency indices of fields by province. **20**

Appendix VII. Scatter plots for the mean strigency index and the 7-day COVID-19 logarithmic return of the incidence growth rate 12 days delayed (LR_{t+12}) in each province and Spain. **36**

Appendix VIII. Estimated coefficients for the hierarchical multiplicative model (HMM) considered. **62**

Appendix IX. Percentage of significant coefficients (along provinces) for every stringency index using classical methods as well as methods for controlling the family-wise error rate (FWER) and the false discovery rate (FDR). **63**

Appendix I. Dictionary of coded non-pharmaceutical interventions

Code*	Description
**Field of activity: Sports**	
AF.1	General closure of sport centres/installations
AF.2	General closure of indoor (but not indoor) sport centres/installations
AF.12	Limitations to practising sports in groups indoors
AF.7	Limitations to practising sports in groups outdoors
AF.5	Limitations to capacity of sports centres indoors
AF.6	Limitations to capacity of sport centres outdoors
AF.4	Prohibition of sport activities involving physical contact
AF.17	Limits to sport activities involving physical contact
AF.3	Cancelation of sport events
AF.13	Prohibition of public in sport events outdoors
AF.14	Prohibition of public in sport events indoors
AF.15	Limits to the public capacity in sport events outdoors
AF.16	Limits to the public capacity in sport events indoors
**Field of activity: Culture**	
CD.1	Closure of monuments, museums, libraries etc.
CD.2	Closure of indoor (but not outdoor) monuments, museums, libraries etc.
CD.6	Capacity limitations of monuments, museums, libraries etc.
CD.7	Capacity limitations of indoor monuments, museums, libraries etc.
CD.8	Capacity limitations of outdoor monuments, museums, libraries etc.
CD.3	Closure of cinemas, theatres, circus and similar spaces for cultural spectacles
CD.4	Closure of indoor (but not outdoor) cinemas, theatres, circus and similar spaces for cultural spectacles
CD.9	Capacity limitations of cinemas, theatres, circus and similar spaces for cultural spectacles indoors
CD.10	Capacity limitations of cinemas, theatres, circus and similar spaces for cultural spectacles outdoors
CD.16	Closure of zoos, amusement parks and similar
CD.15	Capacity limitations of zoos, amusement parks and similar
CD.17	Closure of bullfighting shows
CD.14	Capacity limitations of bullfighting shows
CD.11	Closure of multipurpose rooms
CD.5	Capacity limitations of multipurpose rooms
**Field of activity: Ceremonies and religious celebrations**	
CE.1	Closure of religious temples (theoretical, never used)
CE.2	Capacity limitations of religious temples
CE.7	Prohibition of choirs at religious events
CE.3	Capacity limitations in funeral ceremonies indoors
CE.5	Capacity limitations in other ceremonies (weddings, baptisms) indoors
CE.4	Capacity limitations in funeral ceremonies outdoors
CE.6	Capacity limitations in other ceremonies (weddings, baptisms) outdoors
CE.9	Funeral ceremonies restricted to household members
CE.10	Other ceremonies (weddings, baptisms) restricted to household members
**Field of activity: Commerce**	
CO.1	Closure of all non-essential commercial activity
CO.8	Capacity limitations in commercial activity, stores etc
CO.7	Limitation in the opening times for commercial activity, stores etc
CO.2	Closure of high-capacity stores (i.e. ¿400 m2)
CO.3	Prohibition of commercial activities involving physical contact
CO.4	Closure of malls
CO.5	Closure only of common areas within malls
CO.9	Capacity limitations of malls (if different from general commercial activity)
CO.6	Closure of open space markets
CO.10	Capacity limitations of open space markets

Code*	Description
**Field of activity: Mobility**	
MV.3	Curfew
MV.4	Perimeter entry/exit restriction
MV.7	Mobility restriction within smaller áreas in the territory (i.e. between municipalities)
**Field of activity: bars and restaurants**	
RH.1	Total closure of bars and restaurants
RH.2	Bars and restaurants only open for take away
RH.3	Closure of indoor (but not outdoor) spaces in bars and restaurants
RH.4	Prohibition of eating or drinking at the bar or standing up
RH.7	Capacity limitations indoors in bars and restaurants
RH.6	Capacity limitations outdoors in bars and restaurants
RH.9	Maximum number of persons per tables in bars and restaurants in general
RH.11	Maximum number of persons per tables in bars and restaurants indoors
RH.10	Maximum number of persons per tables in bars and restaurants outdoors
RH.5	Opening time limitations in bars and restaurants
**Field of activity: social distance**	
MV.1	Home confinement (theoretical, never used in this period)
MV.2	Recommendation to stay at home except for essential activities
RS.1	Limitations to gathering of people (except household members) everywhere
RS.2	Limitations to gathering of people (except household members) in public spaces
RS.3	Limitations to gathering of people (except household members) in public spaces indoors (but not outdoors)
RS.8	Restriction to interaction with household members
TP.1	Capacity limitation in public transport
CD.12	Limitation in the maximum number of persons allowed in an event
CD.13	Need for authorization from public health authorities for events of more than a limit of persons

Appendix II. Creation of the stringency index in each of the 9 fields of activity, based on values assigned to coded Non-Pharmaceutical Interventions and weighting criteria.

In this section we describe the procedure to create a daily stringency index for each of the 9 fields of activity for each of the 52 provinces of Spain. Stringency indices are designed to take values between 0 and 1, where 0 indicates no measures taken and 1 the highest level of restriction. The first step in the data collection has been to define a list of non-pharmaceutical interventions (NPIs) in each of these 9 fields of activity, which are shown in Appendix I. Some of these NPIs have parameters that reflect their intensity. For instance, the measure RH.7 "Capacity limitations indoors in bars and restaurants" must be accompanied by an extra parameter specifying the percentage of the total capacity of a restaurant whose occupation was allowed while this measure was applied. Likewise, intervention CO.7 "Limitation in the opening times for commercial activity, stores etc" must be accompanied by the time interval where shops were allowed to open. During the period under study, some of these NPIs were decided by the National Government and thus applicable over the whole country, while others applied at the Autonomous Community (AC) level, at provincial level or even applicable only to certain towns and cities within a province. A considerable amount of work was carried out by the Study Group for Non-Pharmaceutical Interventions in Spain to read all the legal bulletins published at National and AC levels in order to gather, categorise and properly encode each NPI according to the taxonomy described in Appendix III. The resulting set of data with all the encoded NPIs is accessible at the companion website (http://npispain.clapton.uca.es//downloads). The steps towards building a stringency index for each field of activity on a given day at a given province are the following:

1. Apply the NPI rules in the taxonomy to an NPI (with its corresponding modifying parameters) to score its value of restriction as high (1), medium (0.5) or low (0.2). The numerical values for these levels of intensity are given in brackets. NPIs that were not enforced on a province on a given day take the value 0.

2. Combine single NPIs into items according to the Item rules described in the taxonomy. An item gathers a number of possible NPIs referred to the same activity.

3. Finally, all of the items in each of the 9 fields of activity are combined with certain weights that reflect the relative importance of the activity item in the whole field of activity.

When NPIs of different restrictive strength have been applied on different regions within a province, the stringency index for the province has been calculated by a weighted average of the different regions according to the fraction of the province's population affected by each NPI. Similarly, the raw data contains NPIs applied in each island of the Canary and Balearic Islands, that have been aggregated at provincial level, with average weighted by population. The systematic application of the above rules allows to convert all the NPIs taken at subprovincial, provincial, Autonomous Community and country level into a single stringency index for each field of activity that varies across provinces and changes on a daily basis. The processed set of data containing the time series for all stringency indices can be downloaded from the companion website, where other visualisations and exploratory data analysis of stringency indices are also available. In order to render the whole process reproducible for other scholars, not only the raw and processed data, but also the python code that performs all necessary manipulations has been made available at the following git repository https://github.com/UCA-Datalab/covid-npi

Appendix III. Taxonomy of Non-Pharmaceutical Interventions and weighting criteria.

Field of activity: Outdoor sports					
**Code**	**NPI**	**Item**	**Item rules**	**Item weight**	**NPI rules**
AF.1	General closure of sport centres	DEX_afor	max(AF.1, AF.6, AF.7)	1	If applied: high
AF.6	Limitations to capacity of sport centres outdoors				If capacity <=35%: medium >35%: low
AF.7	Limitations to practising sports in groups outdoors				If n.people <=6 : medium >6 : low
AF.4	Prohibition of sport activities involving physical contact	DP_cont	max(AF.4, AF.17)	0.2	If applied: high
AF.17	Limits to sport activities involving physical contact				If n.people <=6 : medium >6 : low
AF.3	Cancelation of sport events	DEX_pub	max(AF.3, AF.13, AF.15)	0.7	If applied: high
AF.13	Prohibition of public in sport events outdoors				If applied: high
AF.15	Limits to the public capacity in sport events outdoors				If capacity <=35%: medium >35%: low

Field of activity: Indoor Sports					
**Code**	**NPI**	**Item**	**Item rules**	**Item weight**	**NPI rules**
AF.1	General closure of sport centres/installations	DIN_afo	max(AF.1, AF.2, AF.5, AF.12)	1	If applied: **high**
AF.2	General closure of indoor (but not outdoor) sport centres/installations				If applied: **high**
AF.5	Limitations to capacity of sport centres indoors				(Take highest) If capacity <=35%: **medium** >35%: **low** If n.people <=6 : **medium** >6 : **low**
AF.12	Limitations to practising sports in groups indoors				If n.people <=6: **medium**
AF.4	Prohibition of sport activities involving physical contact	DIN_grupo	max(AF.4, AF.17)	0.2	If applied: **high**
AF.17	Limits to sport activities involving physical contact				If n.people <=6 : **medium** >6 : **low**
AF.3	Cancelation of sport events	DIN_pub	max(AF.3, AF.14, AF.16)	0.7	If applied: **high**
AF.14	Prohibition of public in sport events indoors				If applied: **high**
AF.16	Limits to the public capacity in sport events indoors				If capacity <=35%: **medium**

Field of activity: Culture					
**Code**	**NPI**	**Item**	**Item rules**	**Item weight**	**NPI rules**
CD.1	Closure of monuments, museums, libraries etc.	CUL_mus	max(CD.1, mean(max(CD.2, CD.6, CD.7), max(CD.6, CD.8)))	0.5	If applied: high
CD.6	Capacity limitations of monuments, museums, libraries etc.				If capacity <=35%: medium
CD.2	Closure of indoor (but not outdoor) monuments, museums, libraries etc.				If applied: high
CD.7	Capacity limitations of indoor monuments, museums, libraries etc.				If capacity <=35%: medium
CD.8	Capacity limitations of outdoor monuments, museums, libraries etc.				If capacity <=35%: medium
CD.3	Closure of cinemas, theatres, circus and similar spaces for cultural spectacles	CUL_cin	mean(max(CD.3, CD.4, CD.9), CD.10)	1	If applied: high
CD.4	Closure of indoor (but not outdoor) cinemas, theatres, circus and similar spaces for cultural spectacles				If applied: high
CD.9	Capacity limitations of cinemas, theatres, circus and similar spaces for cultural spectacles indoors				If capacity <=35%: medium
CD.10	Capacity limitations of cinemas, theatres, circus and similar spaces for cultural spectacles outdoors				If capacity <=35%: medium
CD.5	Closure of multipurpose rooms	CUL_sal	max(CD.5, CD.11)	0.3	If applied: high
CD.11	Capacity limitations of multipurpose rooms				If capacity <=35%: medium
CD.17	Closure of bullfighting shows	CUL_tor	max(CD.3, CD.14, CD.17)	0.2	If applied: high
CD.14	Capacity limitations of bullfighting shows				If capacity <=35%: medium
CD.16	Closure of zoos, amusement parks and similar	CUL_zoo	max(CD.3, CD.15, CD.16)	0.2	If applied: high
CD.15	Capacity limitations of zoos, amusement parks and similar				If capacity <=35%: medium

Field of activity: Ceremonies					
**Code**	**NPI**	**Item**	**Item rules**	**Item weight**	**NPI rules**
CE.1	Closure of religious temples (theoretical, never used)	CER_cult	max(CE.1, CE.2)	1	If applied: **high**
CE.2	Capacity limitations of religious temples				If capacity<br><=35%: **medium**
CE.7	Prohibition of choirs at religious events	CER_cor	CE.7	0.2	If applied: **high**
CE.3	Capacity limitations in funeral ceremonies indoors	CER_ent_int	max(CE.3, CE.9)	0.3	(Take highest)<br>If capacity<br><=35%: **medium**<br>>35%: **low**<br>If n.people<br><=10 : **medium**<br>>10 : **low**
CE.4	Capacity limitations in funeral ceremonies outdoors	CER_ent_ext	max(CE.4, CE.9)	0.2	If capacity<br><=35%: **medium**<br>If n.people<br><=10 : **medium**
CE.9	Funeral ceremonies restricted to household members				If applied: **high**
CE.5	Capacity limitations in other ceremonies (weddings, baptisms) indoors	CER_otro_int	max(CE.5, CE.10)	0.3	(Take highest)<br>If capacity<br><=35%: **medium**<br>>35%: **low**<br>If n.people<br><=10 : **medium**<br>>10 : **low**
CE.10	other ceremonies (weddings, baptisms) restricted to household members				If applied: **high**
CE.6	Capacity limitations in other ceremonies (weddings, baptisms) outdoors	CER_otro_ext	max(CE.6, CE.10)	0.2	If capacity<br><=35%: **medium**<br>If n.people<br><=10 : **medium**

Field of activity: Commerce

Code	NPI	Item	Item rules	Item weight	NPI rules
CO.1	Closure of all non-essential commercial activity	COM_afo	max(CO.1, CO.8)	1	If applied: **high**
CO.8	Capacity limitations in commercial activity, stores etc				If capacity <=35%: **medium**
CO.7	Limitation in the opening times for commercial activity, stores etc	COM_hor	max(CO.1, CO.7)	0.6	If time <= 18h: **medium** > 18h: **low**
CO.2	Closure of high-capacity stores (i.e. >400 m2)	COM_esp	max(CO.1, CO.2)	0.3	If applied: **high**
CO.3	Prohibition of commercial activities involving physical contact	COM_fis	max(CO.1, CO.4, CO.8, CO.9)	0.2	If applied: **high**
CO.4	Closure of malls	COM_cent	max(CO.4, CO.9)	1	If applied: **high**
CO.9	Capacity limitations of malls (if different from general commercial activity)				If capacity <=35%: **medium** >35%: **low**
CO.5	Closure only of common areas within malls	COM_cczon	max(CO.6, CO.10)	0.3	If applied: **high**
CO.6	Closure of open space markets	COM_libre		0.4	If applied: **high**
CO.10	Capacity limitations of open space markets				If capacity <=35%: **medium**

Field of activity: Indoor bars and restaurants					
**Code**	**NPI**	**Item**	**Item rules**	**Item weight**	**NPI rules**
RH.1	Total closure of bars and restaurants	REX_afo	max(RH.1, RH.2, RH.6)	1	If applied: **high**
RH.2	Bars and restaurants only open for take away				If applied: **high**
RH.6	Capacity limitations outdoors in bars and restaurants				If capacity <=35%: **medium**
RH.5	Opening time limitations in bars and restaurants	REX_hor	max(RH.1, RH.2, RH.5)	0.6	If time <= 18h: **medium** > 18h: **low**
RH.9	Maximum number of persons per tables in bars and restaurants in general	REX_otr	max(RH.1, RH.2, RH.9, RH.10)	0.6	If n.people <=6 : **medium** >6 : **low**
RH.10	Maximum number of persons per tables in bars and restaurants outdoors				If n.people <=6 : **medium** >6 : **low**

Field of activity: Outdoors bars and restaurants					
**Code**	**NPI**	**Item**	**Item rules**	**Item weight**	**NPI rules**
RH.1	Total closure of bars and restaurants	RIN_afo	max(RH.1, RH.2, RH.3, RH.4 + RH.7)	1	If applied: **high**
RH.2	Bars and restaurants only open for take away				If applied: **high**
RH.3	Closure of indoor (but not outdoor) spaces in bars and restaurants				If applied: **high**
RH.4	Prohibition of eating or drinking at the bar or standing up				If applied: **low**
RH.7	Capacity limitations indoors in bars and restaurants				If capacity <=35%: **medium**
RH.5	Opening time limitations in bars and restaurants	RIN_hor	max(RH.1, RH.5)	0.6	If time <= 18h: **medium** > 18h: **low**
RH.9	Maximum number of persons per tables in bars and restaurants in general	RIN_mesa	max(RH.1, RH.2, RH.3, RH.9, RH.11)	0.6	If n.people <=6 : **medium** >6 : **low**
RH.11	Maximum number of persons per tables in bars and restaurants indoors				If n.people <=6 : **medium** >6 : **low**

Field of activity: Social distance					
**Code**	**NPI**	**Item**	**Item rules**	**Item weight**	**NPI rules**
CD.12	Limitation in the maximum number of persons allowed in an event	DS_even	max(CD.12, CD.13, MV.1)	0.6	If n.people <=100 : **medium** >100 : **low**
CD.13	Need for authorization from public health authorities for events of more than a limit of persons				If n.people <=100 : **medium**
MV.1	Home confinement (theoretical, never used in this period)	DS_dom	max(MV.1, MV.2)	1	If applied: **high**
MV.2	Recommendation to stay at home except for essential activities				If applied: **low**
RS.1	Limitations to gathering of people (except household members) everywhere	DS_reun	max(RS.1, RS.2, RS.3, RS.8)	0.8	If n.people <=6 : **high**
RS.2	Limitations to gathering of people (except household members) in public spaces				If n.people <=6 : **high** >6 and <=10 : **medium** >10 : **low**
RS.3	Limitations to gathering of people (except household members) in public spaces indoors (but not outdoors)				If n.people <=6 : **high**
RS.8	Restriction to interaction with household members				If applied: **high**
TP.1	Capacity limitation in public transport	DS_tran	max(MV.1, TP.1)	0.2	If applied: **high**

Field of activity: Mobility					
**Code**	**NPI**	**Item**	**Item rules**	**Item weight**	**NPI rules**
MV.3	Mobility restriction at night (curfew)	MOV_qued	MV.3	1	If applied: **high**
MV.4	Perimeter entry/exit restriction	MOV_per	MV.4	0.4	If applied: **high**
MV.7	Mobility restriction within smaller areas in the territory (i.e. between municipalities)	MOV_int	max(MV.4, MV.7)	1	If applied: **high**

Appendix IV. Boxplot of the distribution across the 50 Spanish Provinces of the mean, median, standard deviation, interquartile range and coefficient of variation of the stringency index between 15 September 2021 and 9 May 2022, by field of activity.

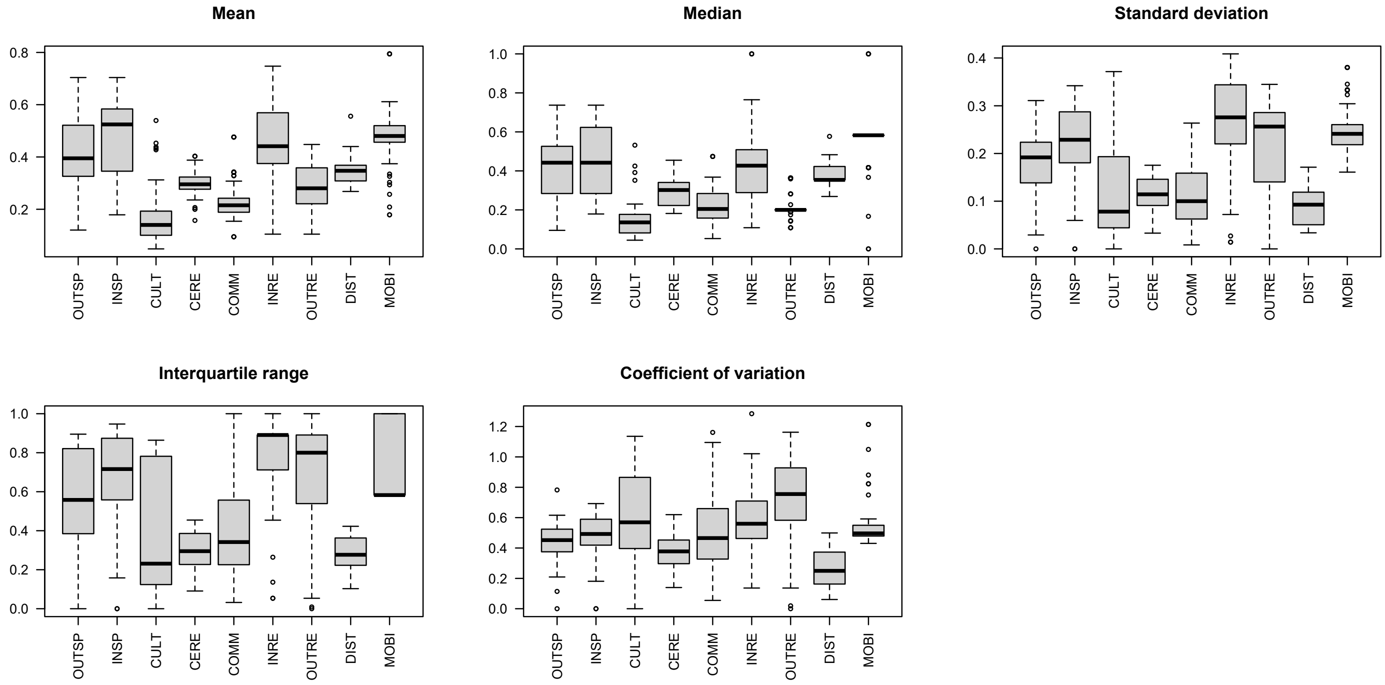

OUTSP= Outdoor sports; INSP= Indoor sports; CULT= culture; CERE= ceremonies; COMM= commerce; INRE= Indoor bars and restaurants; OUTRE= Outdoor bars and restaurants; DIST=Social Distance; MOBI= Mobility

Appendix V. Radar charts depicting restriction level by field of activity (in the different axis) between 15 September 2021 and 9 May 2022 in Spanish provinces: (a) template for the median, numbers in blue are the range endpoints for every field (b) and radar plots showing the median stringency index by province, normalized to the values in the template, so the level of restriction on each field is relative to the maximum and minimum median across all provinces.

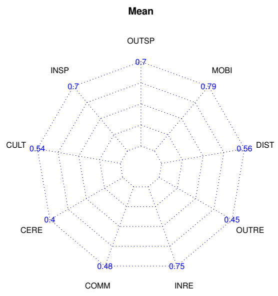

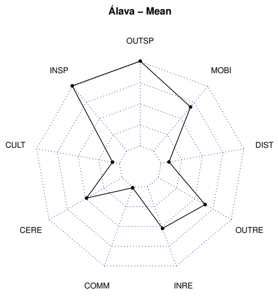

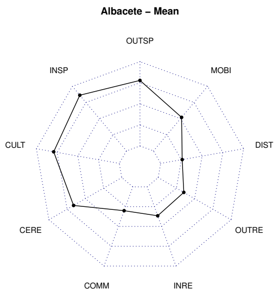

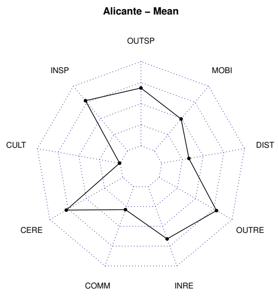

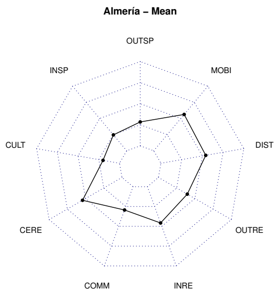

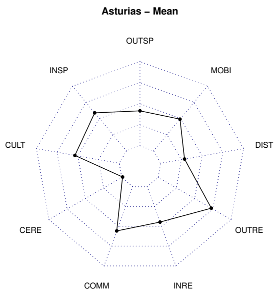

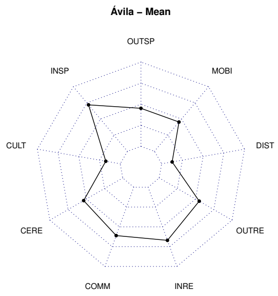

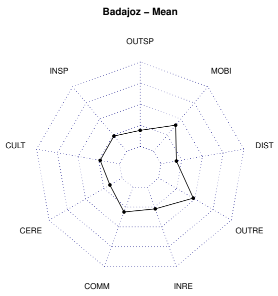

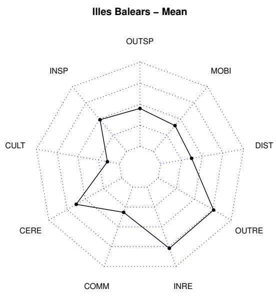

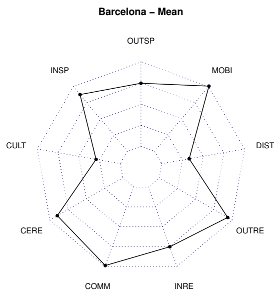

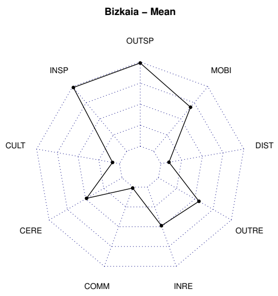

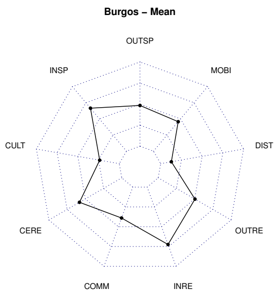

Cáceres – Mean

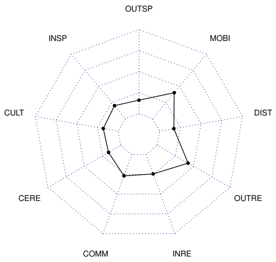

Cádiz – Mean

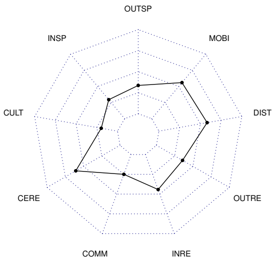

Cantabria – Mean

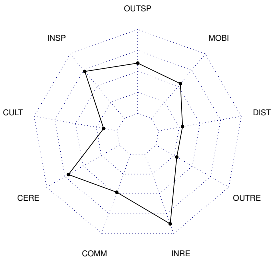

Castellón – Mean

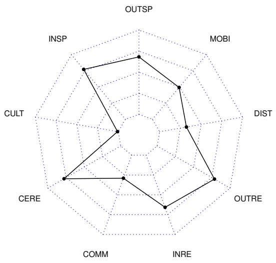

Ciudad Real – Mean

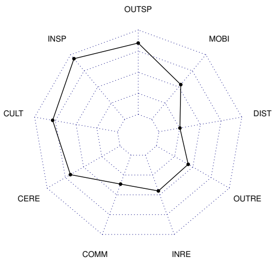

Córdoba – Mean

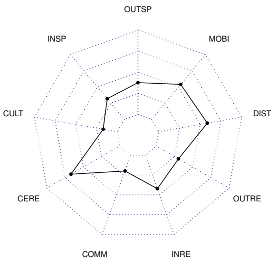

A Coruña – Mean

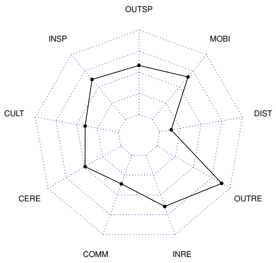

Cuenca – Mean

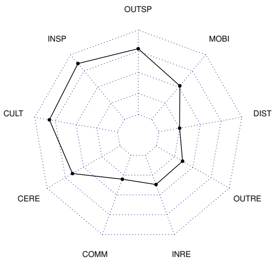

Girona – Mean

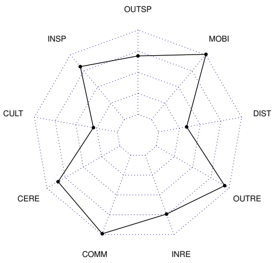

Granada – Mean

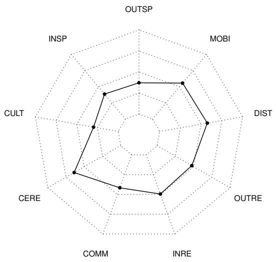

Guadalajara – Mean

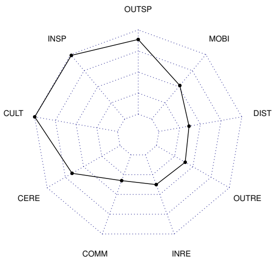

Guipuzkoa – Mean

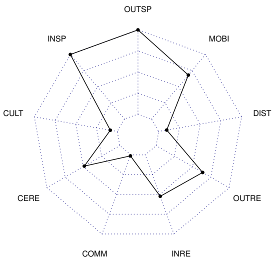

Huelva – Mean

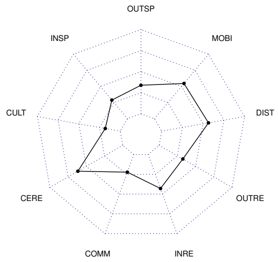

Huesca – Mean

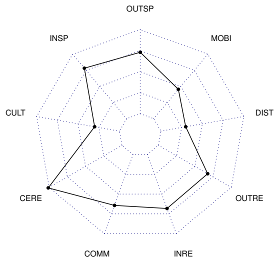

Jaén – Mean

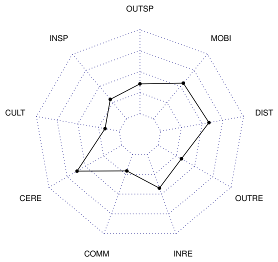

León – Mean

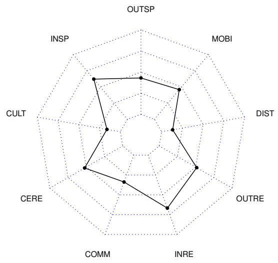

Lleida – Mean

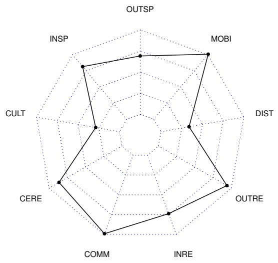

Lugo – Mean

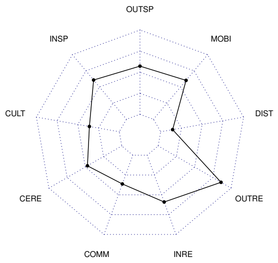

Madrid – Mean

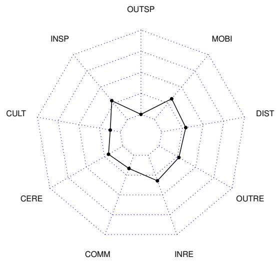

Málaga – Mean

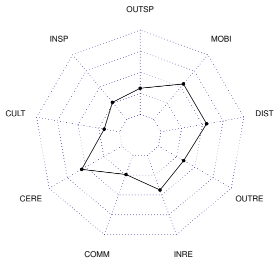

Murcia – Mean

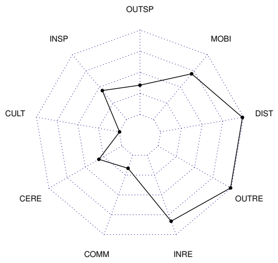

Navarra – Mean

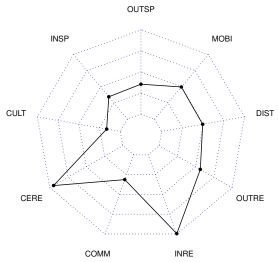

Ourense – Mean

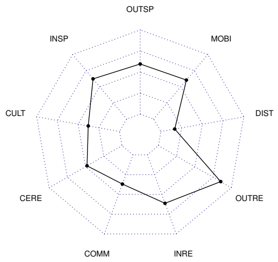

Palencia – Mean

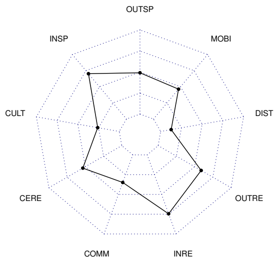

Las Palmas – Mean

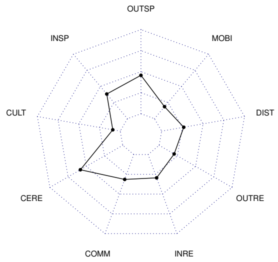

Pontevedra – Mean

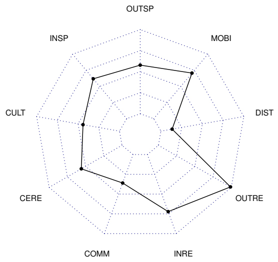

La Rioja – Mean

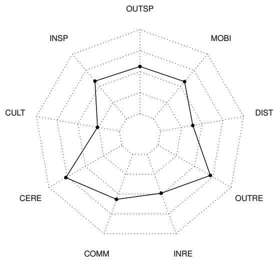

Salamanca – Mean

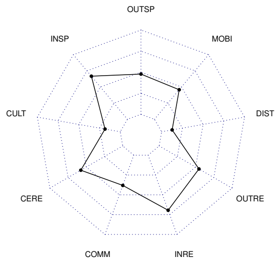

Santa Cruz de Tenerife – Mean

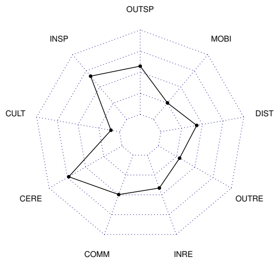

Segovia – Mean

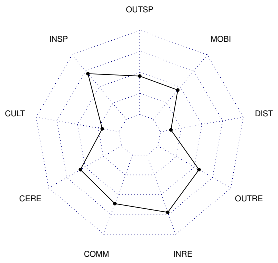

Sevilla – Mean

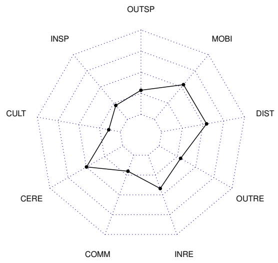

Soria – Mean

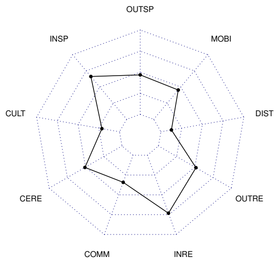

Tarragona – Mean

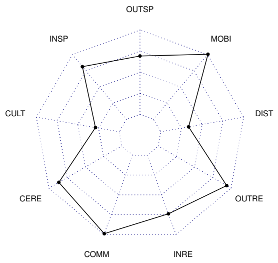

Teruel – Mean

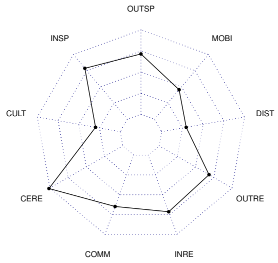

Toledo – Mean

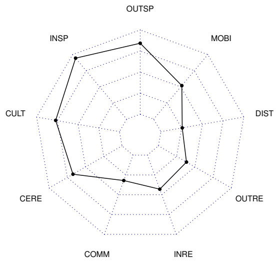

Valencia – Mean

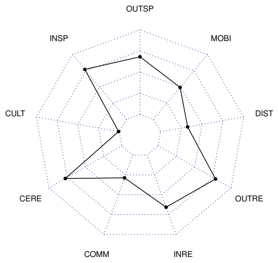

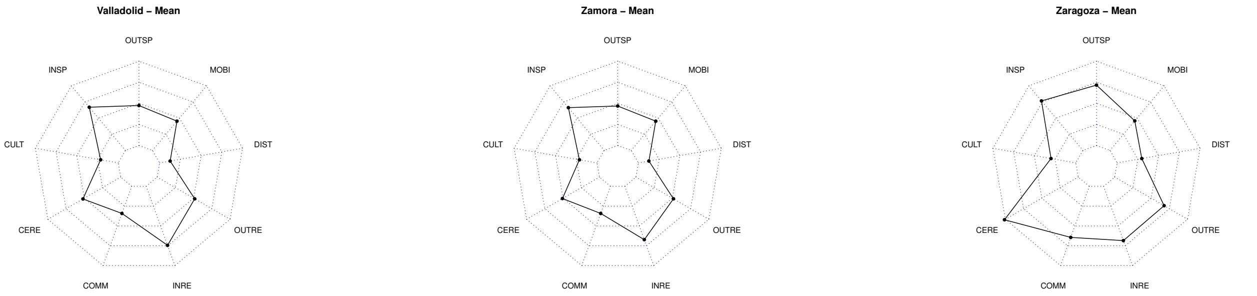

OUTSP= Outdoor sports; INSP= Indoor sports; CULT= culture; CERE= ceremonies; COMM= commerce; INRE= Indoor bars and restaurants; OUTRE= Outdoor bars and restaurants; DIST=Social Distance; MOBI= Mobility

Appendix VI. Correlation matrices between stringency indices of fields by province.

In green, correlations which turned out to be higher or equal to 0.9 ($\rho \geq 0.90$). In blue, those correlations higher or equal to 0.80 and smaller than 0.90 ($0.80 < \rho \leq 0.90$). In red, those correlations smaller or equal to -0.90 ($\rho \leq -0.90$). Finally, in orange, those correlations smaller or equal to -0.80 and higher than -0.90 ($-0.90 < \rho \leq -0.80$).

	OUTSP	INSP	CULT	CERE	COMM	INRE	OUTRE	DIST	MOBI
OUTSP	1	0.93	0.77	0.65	0.88	0.74	0.66	0.87	0.83
INSP	0.93	1	0.89	0.81	0.90	0.77	0.81	0.72	0.75
CULT	0.77	0.89	1	0.83	0.90	0.82	0.82	0.59	0.66
CERE	0.65	0.81	0.83	1	0.74	0.89	0.89	0.49	0.69
COMM	0.88	0.90	0.90	0.74	1	0.74	0.70	0.76	0.68
INRE	0.74	0.77	0.82	0.89	0.74	1	0.84	0.69	0.81
OUTRE	0.66	0.81	0.82	0.89	0.70	0.84	1	0.55	0.68
DIST	0.87	0.72	0.59	0.49	0.76	0.69	0.55	1	0.80
MOBI	0.83	0.75	0.66	0.69	0.68	0.81	0.68	0.80	1

Supplementary Table 1: Crossed correlations among the 9 fields in A Coruña.

	OUTSP	INSP	CULT	CERE	COMM	INRE	OUTRE	DIST	MOBI
OUTSP	1	0.93	0.80	0.67	0.89	0.84	0.73	0.87	0.92
INSP	0.93	1	0.90	0.83	0.91	0.89	0.88	0.71	0.84
CULT	0.80	0.90	1	0.79	0.93	0.83	0.84	0.58	0.67
CERE	0.67	0.83	0.79	1	0.76	0.93	0.93	0.48	0.68
COMM	0.89	0.91	0.93	0.76	1	0.84	0.75	0.76	0.76
INRE	0.84	0.89	0.83	0.93	0.84	1	0.94	0.72	0.84
OUTRE	0.73	0.88	0.84	0.93	0.75	0.94	1	0.56	0.74
DIST	0.87	0.71	0.58	0.48	0.76	0.72	0.56	1	0.86
MOBI	0.92	0.84	0.67	0.68	0.76	0.84	0.74	0.86	1

Supplementary Table 2: Crossed correlations among the 9 fields in Lugo.

	OUTSP	INSP	CULT	CERE	COMM	INRE	OUTRE	DIST	MOBI
OUTSP	1	0.93	0.81	0.64	0.89	0.71	0.59	0.87	0.82
INSP	0.93	1	0.90	0.77	0.91	0.70	0.71	0.72	0.72
CULT	0.81	0.90	1	0.77	0.89	0.75	0.77	0.64	0.66
CERE	0.64	0.77	0.77	1	0.68	0.88	0.83	0.50	0.73
COMM	0.89	0.91	0.89	0.68	1	0.64	0.59	0.77	0.61
INRE	0.71	0.70	0.75	0.88	0.64	1	0.86	0.69	0.89
OUTRE	0.59	0.71	0.77	0.83	0.59	0.86	1	0.53	0.70
DIST	0.87	0.72	0.64	0.50	0.77	0.69	0.53	1	0.80
MOBI	0.82	0.72	0.66	0.73	0.61	0.89	0.70	0.80	1

Supplementary Table 3: Crossed correlations among the 9 fields in Pontevedra.

	OUTSP	INSP	CULT	CERE	COMM	INRE	OUTRE	DIST	MOBI
OUTSP	1	0.93	0.81	0.66	0.89	0.75	0.68	0.88	0.87
INSP	0.93	1	0.90	0.82	0.91	0.79	0.82	0.72	0.82
CULT	0.81	0.90	1	0.81	0.92	0.80	0.80	0.59	0.74
CERE	0.66	0.82	0.81	1	0.75	0.91	0.90	0.46	0.70
COMM	0.89	0.91	0.92	0.75	1	0.72	0.68	0.72	0.72
INRE	0.75	0.79	0.80	0.91	0.72	1	0.90	0.65	0.86
OUTRE	0.68	0.82	0.80	0.90	0.68	0.90	1	0.54	0.77
DIST	0.88	0.72	0.59	0.46	0.72	0.65	0.54	1	0.82
MOBI	0.87	0.82	0.74	0.70	0.72	0.86	0.77	0.82	1

Supplementary Table 4: Crossed correlations among the 9 fields in Ourense.

	OUTSP	INSP	CULT	CERE	COMM	INRE	OUTRE	DIST	MOBI
OUTSP	1	0.92	0.91	0.35	0.84	0.89	0.93	0.46	0.35
INSP	0.92	1	0.92	0.44	0.82	0.98	0.98	0.53	0.45
CULT	0.91	0.92	1	0.40	0.78	0.89	0.93	0.44	0.41
CERE	0.35	0.44	0.40	1	0.32	0.38	0.33	0.51	0.98
COMM	0.84	0.82	0.78	0.32	1	0.80	0.80	0.62	0.30
INRE	0.89	0.98	0.89	0.38	0.80	1	0.95	0.47	0.39
OUTRE	0.93	0.98	0.93	0.33	0.80	0.95	1	0.49	0.34
DIST	0.46	0.53	0.44	0.51	0.62	0.47	0.49	1	0.41
MOBI	0.35	0.45	0.41	0.98	0.30	0.39	0.34	0.41	1

Supplementary Table 5: Crossed correlations among the 9 fields in Asturias.

	OUTSP	INSP	CULT	CERE	COMM	INRE	OUTRE	DIST	MOBI
OUTSP	1	0.95	0.37	0.71	0.52	0.79		0.86	0.75
INSP	0.95	1	0.41	0.69	0.52	0.75		0.70	0.62
CULT	0.37	0.41	1	0.34	0.68	0.35		0.34	0.10
CERE	0.71	0.69	0.34	1	0.63	0.92		0.60	0.65
COMM	0.52	0.52	0.68	0.63	1	0.60		0.49	0.36
INRE	0.79	0.75	0.35	0.92	0.60	1		0.69	0.70
OUTRE									
DIST	0.86	0.70	0.34	0.60	0.49	0.69		1	0.75
MOBI	0.75	0.62	0.10	0.65	0.36	0.70		0.75	1

Supplementary Table 6: Crossed correlations among the 9 fields in Cantabria.

	OUTSP	INSP	CULT	CERE	COMM	INRE	OUTRE	DIST	MOBI
OUTSP	1	1		0.91	0.43	0.83	0.68	0.82	0.77
INSP	1	1		0.91	0.43	0.83	0.68	0.82	0.77
CULT									
CERE	0.91	0.91		1	0.65	0.53	0.33	0.89	0.68
COMM	0.43	0.43		0.65	1	0.06	-0.11	0.57	0.31
INRE	0.83	0.83		0.53	0.06	1	0.97	0.48	0.67
OUTRE	0.68	0.68		0.33	-0.11	0.97	1	0.26	0.52
DIST	0.82	0.82		0.89	0.57	0.48	0.26	1	0.84
MOBI	0.77	0.77		0.68	0.31	0.67	0.52	0.84	1

Supplementary Table 7: Crossed correlations among the 9 fields in Bizkaia.

	OUTSP	INSP	CULT	CERE	COMM	INRE	OUTRE	DIST	MOBI
OUTSP	1	1		0.91	0.45	0.84	0.70	0.81	0.75
INSP	1	1		0.91	0.45	0.84	0.70	0.81	0.75
CULT									
CERE	0.91	0.91		1	0.65	0.56	0.37	0.89	0.68
COMM	0.45	0.45		0.65	1	0.12	-0.05	0.57	0.31
INRE	0.84	0.84		0.56	0.12	1	0.97	0.51	0.66
OUTRE	0.70	0.70		0.37	-0.05	0.97	1	0.29	0.51
DIST	0.81	0.81		0.89	0.57	0.51	0.29	1	0.84
MOBI	0.75	0.75		0.68	0.31	0.66	0.51	0.84	1

Supplementary Table 8: Crossed correlations among the 9 fields in Guipuzkoa.

	OUTSP	INSP	CULT	CERE	COMM	INRE	OUTRE	DIST	MOBI
OUTSP	1	1		0.90	0.46	0.83	0.69	0.80	0.73
INSP	1	1		0.90	0.46	0.83	0.69	0.80	0.73
CULT									
CERE	0.90	0.90		1	0.65	0.54	0.35	0.89	0.68
COMM	0.46	0.46		0.65	1	0.15	-0.01	0.57	0.31
INRE	0.83	0.83		0.54	0.15	1	0.97	0.48	0.62
OUTRE	0.69	0.69		0.35	-0.01	0.97	1	0.28	0.47
DIST	0.80	0.80		0.89	0.57	0.48	0.28	1	0.84
MOBI	0.73	0.73		0.68	0.31	0.62	0.47	0.84	1

Supplementary Table 9: Crossed correlations among the 9 fields in Álava.

	OUTSP	INSP	CULT	CERE	COMM	INRE	OUTRE	DIST	MOBI
OUTSP	1	0.97	0.95	0.38	-0.17	0.42	0.65	0.22	0.08
INSP	0.97	1	0.97	0.27	-0.21	0.32	0.71	0.07	-0.09
CULT	0.95	0.97	1	0.33	-0.09	0.40	0.77	0.18	0.02
CERE	0.38	0.27	0.33	1	0.65	0.74	0.23	0.72	0.62
COMM	-0.17	-0.21	-0.09	0.65	1	0.62	0.08	0.56	0.52
INRE	0.42	0.32	0.40	0.74	0.62	1	0.43	0.65	0.59
OUTRE	0.65	0.71	0.77	0.23	0.08	0.43	1	0.27	0.17
DIST	0.22	0.07	0.18	0.72	0.56	0.65	0.27	1	0.94
MOBI	0.08	-0.09	0.02	0.62	0.52	0.59	0.17	0.94	1

Supplementary Table 10: Crossed correlations among the 9 fields in Navarra.

	OUTSP	INSP	CULT	CERE	COMM	INRE	OUTRE	DIST	MOBI
OUTSP	1	0.97	0.95	0.38	-0.17	0.42	0.65	0.22	0.08
INSP	0.97	1	0.97	0.27	-0.21	0.32	0.71	0.07	-0.09
CULT	0.95	0.97	1	0.33	-0.09	0.40	0.77	0.18	0.02
CERE	0.38	0.27	0.33	1	0.65	0.74	0.23	0.72	0.62
COMM	-0.17	-0.21	-0.09	0.65	1	0.62	0.08	0.56	0.52
INRE	0.42	0.32	0.40	0.74	0.62	1	0.43	0.65	0.59
OUTRE	0.65	0.71	0.77	0.23	0.08	0.43	1	0.27	0.17
DIST	0.22	0.07	0.18	0.72	0.56	0.65	0.27	1	0.94
MOBI	0.08	-0.09	0.02	0.62	0.52	0.59	0.17	0.94	1

Supplementary Table 11: Crossed correlations among the 9 fields in La Rioja.

	OUTSP	INSP	CULT	CERE	COMM	INRE	OUTRE	DIST	MOBI
OUTSP	1	0.94	0.94	0.85	0.91	0.75	0.56	0.95	0.73
INSP	0.94	1	0.94	0.82	0.84	0.87	0.80	0.88	0.64
CULT	0.94	0.94	1	0.90	0.89	0.88	0.67	0.93	0.63
CERE	0.85	0.82	0.90	1	0.76	0.72	0.54	0.82	0.51
COMM	0.91	0.84	0.89	0.76	1	0.78	0.48	0.97	0.87
INRE	0.75	0.87	0.88	0.72	0.78	1	0.80	0.74	0.48
OUTRE	0.56	0.80	0.67	0.54	0.48	0.80	1	0.52	0.30
DIST	0.95	0.88	0.93	0.82	0.97	0.74	0.52	1	0.86
MOBI	0.73	0.64	0.63	0.51	0.87	0.48	0.30	0.86	1

Supplementary Table 12: Crossed correlations among the 9 fields in Huesca.

	OUTSP	INSP	CULT	CERE	COMM	INRE	OUTRE	DIST	MOBI
OUTSP	1	0.94	0.93	0.84	0.90	0.77	0.58	0.93	0.71
INSP	0.94	1	0.93	0.81	0.82	0.87	0.82	0.86	0.62
CULT	0.93	0.93	1	0.90	0.88	0.88	0.68	0.93	0.62
CERE	0.84	0.81	0.90	1	0.76	0.73	0.55	0.82	0.51
COMM	0.90	0.82	0.88	0.76	1	0.78	0.48	0.97	0.86
INRE	0.77	0.87	0.88	0.73	0.78	1	0.80	0.74	0.47
OUTRE	0.58	0.82	0.68	0.55	0.48	0.80	1	0.52	0.30
DIST	0.93	0.86	0.93	0.82	0.97	0.74	0.52	1	0.85
MOBI	0.71	0.62	0.62	0.51	0.86	0.47	0.30	0.85	1

Supplementary Table 13: Crossed correlations among the 9 fields in Zaragoza.

	OUTSP	INSP	CULT	CERE	COMM	INRE	OUTRE	DIST	MOBI
OUTSP	1	0.95	0.93	0.84	0.90	0.82	0.59	0.93	0.71
INSP	0.95	1	0.94	0.83	0.83	0.89	0.80	0.88	0.62
CULT	0.93	0.94	1	0.90	0.89	0.92	0.69	0.93	0.62
CERE	0.84	0.83	0.90	1	0.77	0.79	0.56	0.83	0.51
COMM	0.90	0.83	0.89	0.77	1	0.82	0.48	0.97	0.87
INRE	0.82	0.89	0.92	0.79	0.82	1	0.78	0.80	0.51
OUTRE	0.59	0.80	0.69	0.56	0.48	0.78	1	0.54	0.31
DIST	0.93	0.88	0.93	0.83	0.97	0.80	0.54	1	0.85
MOBI	0.71	0.62	0.62	0.51	0.87	0.51	0.31	0.85	1

Supplementary Table 14: Crossed correlations among the 9 fields in Teruel.

	OUTSP	INSP	CULT	CERE	COMM	INRE	OUTRE	DIST	MOBI
OUTSP	1	0.85	0.99	0.86	0.73	0.75	0.32	0.30	0.48
INSP	0.85	1	0.88	0.73	0.80	0.95	0.35	0.33	0.49
CULT	0.99	0.88	1	0.84	0.75	0.78	0.36	0.27	0.42
CERE	0.86	0.73	0.84	1	0.89	0.54	-0.17	-0.01	0.39
COMM	0.73	0.80	0.75	0.89	1	0.65	-0.24	-0.06	0.31
INRE	0.75	0.95	0.78	0.54	0.65	1	0.49	0.45	0.54
OUTRE	0.32	0.35	0.36	-0.17	-0.24	0.49	1	0.58	0.20
DIST	0.30	0.33	0.27	-0.01	-0.06	0.45	0.58	1	0.65
MOBI	0.48	0.49	0.42	0.39	0.31	0.54	0.20	0.65	1

Supplementary Table 15: Crossed correlations among the 9 fields in León.

	OUTSP	INSP	CULT	CERE	COMM	INRE	OUTRE	DIST	MOBI
OUTSP	1	0.85	0.75	0.69	0.60	0.78	0.29	0.22	0.50
INSP	0.85	1	0.64	0.63	0.76	0.94	0.40	0.32	0.49
CULT	0.75	0.64	1	0.29	0.34	0.59	0.22	0.12	0.33
CERE	0.69	0.63	0.29	1	0.87	0.47	-0.08	-0.06	0.37
COMM	0.60	0.76	0.34	0.87	1	0.60	-0.11	-0.03	0.32
INRE	0.78	0.94	0.59	0.47	0.60	1	0.52	0.46	0.56
OUTRE	0.29	0.40	0.22	-0.08	-0.11	0.52	1	0.53	0.25
DIST	0.22	0.32	0.12	-0.06	-0.03	0.46	0.53	1	0.68
MOBI	0.50	0.49	0.33	0.37	0.32	0.56	0.25	0.68	1

Supplementary Table 16: Crossed correlations among the 9 fields in Palencia.

	OUTSP	INSP	CULT	CERE	COMM	INRE	OUTRE	DIST	MOBI
OUTSP	1	0.84	0.78	0.75	0.65	0.73	0.50	0.41	0.57
INSP	0.84	1	0.66	0.70	0.83	0.89	0.36	0.34	0.55
CULT	0.78	0.66	1	0.22	0.36	0.53	0.77	0.38	0.37
CERE	0.75	0.70	0.22	1	0.76	0.60	-0.09	0.14	0.49
COMM	0.65	0.83	0.36	0.76	1	0.64	-0.14	0	0.39
INRE	0.73	0.89	0.53	0.60	0.64	1	0.40	0.44	0.61
OUTRE	0.50	0.36	0.77	-0.09	-0.14	0.40	1	0.54	0.22
DIST	0.41	0.34	0.38	0.14	0	0.44	0.54	1	0.66
MOBI	0.57	0.55	0.37	0.49	0.39	0.61	0.22	0.66	1

Supplementary Table 17: Crossed correlations among the 9 fields in Burgos.

	OUTSP	INSP	CULT	CERE	COMM	INRE	OUTRE	DIST	MOBI
OUTSP	1	0.84	0.77	0.69	0.58	0.79	0.23	0.29	0.54
INSP	0.84	1	0.56	0.66	0.74	0.96	0.31	0.34	0.55
CULT	0.77	0.56	1	0.20	0.19	0.53	0.06	0.14	0.31
CERE	0.69	0.66	0.20	1	0.89	0.54	-0.17	0.01	0.44
COMM	0.58	0.74	0.19	0.89	1	0.60	-0.25	-0.04	0.33
INRE	0.79	0.96	0.53	0.54	0.60	1	0.43	0.45	0.61
OUTRE	0.23	0.31	0.06	-0.17	-0.25	0.43	1	0.56	0.20
DIST	0.29	0.34	0.14	0.01	-0.04	0.45	0.56	1	0.67
MOBI	0.54	0.55	0.31	0.44	0.33	0.61	0.20	0.67	1

Supplementary Table 18: Crossed correlations among the 9 fields in Soria.

	OUTSP	INSP	CULT	CERE	COMM	INRE	OUTRE	DIST	MOBI
OUTSP	1	0.84	0.77	0.69	0.58	0.72	0.23	0.29	0.54
INSP	0.84	1	0.56	0.66	0.74	0.96	0.31	0.34	0.55
CULT	0.77	0.56	1	0.20	0.19	0.52	0.06	0.14	0.31
CERE	0.69	0.66	0.20	1	0.89	0.44	-0.17	0.01	0.44
COMM	0.58	0.74	0.19	0.89	1	0.57	-0.25	-0.04	0.33
INRE	0.72	0.96	0.52	0.44	0.57	1	0.46	0.45	0.56
OUTRE	0.23	0.31	0.06	-0.17	-0.25	0.46	1	0.56	0.20
DIST	0.29	0.34	0.14	0.01	-0.04	0.45	0.56	1	0.67
MOBI	0.54	0.55	0.31	0.44	0.33	0.56	0.20	0.67	1

Supplementary Table 19: Crossed correlations among the 9 fields in Zamora.

	OUTSP	INSP	CULT	CERE	COMM	INRE	OUTRE	DIST	MOBI
OUTSP	1	0.83	0.77	0.68	0.58	0.79	0.23	0.26	0.52
INSP	0.83	1	0.56	0.65	0.74	0.94	0.30	0.32	0.54
CULT	0.77	0.56	1	0.20	0.19	0.53	0.06	0.14	0.31
CERE	0.68	0.65	0.20	1	0.89	0.54	-0.18	-0.02	0.41
COMM	0.58	0.74	0.19	0.89	1	0.60	-0.25	-0.04	0.33
INRE	0.79	0.94	0.53	0.54	0.60	1	0.42	0.44	0.61
OUTRE	0.23	0.30	0.06	-0.18	-0.25	0.42	1	0.56	0.20
DIST	0.26	0.32	0.14	-0.02	-0.04	0.44	0.56	1	0.67
MOBI	0.52	0.54	0.31	0.41	0.33	0.61	0.20	0.67	1

Supplementary Table 20: Crossed correlations among the 9 fields in Valladolid.

	OUTSP	INSP	CULT	CERE	COMM	INRE	OUTRE	DIST	MOBI
OUTSP	1	0.81	0.95	0.83	0.67	0.74	0.30	0.27	0.46
INSP	0.81	1	0.88	0.66	0.76	0.94	0.35	0.34	0.52
CULT	0.95	0.88	1	0.79	0.73	0.80	0.31	0.29	0.47
CERE	0.83	0.66	0.79	1	0.83	0.51	-0.23	-0.11	0.34
COMM	0.67	0.76	0.73	0.83	1	0.59	-0.30	-0.03	0.33
INRE	0.74	0.94	0.80	0.51	0.59	1	0.49	0.46	0.57
OUTRE	0.30	0.35	0.31	-0.23	-0.30	0.49	1	0.57	0.21
DIST	0.27	0.34	0.29	-0.11	-0.03	0.46	0.57	1	0.69
MOBI	0.46	0.52	0.47	0.34	0.33	0.57	0.21	0.69	1

Supplementary Table 21: Crossed correlations among the 9 fields in Salamanca.

	OUTSP	INSP	CULT	CERE	COMM	INRE	OUTRE	DIST	MOBI
OUTSP	1	0.82	0.99	0.84	0.55	0.70	0.25	0.34	0.54
INSP	0.82	1	0.86	0.61	0.79	0.97	0.44	0.45	0.52
CULT	0.99	0.86	1	0.81	0.60	0.73	0.30	0.32	0.47
CERE	0.84	0.61	0.81	1	0.23	0.46	-0.23	0.01	0.45
COMM	0.55	0.79	0.60	0.23	1	0.75	0.69	0.51	0.38
INRE	0.70	0.97	0.73	0.46	0.75	1	0.50	0.49	0.52
OUTRE	0.25	0.44	0.30	-0.23	0.69	0.50	1	0.59	0.21
DIST	0.34	0.45	0.32	0.01	0.51	0.49	0.59	1	0.67
MOBI	0.54	0.52	0.47	0.45	0.38	0.52	0.21	0.67	1

Supplementary Table 22: Crossed correlations among the 9 fields in Ávila.

	OUTSP	INSP	CULT	CERE	COMM	INRE	OUTRE	DIST	MOBI
OUTSP	1	0.84	0.79	0.80	0.55	0.76	0.29	0.33	0.56
INSP	0.84	1	0.77	0.58	0.75	0.95	0.45	0.42	0.52
CULT	0.79	0.77	1	0.68	0.51	0.75	0.30	0.26	0.41
CERE	0.80	0.58	0.68	1	0.20	0.54	-0.23	0.02	0.47
COMM	0.55	0.75	0.51	0.20	1	0.73	0.68	0.49	0.39
INRE	0.76	0.95	0.75	0.54	0.73	1	0.49	0.47	0.57
OUTRE	0.29	0.45	0.30	-0.23	0.68	0.49	1	0.57	0.22
DIST	0.33	0.42	0.26	0.02	0.49	0.47	0.57	1	0.67
MOBI	0.56	0.52	0.41	0.47	0.39	0.57	0.22	0.67	1

Supplementary Table 23: Crossed correlations among the 9 fields in Segovia.

	OUTSP	INSP	CULT	CERE	COMM	INRE	OUTRE	DIST	MOBI
OUTSP	1	-0.28		0.87	0.07	-0.05	0.22	0.39	-0.15
INSP	-0.28	1		-0.58	0.81	0.76	0.70	-0.01	0.95
CULT									
CERE	0.87	-0.58		1	-0.09	-0.21	-0.01	0.51	-0.45
COMM	0.07	0.81		-0.09	1	0.85	0.85	0.26	0.82
INRE	-0.05	0.76		-0.21	0.85	1	0.77	0.13	0.70
OUTRE	0.22	0.70		-0.01	0.85	0.77	1	0.21	0.70
DIST	0.39	-0.01		0.51	0.26	0.13	0.21	1	0.05
MOBI	-0.15	0.95		-0.45	0.82	0.70	0.70	0.05	1

Supplementary Table 24: Crossed correlations among the 9 fields in Madrid.

	OUTSP	INSP	CULT	CERE	COMM	INRE	OUTRE	DIST	MOBI
OUTSP	1	0.87	0.55	0.88	0.58	0.12	0.03	0.63	-0.10
INSP	0.87	1	0.68	0.80	0.53	0.10	-0.07	0.72	-0.04
CULT	0.55	0.68	1	0.70	0.73	0.67	0.45	0.88	0.36
CERE	0.88	0.80	0.70	1	0.84	0.40	0.30	0.75	0.01
COMM	0.58	0.53	0.73	0.84	1	0.77	0.71	0.68	0.31
INRE	0.12	0.10	0.67	0.40	0.77	1	0.91	0.55	0.64
OUTRE	0.03	-0.07	0.45	0.30	0.71	0.91	1	0.43	0.39
DIST	0.63	0.72	0.88	0.75	0.68	0.55	0.43	1	0.13
MOBI	-0.10	-0.04	0.36	0.01	0.31	0.64	0.39	0.13	1

Supplementary Table 25: Crossed correlations among the 9 fields in Guadalajara.

	OUTSP	INSP	CULT	CERE	COMM	INRE	OUTRE	DIST	MOBI
OUTSP	1	0.87	0.82	0.89	0.68	0.39	0.12	0.78	0.25
INSP	0.87	1	0.70	0.75	0.60	0.30	0.02	0.61	0.36
CULT	0.82	0.70	1	0.84	0.81	0.74	0.52	0.90	0.34
CERE	0.89	0.75	0.84	1	0.83	0.50	0.32	0.88	0.07
COMM	0.68	0.60	0.81	0.83	1	0.85	0.73	0.72	0.43
INRE	0.39	0.30	0.74	0.50	0.85	1	0.95	0.61	0.57
OUTRE	0.12	0.02	0.52	0.32	0.73	0.95	1	0.45	0.43
DIST	0.78	0.61	0.90	0.88	0.72	0.61	0.45	1	0.08
MOBI	0.25	0.36	0.34	0.07	0.43	0.57	0.43	0.08	1

Supplementary Table 26: Crossed correlations among the 9 fields in Cuenca.

	OUTSP	INSP	CULT	CERE	COMM	INRE	OUTRE	DIST	MOBI
OUTSP	1	0.84	0.69	0.92	0.63	0.38	0.07	0.83	-0.08
INSP	0.84	1	0.62	0.80	0.56	0.28	-0.05	0.68	0
CULT	0.69	0.62	1	0.77	0.81	0.79	0.50	0.87	0.43
CERE	0.92	0.80	0.77	1	0.84	0.57	0.31	0.88	-0.02
COMM	0.63	0.56	0.81	0.84	1	0.86	0.69	0.74	0.36
INRE	0.38	0.28	0.79	0.57	0.86	1	0.87	0.70	0.50
OUTRE	0.07	-0.05	0.50	0.31	0.69	0.87	1	0.45	0.37
DIST	0.83	0.68	0.87	0.88	0.74	0.70	0.45	1	0.05
MOBI	-0.08	0	0.43	-0.02	0.36	0.50	0.37	0.05	1

Supplementary Table 27: Crossed correlations among the 9 fields in Toledo.

	OUTSP	INSP	CULT	CERE	COMM	INRE	OUTRE	DIST	MOBI
OUTSP	1	0.86	0.76	0.93	0.56	0.52	0.16	0.82	0.08
INSP	0.86	1	0.68	0.81	0.52	0.45	0.06	0.68	0.16
CULT	0.76	0.68	1	0.80	0.64	0.85	0.54	0.87	0.44
CERE	0.93	0.81	0.80	1	0.68	0.64	0.37	0.87	0.07
COMM	0.56	0.52	0.64	0.68	1	0.78	0.73	0.57	0.44
INRE	0.52	0.45	0.85	0.64	0.78	1	0.83	0.69	0.62
OUTRE	0.16	0.06	0.54	0.37	0.73	0.83	1	0.48	0.46
DIST	0.82	0.68	0.87	0.87	0.57	0.69	0.48	1	0.10
MOBI	0.08	0.16	0.44	0.07	0.44	0.62	0.46	0.10	1

Supplementary Table 28: Crossed correlations among the 9 fields in Ciudad Real.

	OUTSP	INSP	CULT	CERE	COMM	INRE	OUTRE	DIST	MOBI
OUTSP	1	0.87	0.83	0.89	0.69	0.39	0.12	0.77	0.26
INSP	0.87	1	0.73	0.74	0.61	0.32	0.03	0.60	0.37
CULT	0.83	0.73	1	0.82	0.82	0.75	0.52	0.87	0.41
CERE	0.89	0.74	0.82	1	0.82	0.49	0.31	0.88	0.05
COMM	0.69	0.61	0.82	0.82	1	0.85	0.73	0.71	0.44
INRE	0.39	0.32	0.75	0.49	0.85	1	0.94	0.60	0.58
OUTRE	0.12	0.03	0.52	0.31	0.73	0.94	1	0.44	0.45
DIST	0.77	0.60	0.87	0.88	0.71	0.60	0.44	1	0.05
MOBI	0.26	0.37	0.41	0.05	0.44	0.58	0.45	0.05	1

Supplementary Table 29: Crossed correlations among the 9 fields in Albacete.

	OUTSP	INSP	CULT	CERE	COMM	INRE	OUTRE	DIST	MOBI
OUTSP	1	0.95	0.50	0.83	0.83	0.88	0.84	0.64	0.59
INSP	0.95	1	0.47	0.80	0.70	0.89	0.72	0.71	0.65
CULT	0.50	0.47	1	0.36	0.34	0.69	0.46	0.57	0.64
CERE	0.83	0.80	0.36	1	0.59	0.58	0.44	0.73	0.68
COMM	0.83	0.70	0.34	0.59	1	0.74	0.90	0.39	0.38
INRE	0.88	0.89	0.69	0.58	0.74	1	0.82	0.66	0.66
OUTRE	0.84	0.72	0.46	0.44	0.90	0.82	1	0.31	0.29
DIST	0.64	0.71	0.57	0.73	0.39	0.66	0.31	1	0.96
MOBI	0.59	0.65	0.64	0.68	0.38	0.66	0.29	0.96	1

Supplementary Table 30: Crossed correlations among the 9 fields in Castellón.

	OUTSP	INSP	CULT	CERE	COMM	INRE	OUTRE	DIST	MOBI
OUTSP	1	0.95	0.51	0.83	0.84	0.88	0.84	0.64	0.59
INSP	0.95	1	0.49	0.80	0.71	0.89	0.71	0.71	0.65
CULT	0.51	0.49	1	0.39	0.35	0.69	0.46	0.54	0.62
CERE	0.83	0.80	0.39	1	0.60	0.58	0.44	0.72	0.67
COMM	0.84	0.71	0.35	0.60	1	0.75	0.90	0.38	0.37
INRE	0.88	0.89	0.69	0.58	0.75	1	0.82	0.66	0.66
OUTRE	0.84	0.71	0.46	0.44	0.90	0.82	1	0.31	0.29
DIST	0.64	0.71	0.54	0.72	0.38	0.66	0.31	1	0.96
MOBI	0.59	0.65	0.62	0.67	0.37	0.66	0.29	0.96	1

Supplementary Table 31: Crossed correlations among the 9 fields in Valencia.

	OUTSP	INSP	CULT	CERE	COMM	INRE	OUTRE	DIST	MOBI
OUTSP	1	0.95	0.50	0.82	0.84	0.88	0.84	0.64	0.58
INSP	0.95	1	0.48	0.80	0.71	0.89	0.72	0.71	0.65
CULT	0.50	0.48	1	0.39	0.35	0.69	0.46	0.54	0.62
CERE	0.82	0.80	0.39	1	0.59	0.58	0.44	0.72	0.67
COMM	0.84	0.71	0.35	0.59	1	0.74	0.90	0.37	0.36
INRE	0.88	0.89	0.69	0.58	0.74	1	0.82	0.66	0.65
OUTRE	0.84	0.72	0.46	0.44	0.90	0.82	1	0.31	0.29
DIST	0.64	0.71	0.54	0.72	0.37	0.66	0.31	1	0.96
MOBI	0.58	0.65	0.62	0.67	0.36	0.65	0.29	0.96	1

Supplementary Table 32: Crossed correlations among the 9 fields in Alicante.

	OUTSP	INSP	CULT	CERE	COMM	INRE	OUTRE	DIST	MOBI
OUTSP	1	0.89	-0.45	0.69	0.42	0.56	0.27	0.42	0.50
INSP	0.89	1	-0.50	0.80	0.48	0.81	0.53	0.32	0.67
CULT	-0.45	-0.50	1	-0.31	-0.95	-0.54	-0.34	-0.49	-0.80
CERE	0.69	0.80	-0.31	1	0.29	0.80	0.60	0.31	0.61
COMM	0.42	0.48	-0.95	0.29	1	0.51	0.32	0.50	0.76
INRE	0.56	0.81	-0.54	0.80	0.51	1	0.83	0.40	0.83
OUTRE	0.27	0.53	-0.34	0.60	0.32	0.83	1	0.21	0.63
DIST	0.42	0.32	-0.49	0.31	0.50	0.40	0.21	1	0.59
MOBI	0.50	0.67	-0.80	0.61	0.76	0.83	0.63	0.59	1

Supplementary Table 33: Crossed correlations among the 9 fields in Murcia.

	OUTSP	INSP	CULT	CERE	COMM	INRE	OUTRE	DIST	MOBI
OUTSP	1	0.97	0.68	0.92	0.55	0.79	0.74	0.75	0.48
INSP	0.97	1	0.83	0.89	0.72	0.84	0.86	0.73	0.52
CULT	0.68	0.83	1	0.62	0.96	0.76	0.94	0.51	0.50
CERE	0.92	0.89	0.62	1	0.56	0.91	0.74	0.90	0.68
COMM	0.55	0.72	0.96	0.56	1	0.77	0.92	0.51	0.57
INRE	0.79	0.84	0.76	0.91	0.77	1	0.89	0.89	0.80
OUTRE	0.74	0.86	0.94	0.74	0.92	0.89	1	0.61	0.56
DIST	0.75	0.73	0.51	0.90	0.51	0.89	0.61	1	0.86
MOBI	0.48	0.52	0.50	0.68	0.57	0.80	0.56	0.86	1

Supplementary Table 34: Crossed correlations among the 9 fields in Huelva.

	OUTSP	INSP	CULT	CERE	COMM	INRE	OUTRE	DIST	MOBI
OUTSP	1	0.99	0.83	0.95	0.62	0.69	0.78	0.68	0.45
INSP	0.99	1	0.89	0.95	0.70	0.71	0.82	0.68	0.48
CULT	0.83	0.89	1	0.80	0.87	0.67	0.86	0.58	0.52
CERE	0.95	0.95	0.80	1	0.70	0.84	0.80	0.87	0.66
COMM	0.62	0.70	0.87	0.70	1	0.76	0.81	0.70	0.76
INRE	0.69	0.71	0.67	0.84	0.76	1	0.89	0.91	0.84
OUTRE	0.78	0.82	0.86	0.80	0.81	0.89	1	0.69	0.62
DIST	0.68	0.68	0.58	0.87	0.70	0.91	0.69	1	0.90
MOBI	0.45	0.48	0.52	0.66	0.76	0.84	0.62	0.90	1

Supplementary Table 35: Crossed correlations among the 9 fields in Sevilla.

	OUTSP	INSP	CULT	CERE	COMM	INRE	OUTRE	DIST	MOBI
OUTSP	1	0.95	0.62	0.94	0.57	0.83	0.73	0.69	0.60
INSP	0.95	1	0.84	0.88	0.79	0.89	0.89	0.65	0.64
CULT	0.62	0.84	1	0.56	0.98	0.77	0.94	0.41	0.56
CERE	0.94	0.88	0.56	1	0.54	0.89	0.69	0.85	0.75
COMM	0.57	0.79	0.98	0.54	1	0.78	0.94	0.44	0.60
INRE	0.83	0.89	0.77	0.89	0.78	1	0.89	0.81	0.82
OUTRE	0.73	0.89	0.94	0.69	0.94	0.89	1	0.51	0.62
DIST	0.69	0.65	0.41	0.85	0.44	0.81	0.51	1	0.87
MOBI	0.60	0.64	0.56	0.75	0.60	0.82	0.62	0.87	1

Supplementary Table 36: Crossed correlations among the 9 fields in Cádiz.

	OUTSP	INSP	CULT	CERE	COMM	INRE	OUTRE	DIST	MOBI
OUTSP	1	0.99	0.80	0.94	0.64	0.88	0.85	0.79	0.58
INSP	0.99	1	0.87	0.93	0.73	0.90	0.91	0.78	0.61
CULT	0.80	0.87	1	0.74	0.91	0.81	0.94	0.62	0.59
CERE	0.94	0.93	0.74	1	0.66	0.95	0.82	0.92	0.74
COMM	0.64	0.73	0.91	0.66	1	0.79	0.88	0.63	0.69
INRE	0.88	0.90	0.81	0.95	0.79	1	0.90	0.92	0.82
OUTRE	0.85	0.91	0.94	0.82	0.88	0.90	1	0.67	0.61
DIST	0.79	0.78	0.62	0.92	0.63	0.92	0.67	1	0.90
MOBI	0.58	0.61	0.59	0.74	0.69	0.82	0.61	0.90	1

Supplementary Table 37: Crossed correlations among the 9 fields in Córdoba.

	OUTSP	INSP	CULT	CERE	COMM	INRE	OUTRE	DIST	MOBI
OUTSP	1	0.94	0.62	0.94	0.51	0.78	0.70	0.73	0.56
INSP	0.94	1	0.85	0.88	0.76	0.84	0.87	0.68	0.60
CULT	0.62	0.85	1	0.56	0.97	0.73	0.93	0.42	0.52
CERE	0.94	0.88	0.56	1	0.50	0.86	0.67	0.89	0.73
COMM	0.51	0.76	0.97	0.50	1	0.76	0.94	0.44	0.57
INRE	0.78	0.84	0.73	0.86	0.76	1	0.88	0.86	0.83
OUTRE	0.70	0.87	0.93	0.67	0.94	0.88	1	0.55	0.60
DIST	0.73	0.68	0.42	0.89	0.44	0.86	0.55	1	0.89
MOBI	0.56	0.60	0.52	0.73	0.57	0.83	0.60	0.89	1

Supplementary Table 38: Crossed correlations among the 9 fields in Málaga.

	OUTSP	INSP	CULT	CERE	COMM	INRE	OUTRE	DIST	MOBI
OUTSP	1	0.99	0.78	0.96	0.71	0.85	0.85	0.75	0.56
INSP	0.99	1	0.87	0.95	0.81	0.86	0.91	0.73	0.58
CULT	0.78	0.87	1	0.74	0.96	0.77	0.93	0.56	0.53
CERE	0.96	0.95	0.74	1	0.71	0.92	0.82	0.90	0.74
COMM	0.71	0.81	0.96	0.71	1	0.80	0.91	0.61	0.62
INRE	0.85	0.86	0.77	0.92	0.80	1	0.90	0.87	0.79
OUTRE	0.85	0.91	0.93	0.82	0.91	0.90	1	0.64	0.57
DIST	0.75	0.73	0.56	0.90	0.61	0.87	0.64	1	0.90
MOBI	0.56	0.58	0.53	0.74	0.62	0.79	0.57	0.90	1

Supplementary Table 39: Crossed correlations among the 9 fields in Jaén.

	OUTSP	INSP	CULT	CERE	COMM	INRE	OUTRE	DIST	MOBI
OUTSP	1	0.85	0.52	0.96	0.43	0.69	0.54	0.73	0.53
INSP	0.85	1	0.89	0.81	0.83	0.88	0.89	0.62	0.44
CULT	0.52	0.89	1	0.50	0.99	0.84	0.99	0.39	0.26
CERE	0.96	0.81	0.50	1	0.42	0.78	0.54	0.90	0.72
COMM	0.43	0.83	0.99	0.42	1	0.82	0.98	0.35	0.26
INRE	0.69	0.88	0.84	0.78	0.82	1	0.89	0.79	0.68
OUTRE	0.54	0.89	0.99	0.54	0.98	0.89	1	0.46	0.35
DIST	0.73	0.62	0.39	0.90	0.35	0.79	0.46	1	0.87
MOBI	0.53	0.44	0.26	0.72	0.26	0.68	0.35	0.87	1

Supplementary Table 40: Crossed correlations among the 9 fields in Granada.

	OUTSP	INSP	CULT	CERE	COMM	INRE	OUTRE	DIST	MOBI
OUTSP	1	0.94	0.67	0.88	0.57	0.77	0.75	0.70	0.55
INSP	0.94	1	0.88	0.82	0.81	0.83	0.92	0.64	0.57
CULT	0.67	0.88	1	0.57	0.97	0.76	0.96	0.44	0.48
CERE	0.88	0.82	0.57	1	0.54	0.88	0.73	0.88	0.74
COMM	0.57	0.81	0.97	0.54	1	0.79	0.95	0.49	0.55
INRE	0.77	0.83	0.76	0.88	0.79	1	0.89	0.87	0.82
OUTRE	0.75	0.92	0.96	0.73	0.95	0.89	1	0.59	0.60
DIST	0.70	0.64	0.44	0.88	0.49	0.87	0.59	1	0.89
MOBI	0.55	0.57	0.48	0.74	0.55	0.82	0.60	0.89	1

Supplementary Table 41: Crossed correlations among the 9 fields in Almería.

	OUTSP	INSP	CULT	CERE	COMM	INRE	OUTRE	DIST	MOBI
OUTSP	1	0.79	0.68	0.62	0.48	0.49	0.59	0.24	0.26
INSP	0.79	1	0.78	0.71	0.80	0.76	0.72	0.39	0.58
CULT	0.68	0.78	1	0.53	0.86	0.90	0.91	0.29	0.55
CERE	0.62	0.71	0.53	1	0.54	0.42	0.32	0.22	0.19
COMM	0.48	0.80	0.86	0.54	1	0.96	0.84	0.38	0.65
INRE	0.49	0.76	0.90	0.42	0.96	1	0.93	0.34	0.67
OUTRE	0.59	0.72	0.91	0.32	0.84	0.93	1	0.18	0.54
DIST	0.24	0.39	0.29	0.22	0.38	0.34	0.18	1	0.75
MOBI	0.26	0.58	0.55	0.19	0.65	0.67	0.54	0.75	1

Supplementary Table 42: Crossed correlations among the 9 fields in Cáceres.

	OUTSP	INSP	CULT	CERE	COMM	INRE	OUTRE	DIST	MOBI
OUTSP	1	0.78	0.70	0.58	0.48	0.51	0.61	0.18	0.23
INSP	0.78	1	0.79	0.65	0.81	0.78	0.76	0.32	0.55
CULT	0.70	0.79	1	0.46	0.85	0.90	0.92	0.34	0.57
CERE	0.58	0.65	0.46	1	0.50	0.37	0.29	0.22	0.19
COMM	0.48	0.81	0.85	0.50	1	0.95	0.86	0.40	0.65
INRE	0.51	0.78	0.90	0.37	0.95	1	0.95	0.37	0.67
OUTRE	0.61	0.76	0.92	0.29	0.86	0.95	1	0.24	0.55
DIST	0.18	0.32	0.34	0.22	0.40	0.37	0.24	1	0.77
MOBI	0.23	0.55	0.57	0.19	0.65	0.67	0.55	0.77	1

Supplementary Table 43: Crossed correlations among the 9 fields in Badajoz.

	OUTSP	INSP	CULT	CERE	COMM	INRE	OUTRE	DIST	MOBI
OUTSP	1	0.85	0.51	0.71	0.83	0.50	0.93	0.62	0.48
INSP	0.85	1	0.47	0.37	0.77	0.65	0.98	0.71	0.36
CULT	0.51	0.47	1	0.59	0	0.80	0.50	0.74	0.73
CERE	0.71	0.37	0.59	1	0.43	0.44	0.49	0.64	0.83
COMM	0.83	0.77	0	0.43	1	0.20	0.81	0.38	0.17
INRE	0.50	0.65	0.80	0.44	0.20	1	0.65	0.74	0.67
OUTRE	0.93	0.98	0.50	0.49	0.81	0.65	1	0.68	0.41
DIST	0.62	0.71	0.74	0.64	0.38	0.74	0.68	1	0.85
MOBI	0.48	0.36	0.73	0.83	0.17	0.67	0.41	0.85	1

Supplementary Table 44: Crossed correlations among the 9 fields in Illes Balears.

	OUTSP	INSP	CULT	CERE	COMM	INRE	OUTRE	DIST	MOBI
OUTSP	1	0.99	0.97	0.81	0.97	0.85	0.97	0.82	0.88
INSP	0.99	1	0.97	0.80	0.96	0.86	0.95	0.79	0.86
CULT	0.97	0.97	1	0.89	0.97	0.87	0.95	0.79	0.88
CERE	0.81	0.80	0.89	1	0.84	0.78	0.80	0.74	0.81
COMM	0.97	0.96	0.97	0.84	1	0.92	0.94	0.81	0.92
INRE	0.85	0.86	0.87	0.78	0.92	1	0.81	0.59	0.74
OUTRE	0.97	0.95	0.95	0.80	0.94	0.81	1	0.86	0.90
DIST	0.82	0.79	0.79	0.74	0.81	0.59	0.86	1	0.93
MOBI	0.88	0.86	0.88	0.81	0.92	0.74	0.90	0.93	1

Supplementary Table 45: Crossed correlations among the 9 fields in Las Palmas.

	OUTSP	INSP	CULT	CERE	COMM	INRE	OUTRE	DIST	MOBI
OUTSP	1	0.84	0.91	0.70	0.54	0.86	0.92	0.80	0.90
INSP	0.84	1	0.79	0.90	0.48	0.74	0.90	0.74	0.66
CULT	0.91	0.79	1	0.67	0.28	0.92	0.81	0.69	0.80
CERE	0.70	0.90	0.67	1	0.65	0.71	0.79	0.49	0.53
COMM	0.54	0.48	0.28	0.65	1	0.39	0.61	0.39	0.57
INRE	0.86	0.74	0.92	0.71	0.39	1	0.74	0.53	0.74
OUTRE	0.92	0.90	0.81	0.79	0.61	0.74	1	0.83	0.83
DIST	0.80	0.74	0.69	0.49	0.39	0.53	0.83	1	0.87
MOBI	0.90	0.66	0.80	0.53	0.57	0.74	0.83	0.87	1

Supplementary Table 46: Crossed correlations among the 9 fields in Santa Cruz de Tenerife.

	OUTSP	INSP	CULT	CERE	COMM	INRE	OUTRE	DIST	MOBI
OUTSP	1	0.85	0.79	0.71	0.81	0.71	0.56	0.66	0.70
INSP	0.85	1	0.58	0.77	0.84	0.63	0.39	0.74	0.80
CULT	0.79	0.58	1	0.34	0.48	0.71	0.76	0.30	0.34
CERE	0.71	0.77	0.34	1	0.71	0.39	0.07	0.75	0.97
COMM	0.81	0.84	0.48	0.71	1	0.70	0.48	0.73	0.68
INRE	0.71	0.63	0.71	0.39	0.70	1	0.92	0.67	0.37
OUTRE	0.56	0.39	0.76	0.07	0.48	0.92	1	0.33	0.03
DIST	0.66	0.74	0.30	0.75	0.73	0.67	0.33	1	0.78
MOBI	0.70	0.80	0.34	0.97	0.68	0.37	0.03	0.78	1

Supplementary Table 47: Crossed correlations among the 9 fields in Barcelona.

	OUTSP	INSP	CULT	CERE	COMM	INRE	OUTRE	DIST	MOBI
OUTSP	1	0.92	0.70	0.83	0.85	0.78	0.53	0.73	0.80
INSP	0.92	1	0.54	0.86	0.86	0.71	0.39	0.76	0.85
CULT	0.70	0.54	1	0.36	0.49	0.71	0.76	0.28	0.35
CERE	0.83	0.86	0.36	1	0.74	0.50	0.13	0.73	0.97
COMM	0.85	0.86	0.49	0.74	1	0.72	0.48	0.65	0.68
INRE	0.78	0.71	0.71	0.50	0.72	1	0.89	0.63	0.41
OUTRE	0.53	0.39	0.76	0.13	0.48	0.89	1	0.30	0.03
DIST	0.73	0.76	0.28	0.73	0.65	0.63	0.30	1	0.70
MOBI	0.80	0.85	0.35	0.97	0.68	0.41	0.03	0.70	1

Supplementary Table 48: Crossed correlations among the 9 fields in Girona.

	OUTSP	INSP	CULT	CERE	COMM	INRE	OUTRE	DIST	MOBI
OUTSP	1	0.92	0.70	0.81	0.85	0.79	0.53	0.73	0.80
INSP	0.92	1	0.54	0.84	0.86	0.73	0.39	0.76	0.84
CULT	0.70	0.54	1	0.36	0.50	0.70	0.76	0.28	0.36
CERE	0.81	0.84	0.36	1	0.72	0.49	0.11	0.68	0.95
COMM	0.85	0.86	0.50	0.72	1	0.73	0.48	0.66	0.68
INRE	0.79	0.73	0.70	0.49	0.73	1	0.88	0.64	0.42
OUTRE	0.53	0.39	0.76	0.11	0.48	0.88	1	0.30	0.03
DIST	0.73	0.76	0.28	0.68	0.66	0.64	0.30	1	0.70
MOBI	0.80	0.84	0.36	0.95	0.68	0.42	0.03	0.70	1

Supplementary Table 49: Crossed correlations among the 9 fields in Lleida.

	OUTSP	INSP	CULT	CERE	COMM	INRE	OUTRE	DIST	MOBI
OUTSP	1	0.92	0.70	0.82	0.85	0.79	0.53	0.75	0.80
INSP	0.92	1	0.54	0.85	0.86	0.72	0.39	0.78	0.85
CULT	0.70	0.54	1	0.36	0.50	0.70	0.76	0.29	0.36
CERE	0.82	0.85	0.36	1	0.73	0.49	0.11	0.71	0.97
COMM	0.85	0.86	0.50	0.73	1	0.73	0.48	0.67	0.68
INRE	0.79	0.72	0.70	0.49	0.73	1	0.89	0.65	0.42
OUTRE	0.53	0.39	0.76	0.11	0.48	0.89	1	0.31	0.03
DIST	0.75	0.78	0.29	0.71	0.67	0.65	0.31	1	0.72
MOBI	0.80	0.85	0.36	0.97	0.68	0.42	0.03	0.72	1

Supplementary Table 50: Crossed correlations among the 9 fields in Tarragona.

**MEDIA**	OUTSP	INSP	CULT	CERE	COMM	INRE	OUTRE	DIST	MOBI
OUTSP	1	0.88	0.73	0.78	0.63	0.69	0.55	0.62	0.53
INSP	0.88	1	0.72	0.73	0.70	0.76	0.61	0.59	0.56
CULT	0.73	0.72	1	0.56	0.61	0.70	0.63	0.48	0.45
CERE	0.78	0.73	0.56	1	0.65	0.63	0.37	0.58	0.55
COMM	0.63	0.70	0.61	0.65	1	0.69	0.52	0.50	0.52
INRE	0.69	0.76	0.70	0.63	0.69	1	0.78	0.62	0.64
OUTRE	0.55	0.61	0.63	0.37	0.52	0.78	1	0.47	0.41
DIST	0.62	0.59	0.48	0.58	0.50	0.62	0.47	1	0.71
MOBI	0.53	0.56	0.45	0.55	0.52	0.64	0.41	0.71	1

Supplementary Table 51: Mean of the crossed correlations among the 9 fields in Spain.

**MEDIANA**	OUTSP	INSP	CULT	CERE	COMM	INRE	OUTRE	DIST	MOBI
OUTSP	1	0.92	0.77	0.83	0.63	0.77	0.59	0.71	0.56
INSP	0.92	1	0.79	0.80	0.76	0.84	0.71	0.69	0.59
CULT	0.77	0.79	1	0.58	0.74	0.75	0.76	0.44	0.47
CERE	0.83	0.80	0.58	1	0.68	0.58	0.35	0.72	0.65
COMM	0.63	0.76	0.74	0.68	1	0.74	0.69	0.56	0.52
INRE	0.77	0.84	0.75	0.58	0.74	1	0.86	0.65	0.64
OUTRE	0.59	0.71	0.76	0.35	0.69	0.86	1	0.52	0.39
DIST	0.71	0.69	0.44	0.72	0.56	0.65	0.52	1	0.80
MOBI	0.56	0.59	0.47	0.65	0.52	0.64	0.39	0.80	1

Supplementary Table 52: Median of the crossed correlations among the 9 fields in Spain.

$\boldsymbol{Q_1}$	OUTSP	INSP	CULT	CERE	COMM	INRE	OUTRE	DIST	MOBI
OUTSP	1	0.85	0.68	0.69	0.54	0.69	0.29	0.40	0.46
INSP	0.85	1	0.56	0.67	0.63	0.72	0.39	0.40	0.50
CULT	0.68	0.56	1	0.36	0.36	0.69	0.46	0.29	0.35
CERE	0.69	0.67	0.36	1	0.57	0.50	0.11	0.46	0.44
COMM	0.54	0.63	0.36	0.57	1	0.62	0.32	0.38	0.34
INRE	0.69	0.72	0.69	0.50	0.62	1	0.74	0.47	0.56
OUTRE	0.29	0.39	0.46	0.11	0.32	0.74	1	0.31	0.22
DIST	0.40	0.40	0.29	0.46	0.38	0.47	0.31	1	0.67
MOBI	0.46	0.50	0.35	0.44	0.34	0.56	0.22	0.67	1

Supplementary Table 53: First quartile of the crossed correlations among the 9 fields in Spain.

$\boldsymbol{Q_3}$	OUTSP	INSP	CULT	CERE	COMM	INRE	OUTRE	DIST	MOBI
OUTSP	1	0.95	0.83	0.89	0.84	0.82	0.73	0.81	0.73
INSP	0.95	1	0.89	0.83	0.83	0.89	0.82	0.74	0.71
CULT	0.83	0.89	1	0.80	0.89	0.82	0.86	0.63	0.62
CERE	0.89	0.83	0.80	1	0.76	0.79	0.67	0.87	0.72
COMM	0.84	0.83	0.89	0.76	1	0.79	0.85	0.69	0.68
INRE	0.82	0.89	0.82	0.79	0.79	1	0.90	0.72	0.74
OUTRE	0.73	0.82	0.86	0.67	0.85	0.90	1	0.56	0.60
DIST	0.81	0.74	0.63	0.87	0.69	0.72	0.56	1	0.87
MOBI	0.73	0.71	0.62	0.72	0.68	0.74	0.60	0.87	1

Supplementary Table 54: Third quartile of the crossed correlations among the 9 fields in Spain.

$\boldsymbol{q}_{0.1}$	OUTSP	INSP	CULT	CERE	COMM	INRE	OUTRE	DIST	MOBI
OUTSP	1	0.83	0.51	0.64	0.43	0.42	0.21	0.26	0.08
INSP	0.83	1	0.48	0.57	0.48	0.32	0.26	0.32	0.34
CULT	0.51	0.48	1	0.26	0.19	0.52	0.25	0.16	0.28
CERE	0.64	0.57	0.26	1	0.41	0.42	-0.17	0.01	0.07
COMM	0.43	0.48	0.19	0.41	1	0.50	-0.16	-0.03	0.31
INRE	0.42	0.32	0.52	0.42	0.50	1	0.45	0.44	0.47
OUTRE	0.21	0.26	0.25	-0.17	-0.16	0.45	1	0.27	0.17
DIST	0.26	0.32	0.16	0.01	-0.03	0.44	0.27	1	0.13
MOBI	0.08	0.34	0.28	0.07	0.31	0.47	0.17	0.13	1

Supplementary Table 55: Quantile 0.1 of the crossed correlations among the 9 fields in Spain.

$\boldsymbol{q}_{0.9}$	OUTSP	INSP	CULT	CERE	COMM	INRE	OUTRE	DIST	MOBI
OUTSP	1	0.99	0.95	0.94	0.89	0.86	0.85	0.87	0.82
INSP	0.99	1	0.94	0.90	0.87	0.95	0.91	0.81	0.84
CULT	0.95	0.94	1	0.84	0.97	0.88	0.94	0.87	0.67
CERE	0.94	0.90	0.84	1	0.84	0.91	0.82	0.89	0.84
COMM	0.89	0.87	0.97	0.84	1	0.85	0.93	0.76	0.77
INRE	0.86	0.95	0.88	0.91	0.85	1	0.95	0.86	0.83
OUTRE	0.85	0.91	0.94	0.82	0.93	0.95	1	0.65	0.70
DIST	0.87	0.81	0.87	0.89	0.76	0.86	0.65	1	0.93
MOBI	0.82	0.84	0.67	0.84	0.77	0.83	0.70	0.93	1

Supplementary Table 56: Quantile 0.9 of the crossed correlations among the 9 fields in Spain.

$q_{0.05}$	OUTSP	INSP	CULT	CERE	COMM	INRE	OUTRE	DIST	MOBI
OUTSP	1	0.80	0.50	0.47	0.23	0.38	0.12	0.22	-0.01
INSP	0.80	1	0.47	0.31	0.44	0.31	0.03	0.18	-0.02
CULT	0.50	0.47	1	0.20	-0.07	0.40	0.06	0.14	0.04
CERE	0.47	0.31	0.20	1	0.25	0.38	-0.21	-0.01	0.03
COMM	0.23	0.44	-0.07	0.25	1	0.17	-0.25	-0.04	0.31
INRE	0.38	0.31	0.40	0.38	0.17	1	0.43	0.39	0.42
OUTRE	0.12	0.03	0.06	-0.21	-0.25	0.43	1	0.22	0.03
DIST	0.22	0.18	0.14	-0.01	-0.04	0.39	0.22	1	0.07
MOBI	-0.01	-0.02	0.04	0.03	0.31	0.42	0.03	0.07	1

Supplementary Table 57: Quantile 0.05 of the crossed correlations among the 9 fields in Spain.

$q_{0.95}$	OUTSP	INSP	CULT	CERE	COMM	INRE	OUTRE	DIST	MOBI
OUTSP	1	1	0.97	0.95	0.90	0.88	0.93	0.91	0.88
INSP	1	1	0.96	0.92	0.91	0.96	0.94	0.84	0.85
CULT	0.97	0.96	1	0.90	0.97	0.90	0.95	0.92	0.73
CERE	0.95	0.92	0.90	1	0.89	0.92	0.87	0.90	0.97
COMM	0.90	0.91	0.97	0.89	1	0.90	0.94	0.90	0.87
INRE	0.88	0.96	0.90	0.92	0.90	1	0.96	0.88	0.84
OUTRE	0.93	0.94	0.95	0.87	0.94	0.96	1	0.69	0.76
DIST	0.91	0.84	0.92	0.90	0.90	0.88	0.69	1	0.95
MOBI	0.88	0.85	0.73	0.97	0.87	0.84	0.76	0.95	1

Supplementary Table 58: Quantile 0.95 of the crossed correlations among the 9 fields in Spain.

Min	OUTSP	INSP	CULT	CERE	COMM	INRE	OUTRE	DIST	MOBI
OUTSP	1	-0.28	-0.45	0.35	-0.17	-0.05	0.03	0.18	-0.15
INSP	-0.28	1	-0.50	-0.58	-0.21	0.10	-0.07	-0.01	-0.09
CULT	-0.45	-0.50	1	-0.31	-0.95	-0.54	-0.34	-0.49	-0.80
CERE	0.35	-0.58	-0.31	1	-0.09	-0.21	-0.23	-0.11	-0.45
COMM	-0.17	-0.21	-0.95	-0.09	1	0.06	-0.30	-0.06	0.17
INRE	-0.05	0.10	-0.54	-0.21	0.06	1	0.40	0.13	0.37
OUTRE	0.03	-0.07	-0.34	-0.23	-0.30	0.40	1	0.18	0.03
DIST	0.18	-0.01	-0.49	-0.11	-0.06	0.13	0.18	1	0.05
MOBI	-0.15	-0.09	-0.80	-0.45	0.17	0.37	0.03	0.05	1

Supplementary Table 59: Minimum of the crossed correlations among the 9 fields in Spain.

Max	OUTSP	INSP	CULT	CERE	COMM	INRE	OUTRE	DIST	MOBI
OUTSP	1	1	0.99	0.96	0.97	0.89	0.97	0.95	0.92
INSP	1	1	0.97	0.95	0.96	0.98	0.98	0.88	0.95
CULT	0.99	0.97	1	0.90	0.99	0.92	0.99	0.93	0.88
CERE	0.96	0.95	0.90	1	0.89	0.95	0.93	0.92	0.98
COMM	0.97	0.96	0.99	0.89	1	0.96	0.98	0.97	0.92
INRE	0.89	0.98	0.92	0.95	0.96	1	0.97	0.92	0.89
OUTRE	0.97	0.98	0.99	0.93	0.98	0.97	1	0.86	0.90
DIST	0.95	0.88	0.93	0.92	0.97	0.92	0.86	1	0.96
MOBI	0.92	0.95	0.88	0.98	0.92	0.89	0.90	0.96	1

Supplementary Table 60: Maximun of the crossed correlations among the 9 fields in Spain.

$Q_3 - Q_1$	OUTSP	INSP	CULT	CERE	COMM	INRE	OUTRE	DIST	MOBI
OUTSP	0	0.10	0.15	0.20	0.29	0.13	0.44	0.41	0.27
INSP	0.10	0	0.33	0.17	0.19	0.17	0.43	0.34	0.21
CULT	0.15	0.33	0	0.43	0.54	0.13	0.39	0.34	0.26
CERE	0.20	0.17	0.43	0	0.19	0.30	0.56	0.41	0.29
COMM	0.29	0.19	0.54	0.19	0	0.17	0.53	0.31	0.34
INRE	0.13	0.17	0.13	0.30	0.17	0	0.15	0.24	0.18
OUTRE	0.44	0.43	0.39	0.56	0.53	0.15	0	0.25	0.38
DIST	0.41	0.34	0.34	0.41	0.31	0.24	0.25	0	0.20
MOBI	0.27	0.21	0.26	0.29	0.34	0.18	0.38	0.20	0

Supplementary Table 61: Interquartile range of the crossed correlations among the 9 fields in Spain.

Appendix VII. Scatter plots for the mean strigency index and the 7-day COVID-19 logarithmic return of the incidence growth rate 12 days delayed (LR_{t+12}) in each province and Spain.

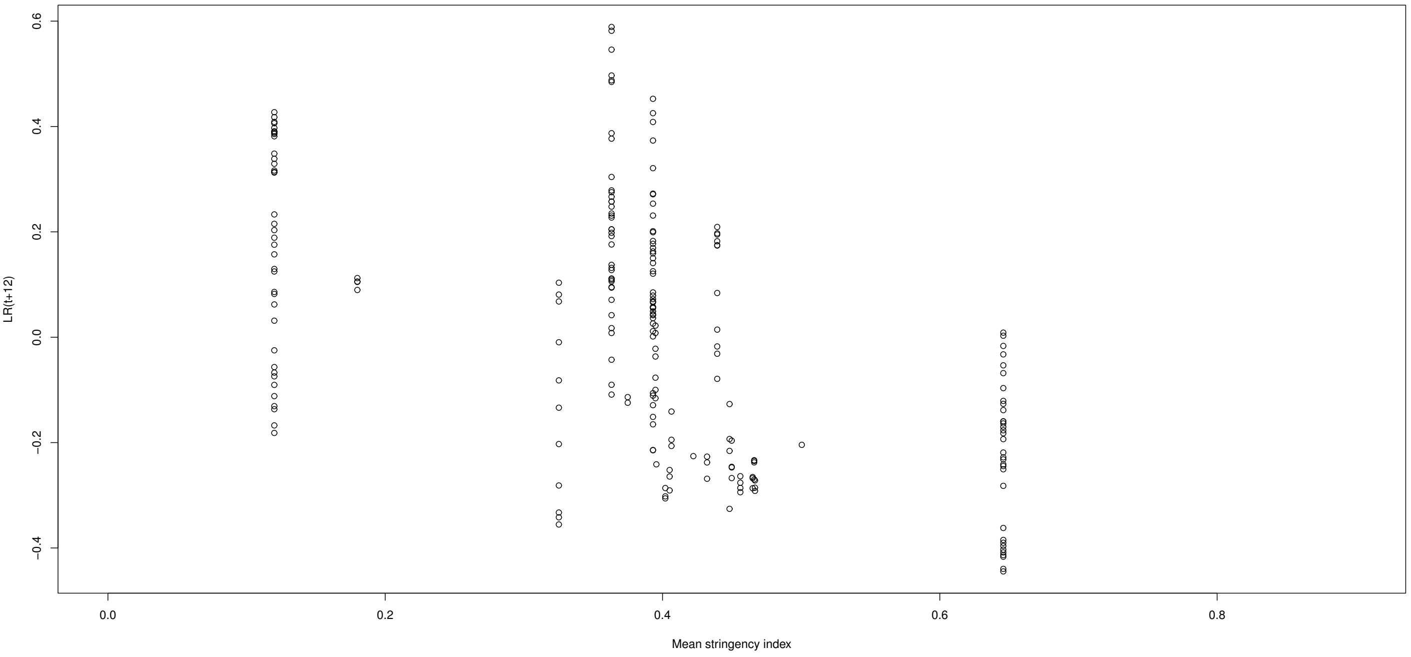

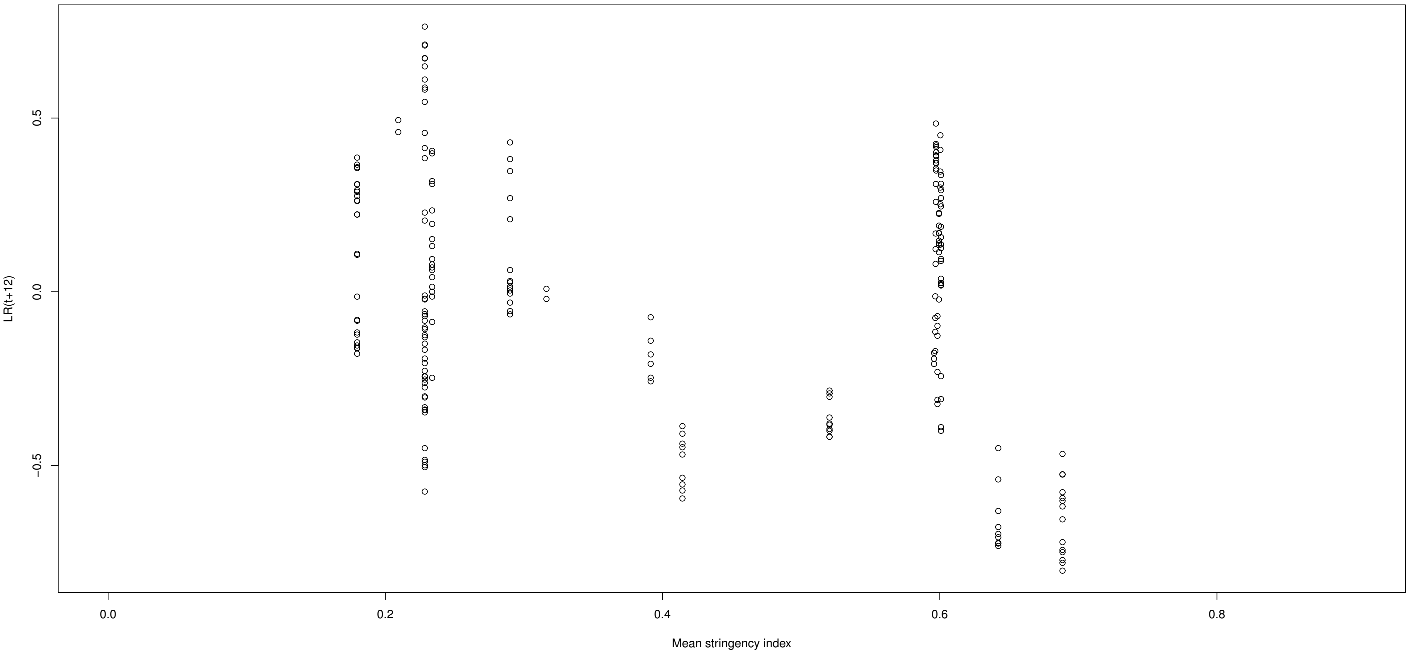

Supplementary Figure 1: Scatter plots for the mean strigency index and LR_{t+12} for Álava (top) and Albacete (bottom).

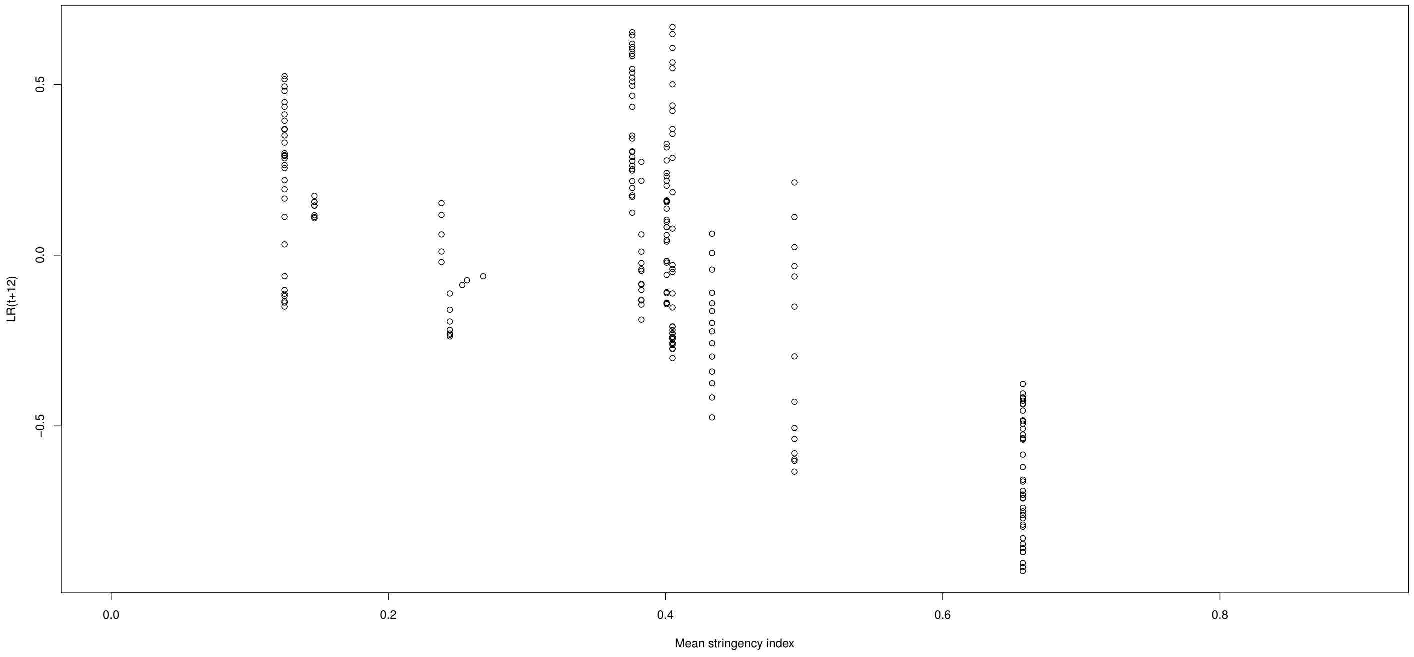

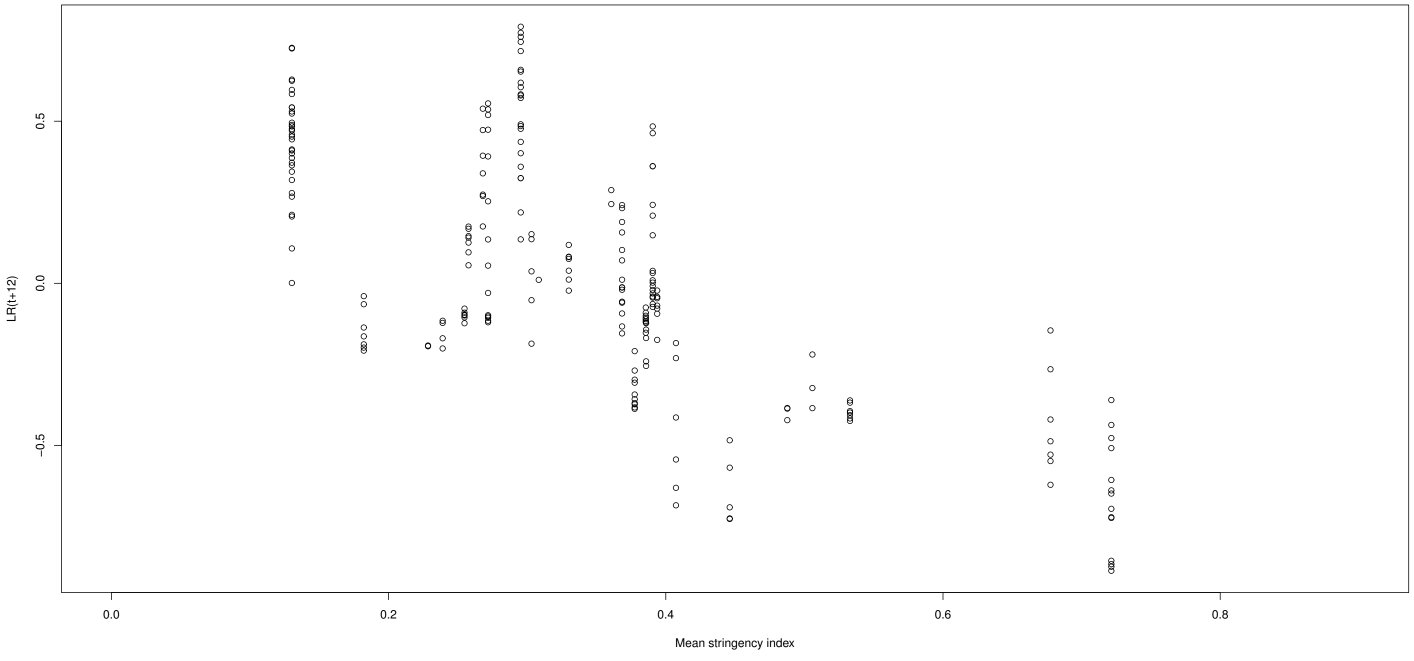

Supplementary Figure 2: Scatter plots for the mean strigency index and LR_{t+12} for Alicante (top) and Almería (bottom).

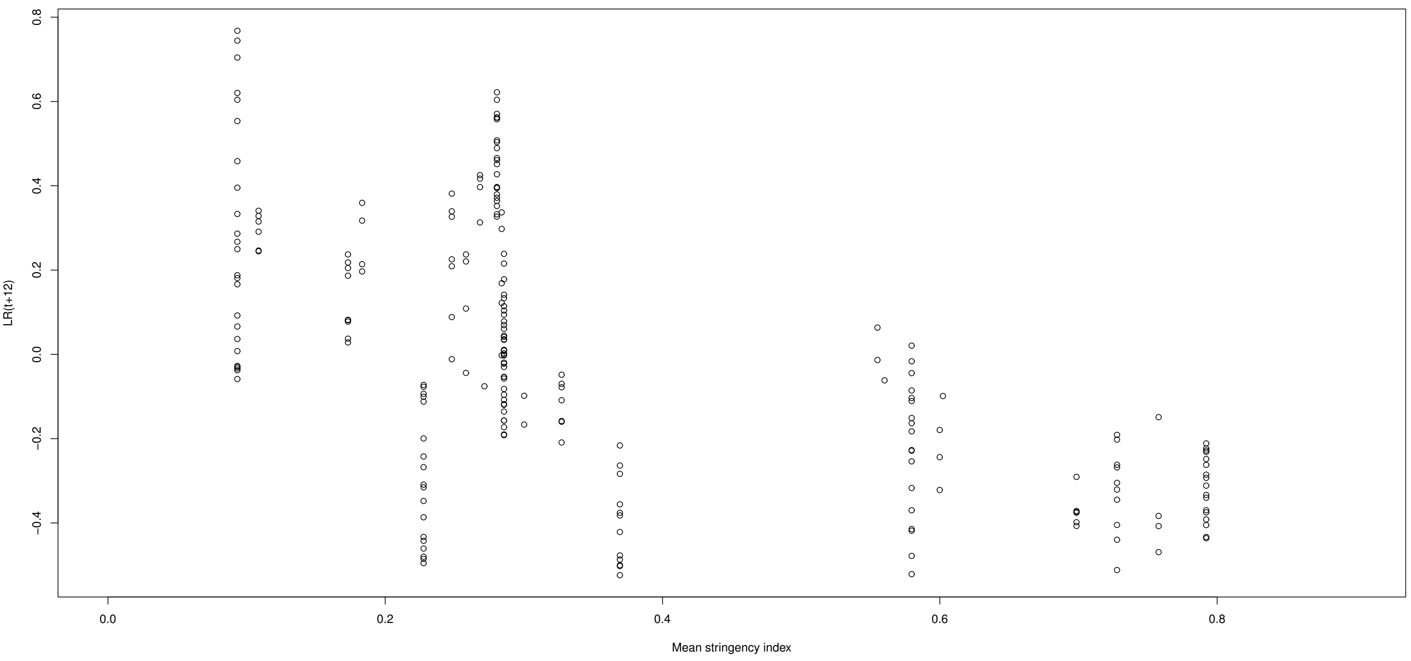

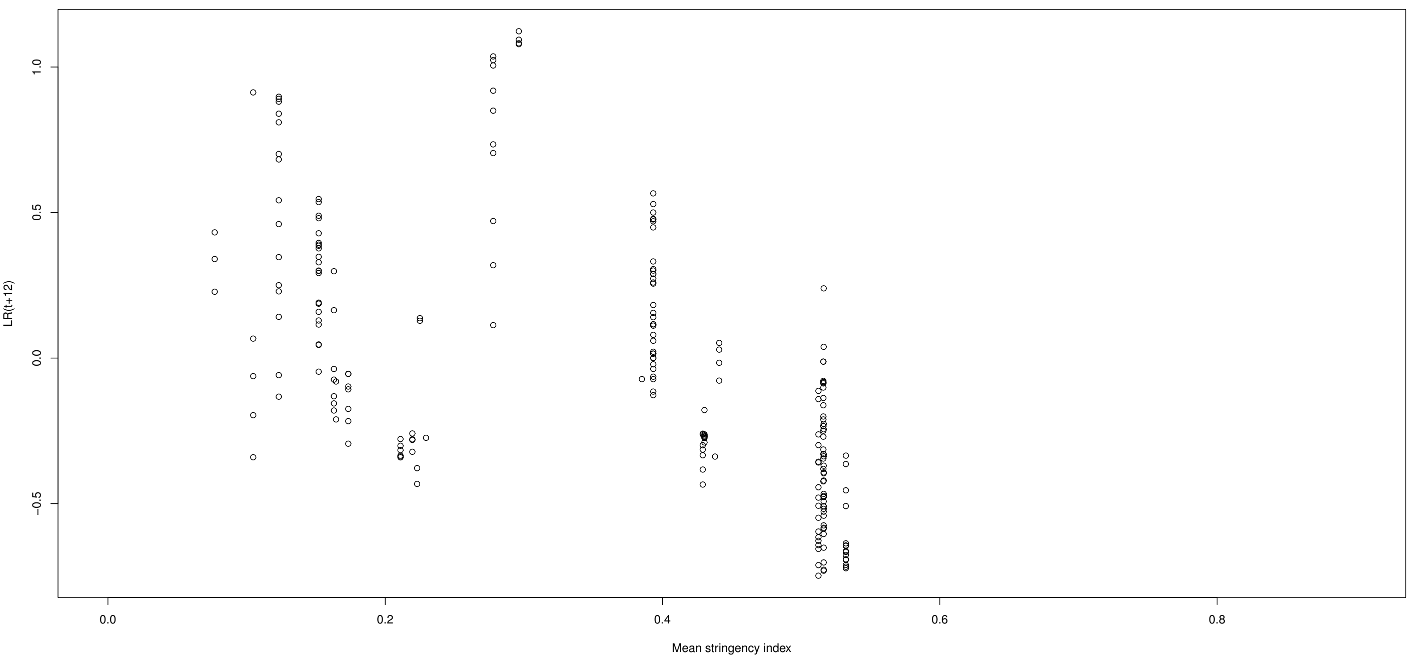

Supplementary Figure 3: Scatter plots for the mean strigency index and LR_{t+12} for Asturias (top) and Ávila (bottom).

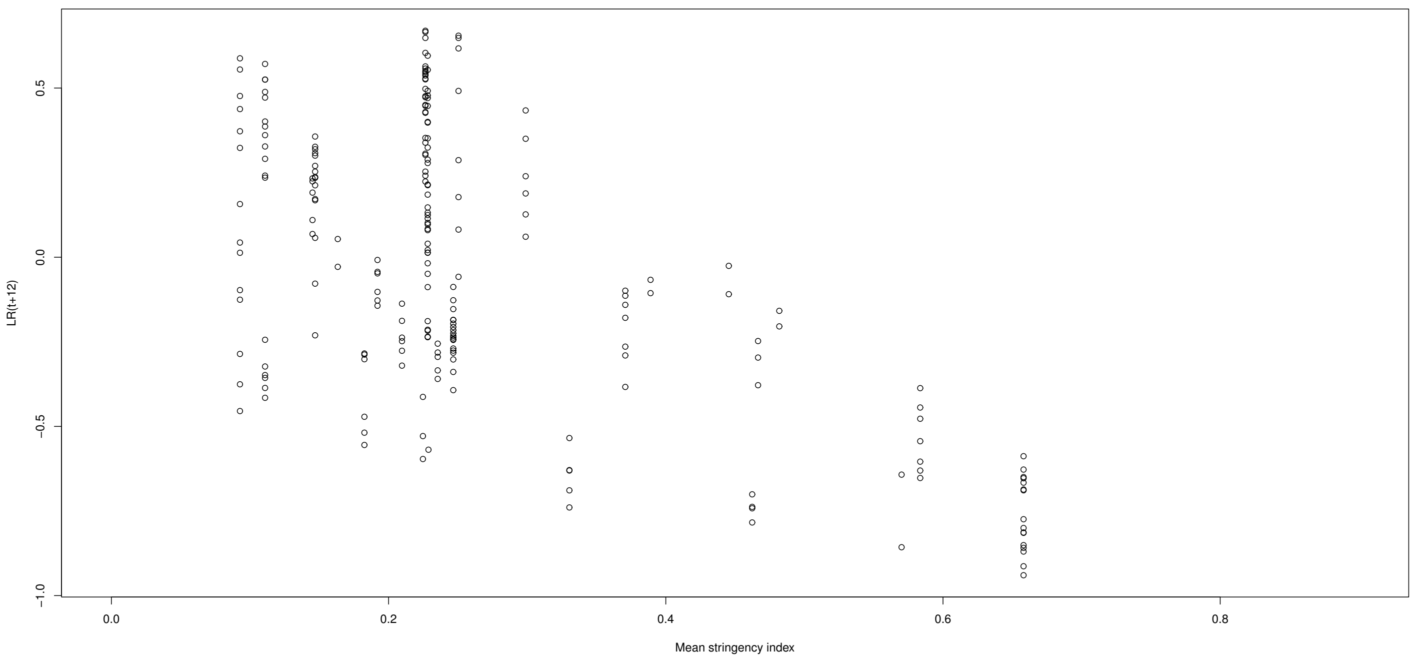

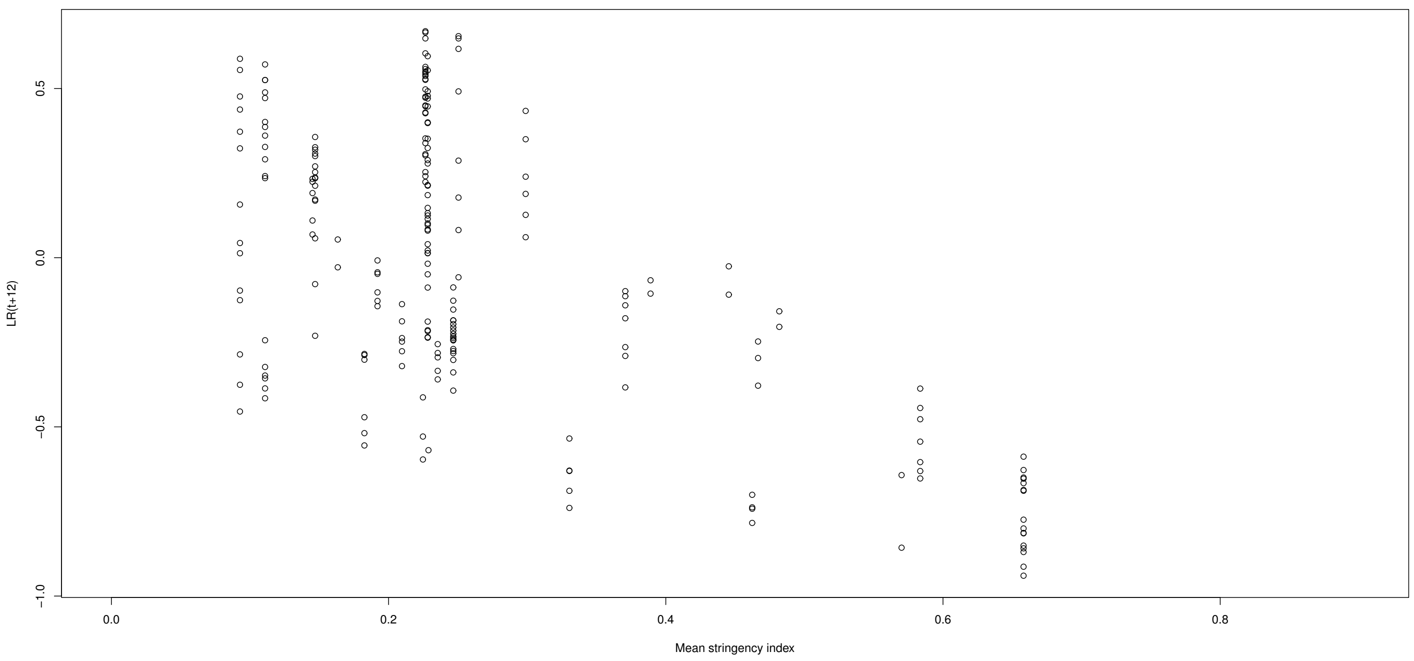

Supplementary Figure 4: Scatter plots for the mean strigency index and LR_{t+12} for Badajoz (top) and Illes Balears (bottom).

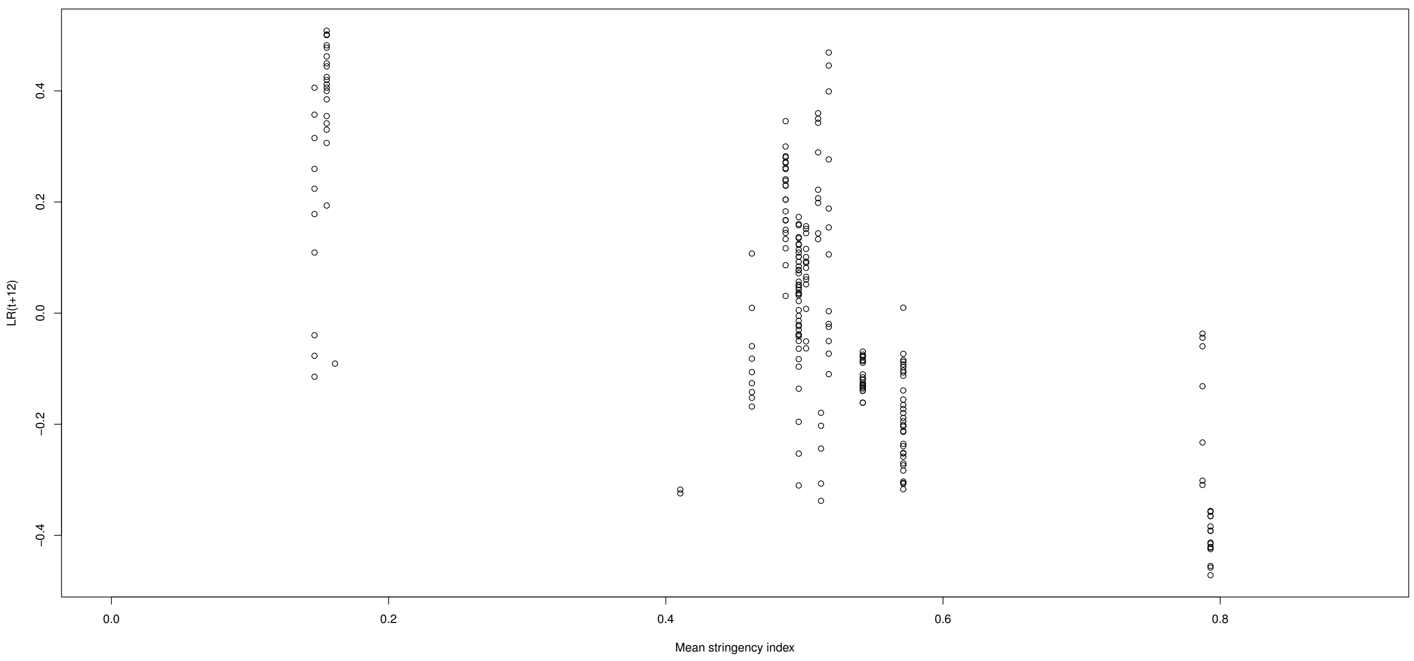

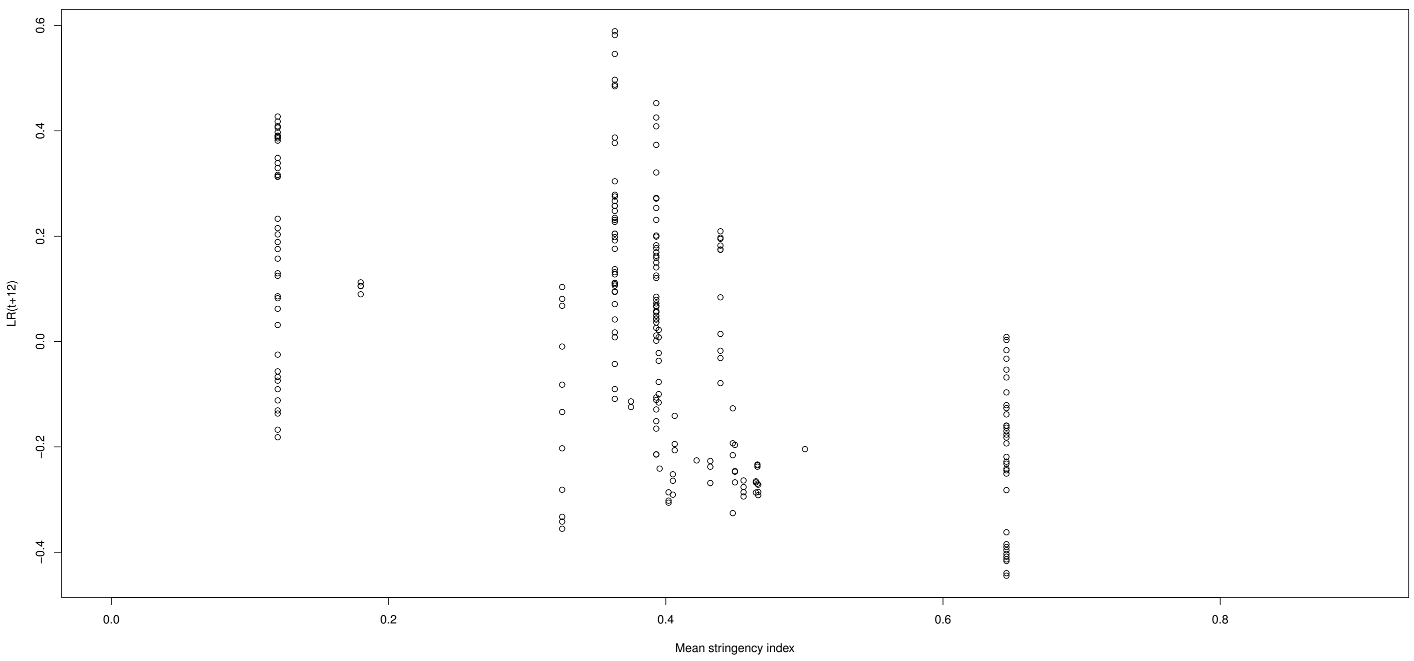

Supplementary Figure 5: Scatter plots for the mean strigency index and LR_{t+12} for Barcelona (top) and Bizkaia (bottom).

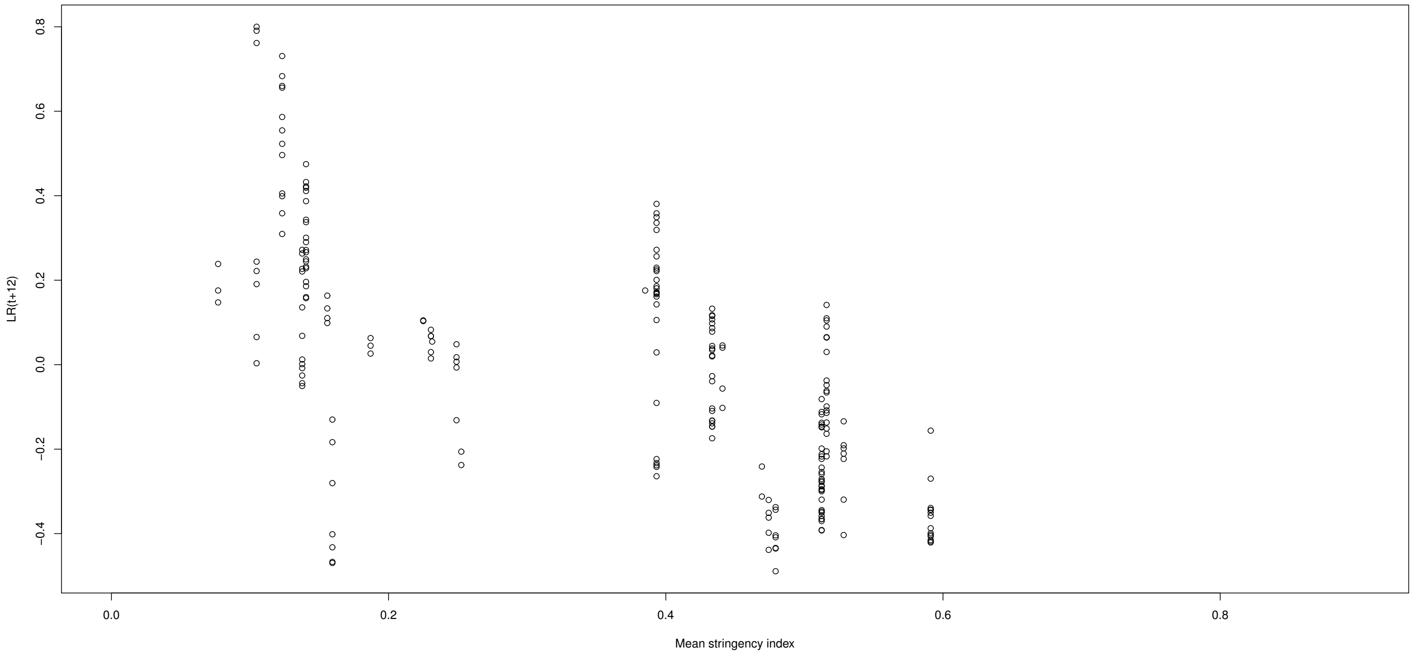

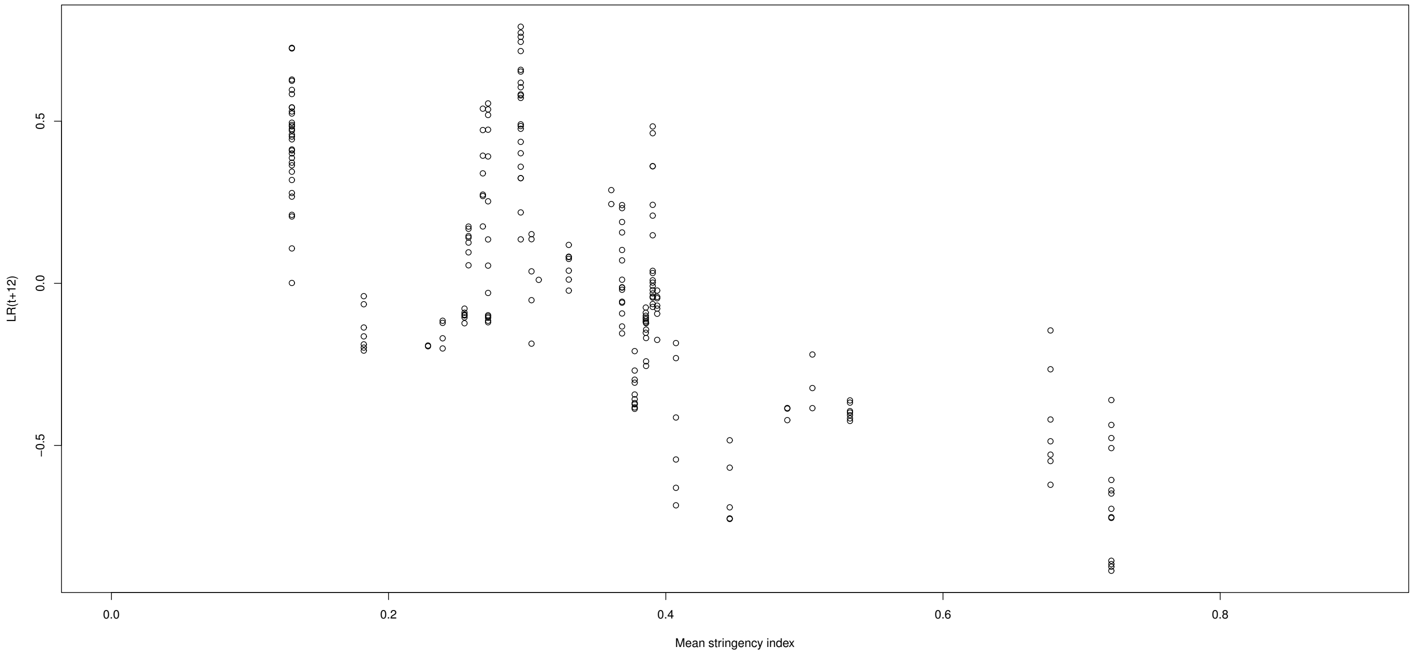

Supplementary Figure 6: Scatter plots for the mean strigency index and LR_{t+12} for Burgos (top) and Cáceres (bottom).

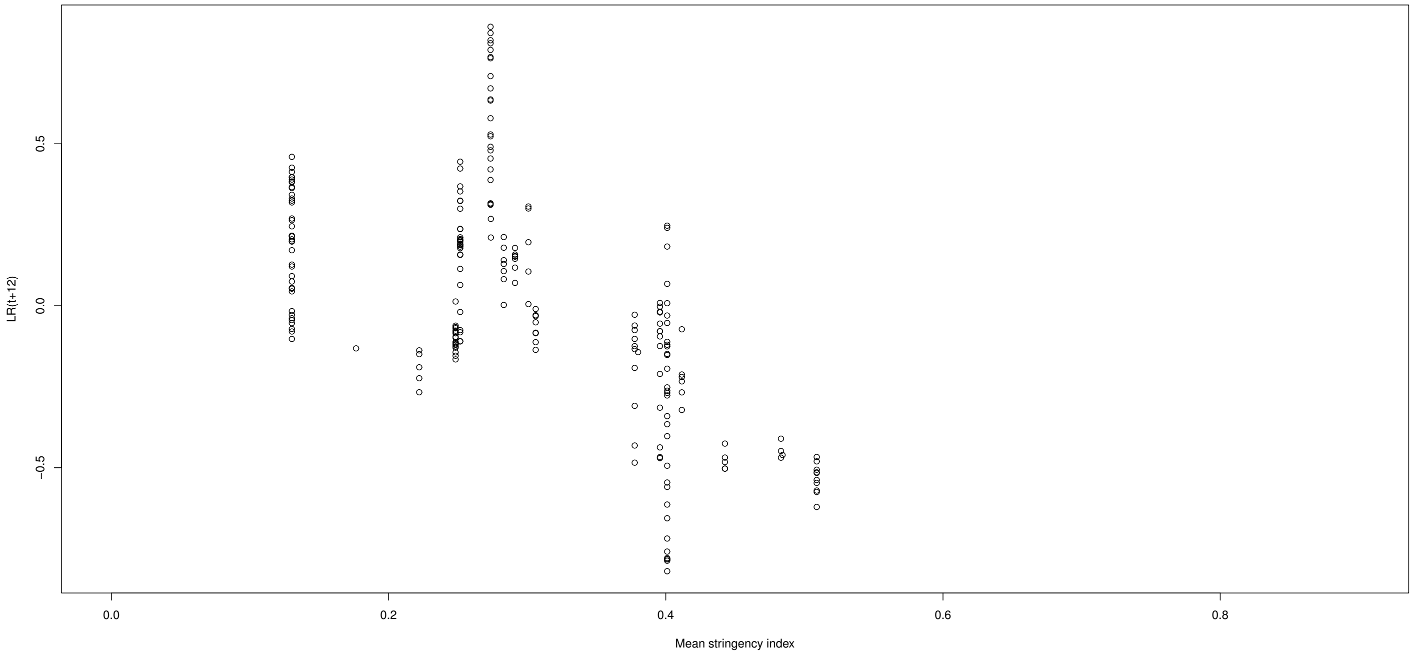

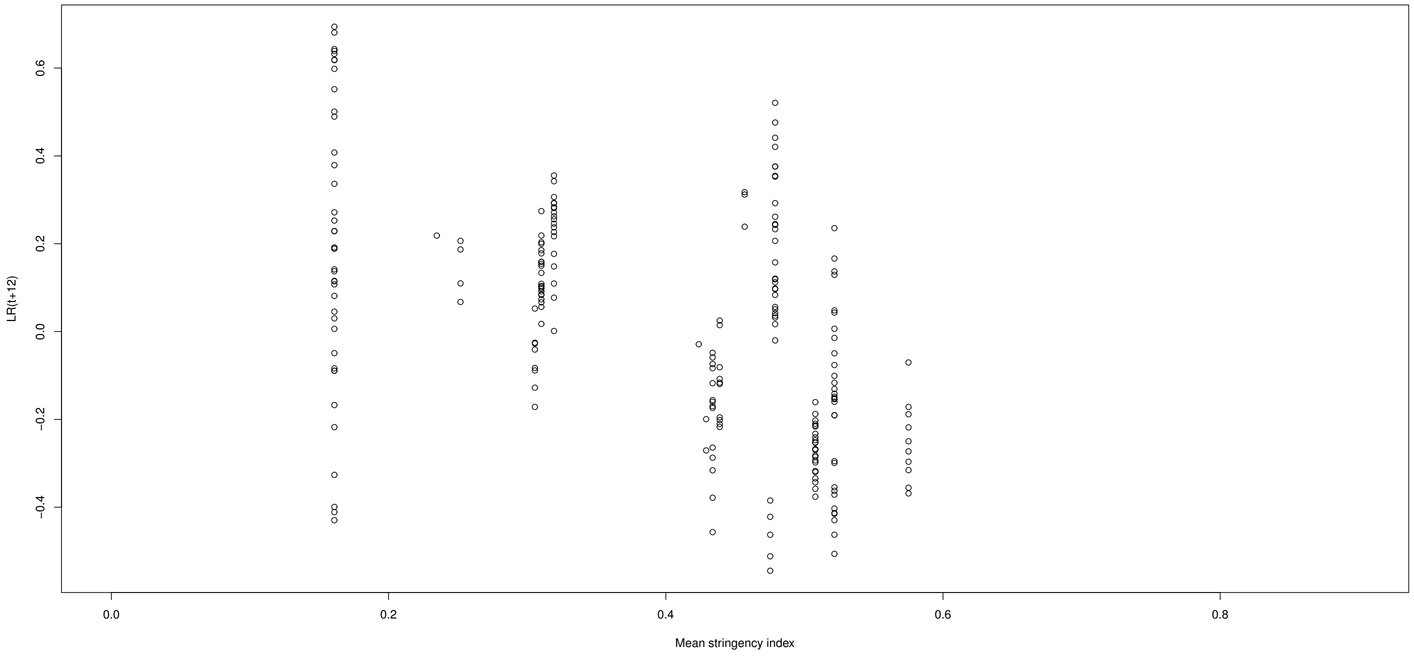

Supplementary Figure 7: Scatter plots for the mean strigency index and LR_{t+12} for Cádiz (top) and Cantabria (bottom).

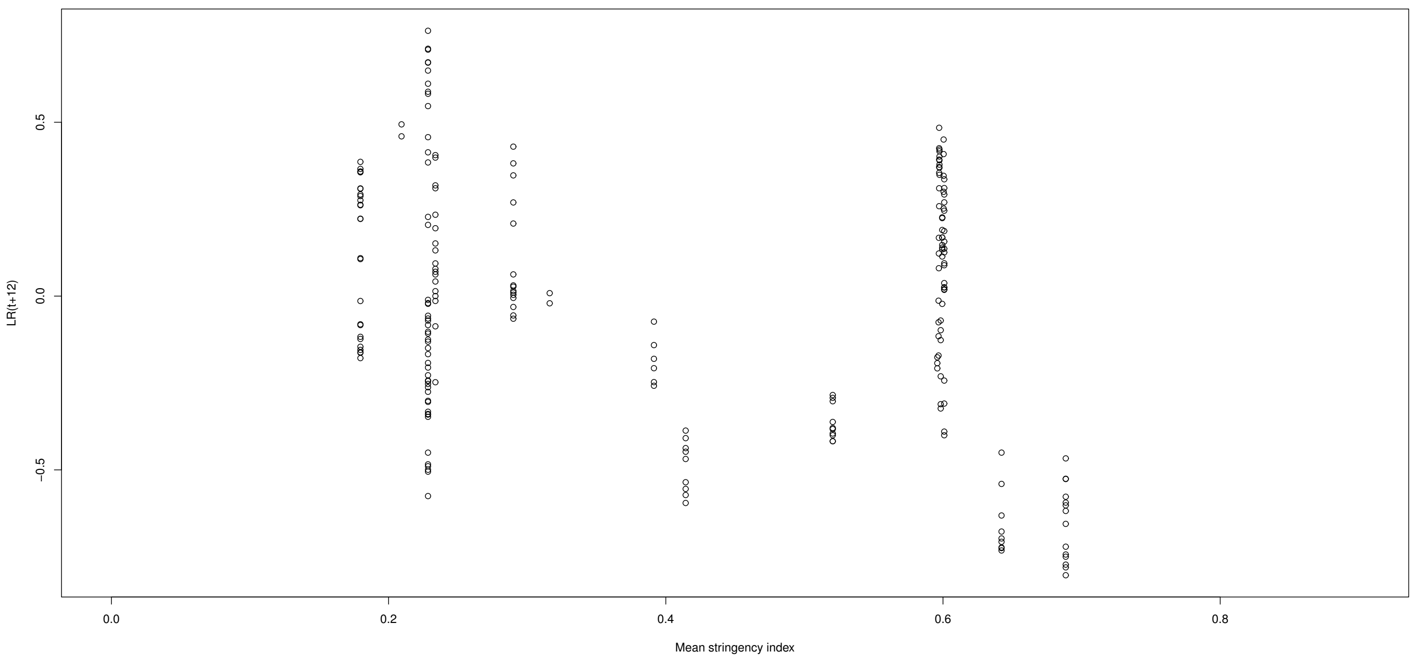

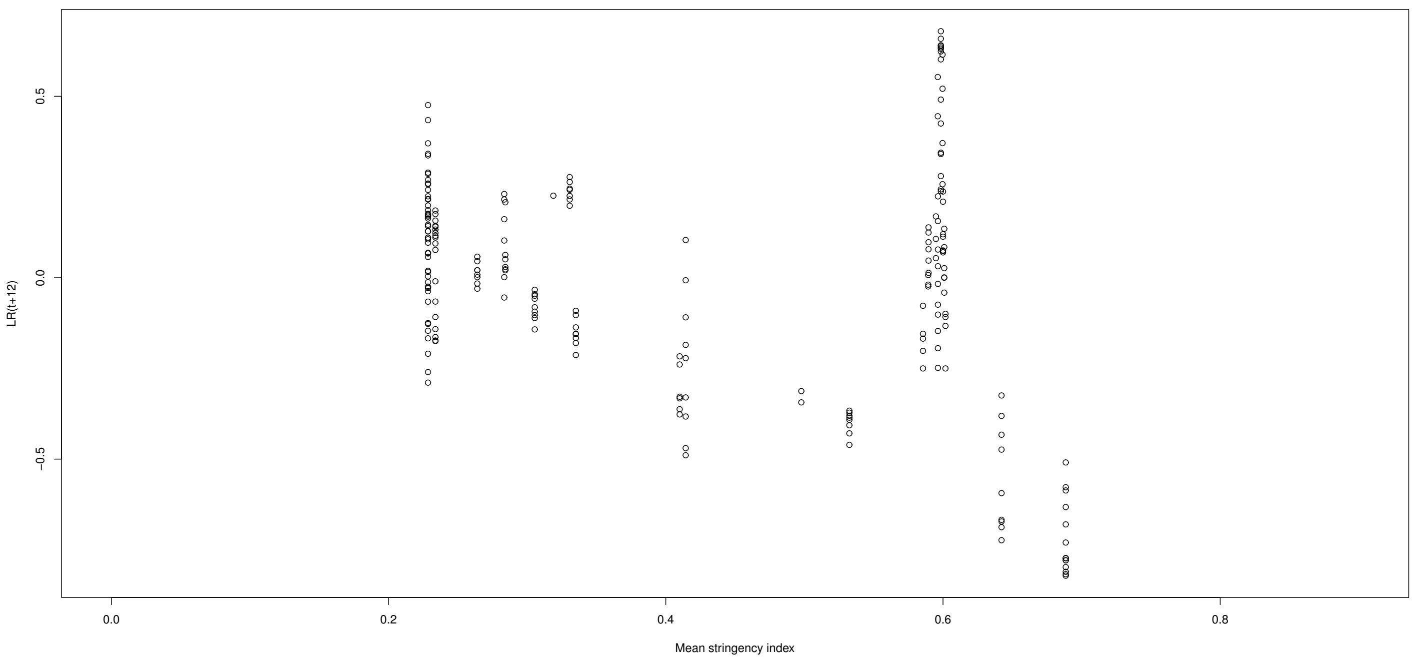

Supplementary Figure 8: Scatter plots for the mean strigency index and LR_{t+12} for Castellón (top) and Ciudad Real (bottom).

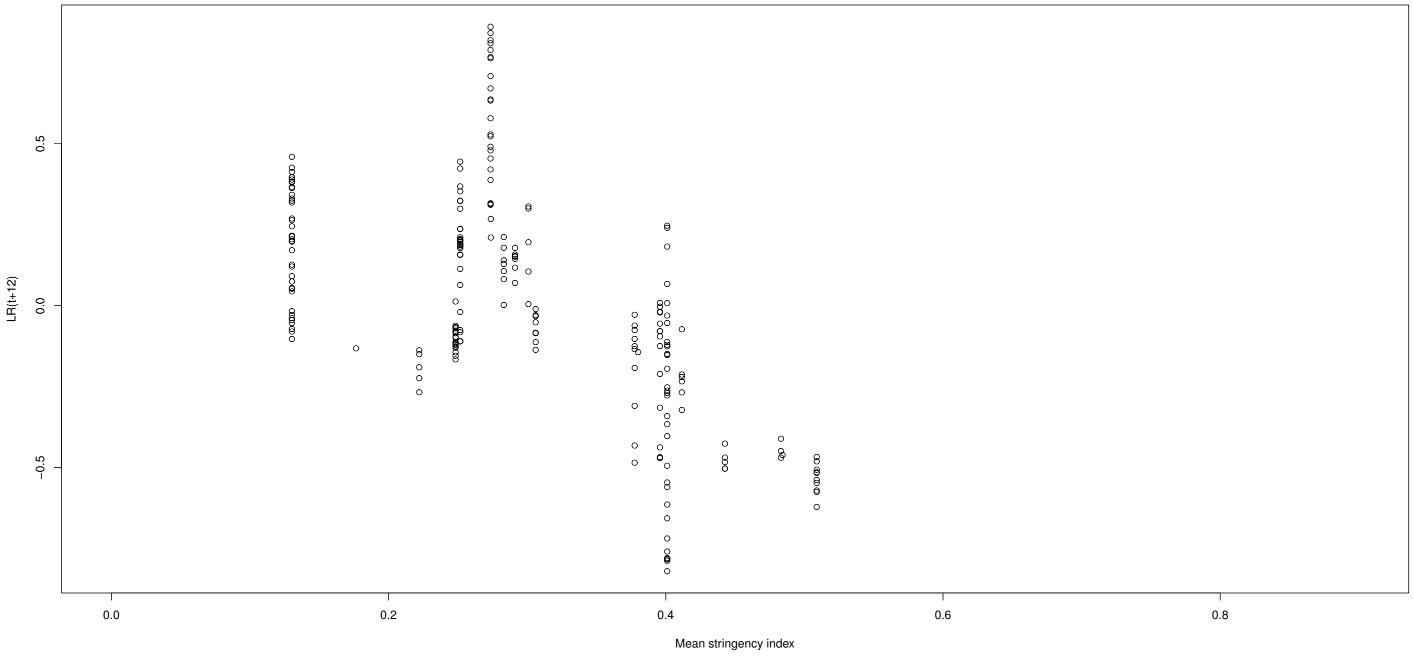

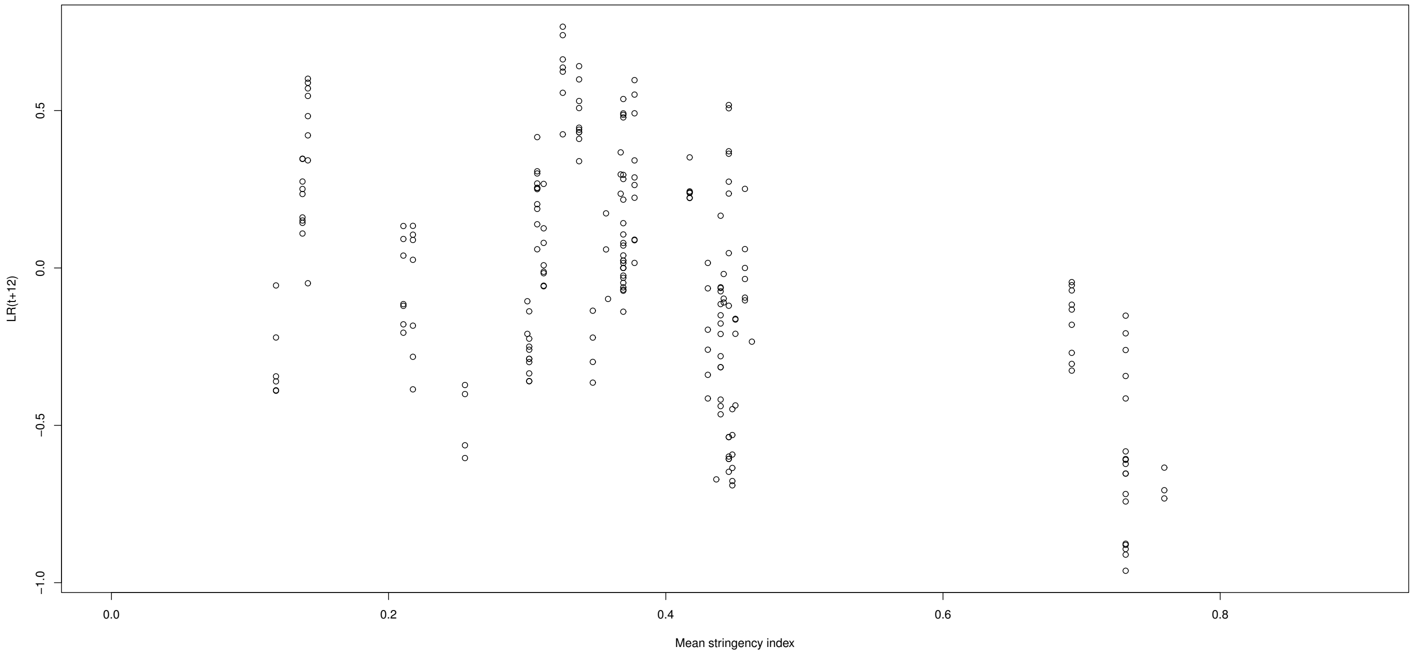

Supplementary Figure 9: Scatter plots for the mean strigency index and LR_{t+12} for Córdoba (top) and A Coruña (bottom).

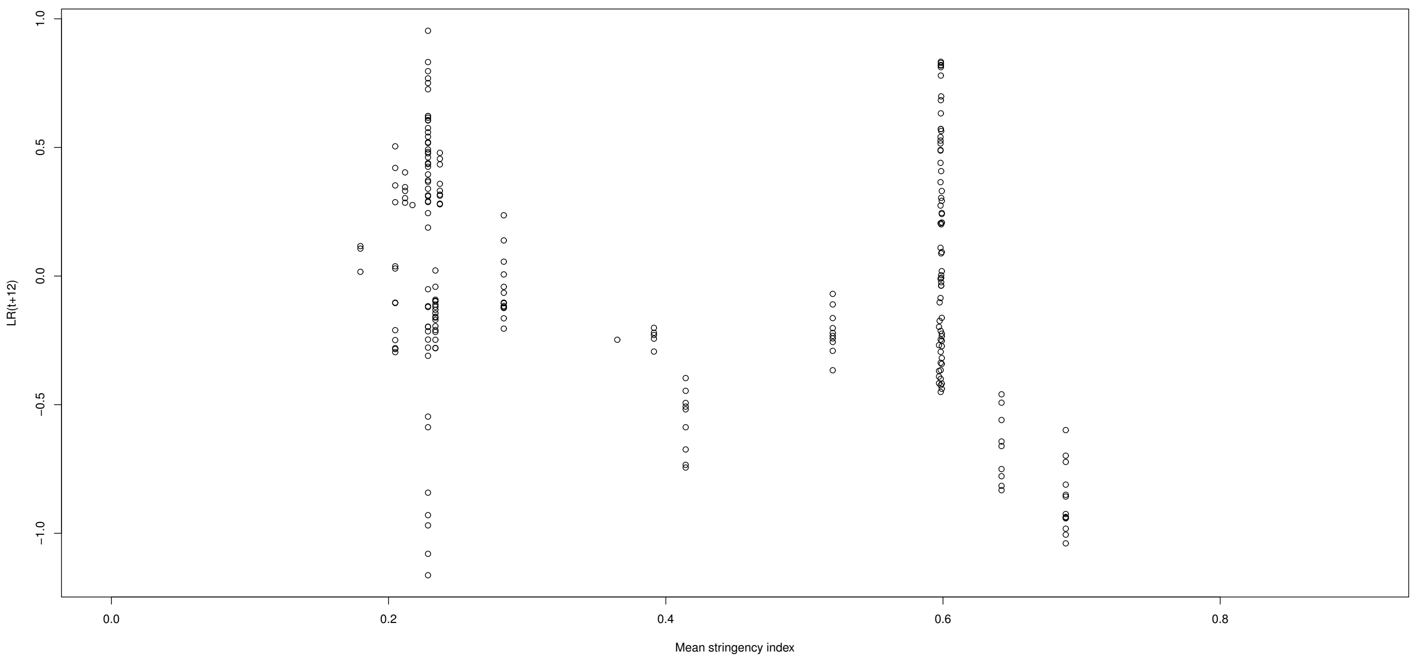

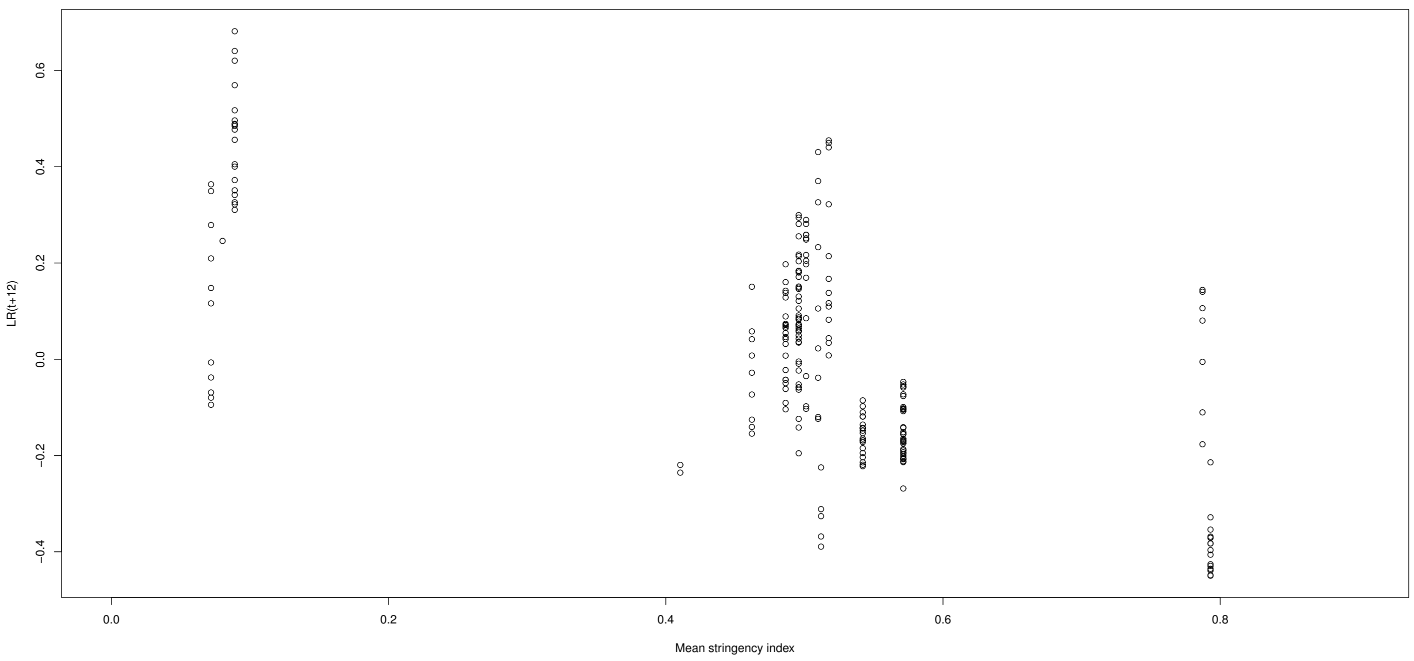

Supplementary Figure 10: Scatter plots for the mean strigency index and LR_{t+12} for Cuenca (top) and Girona (bottom).

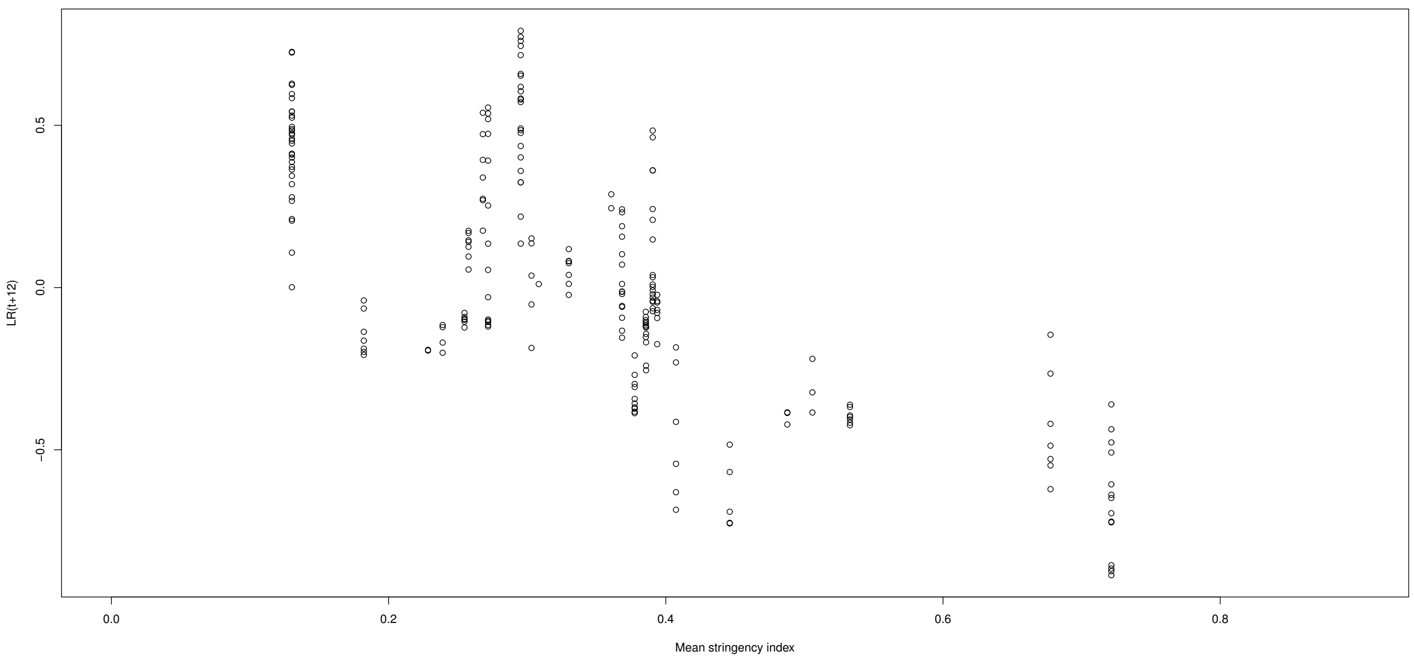

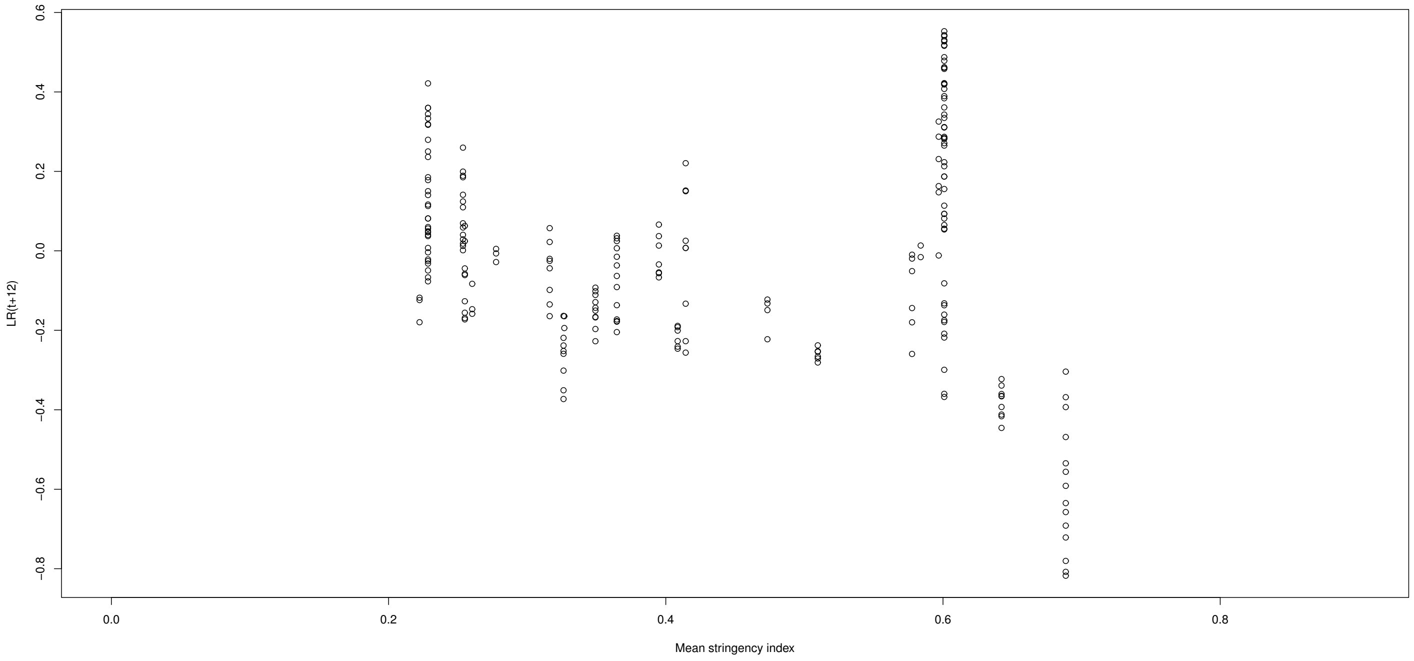

Supplementary Figure 11: Scatter plots for the mean strigency index and LR_{t+12} for Granada (top) and Guadalajara (bottom).

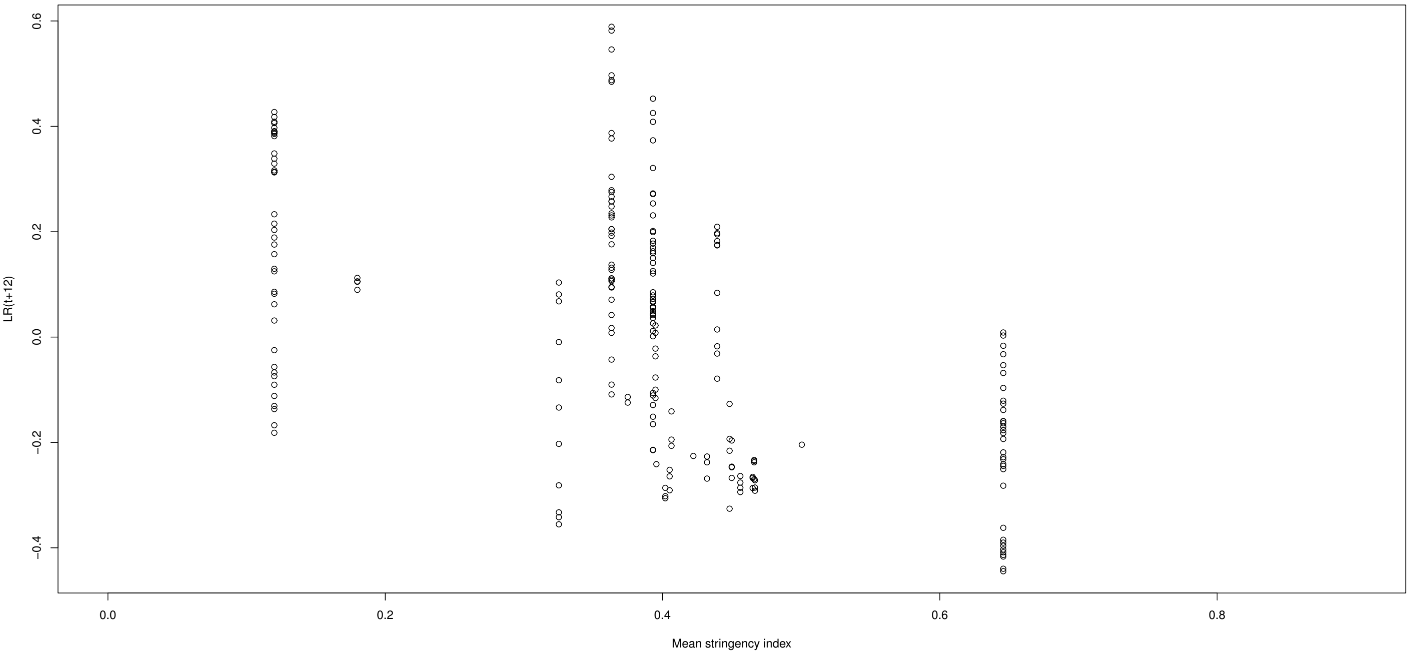

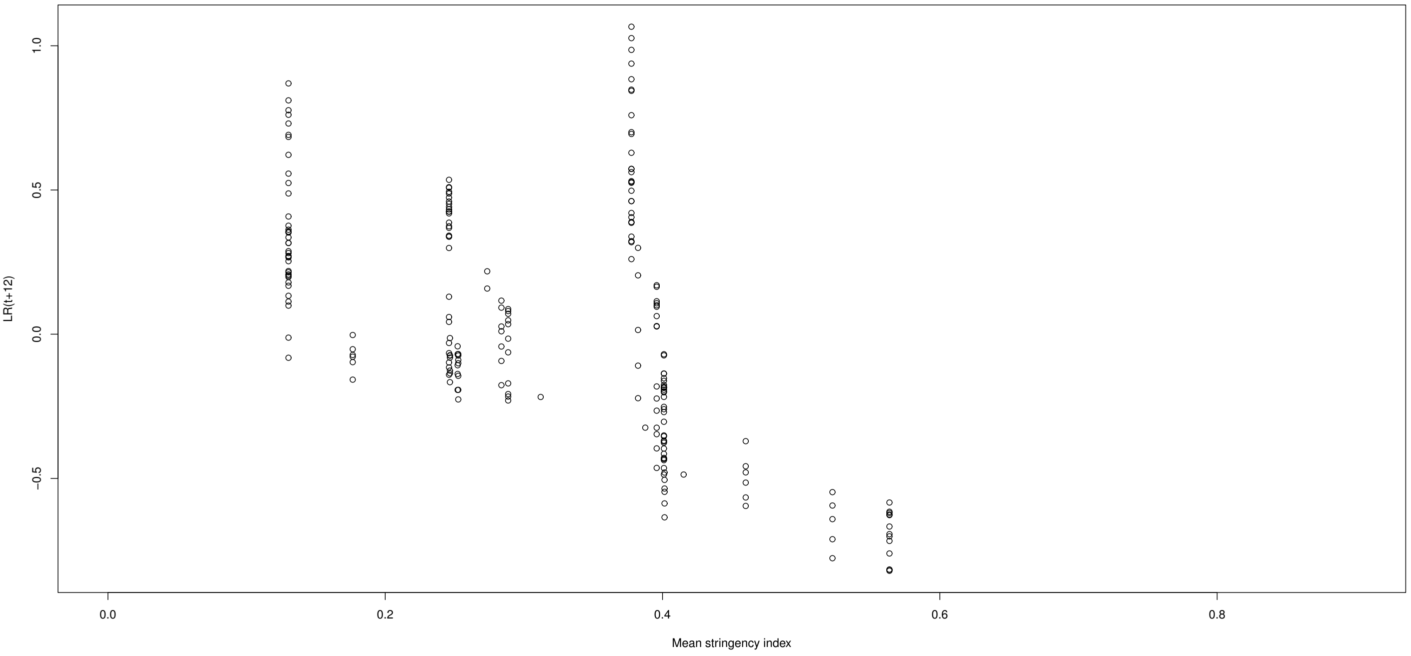

Supplementary Figure 12: Scatter plots for the mean strigency index and LR_{t+12} for Guipuzkoa (top) and Huelva (bottom).

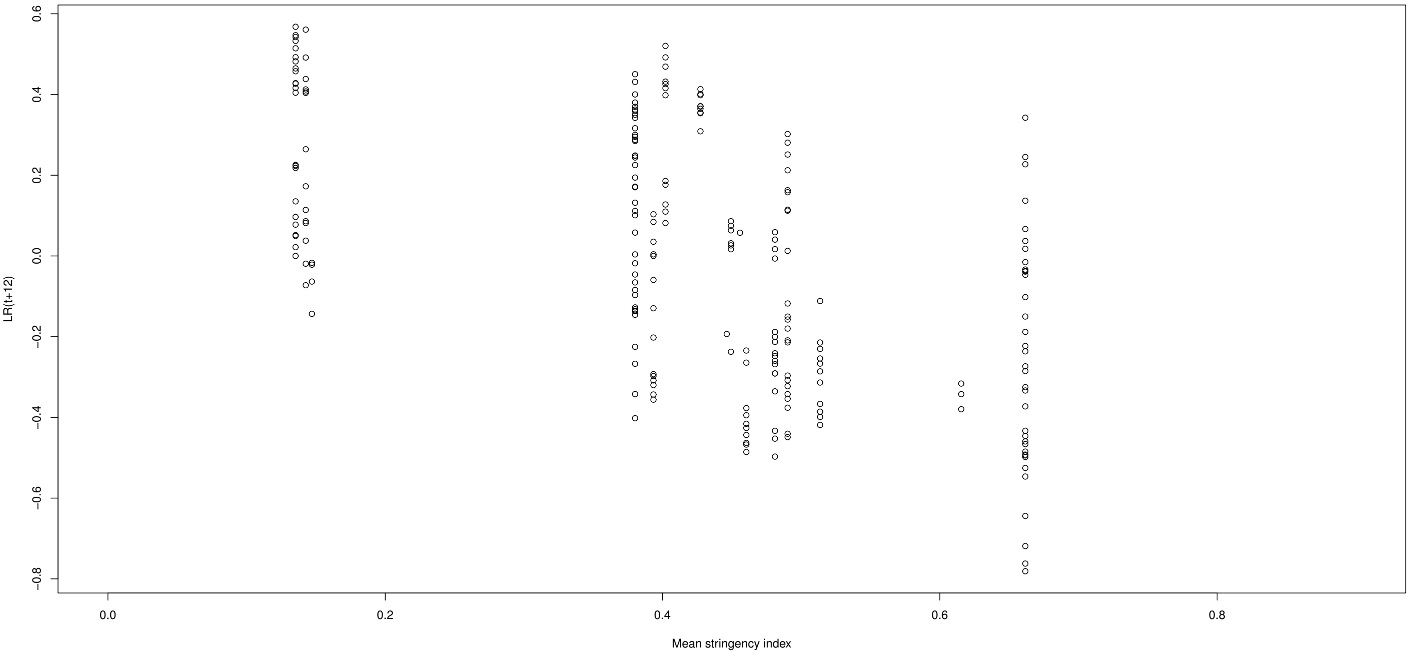

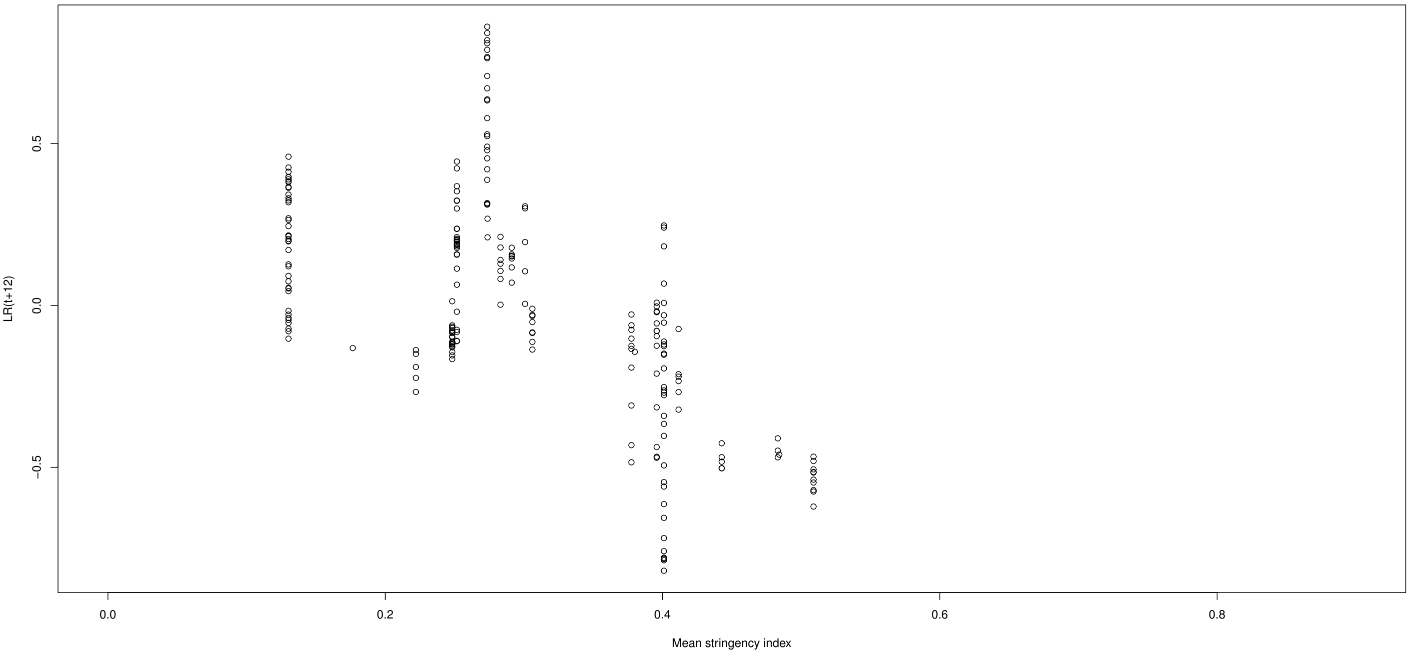

Supplementary Figure 13: Scatter plots for the mean strigency index and LR_{t+12} for Huesca (top) and Jaén (bottom).

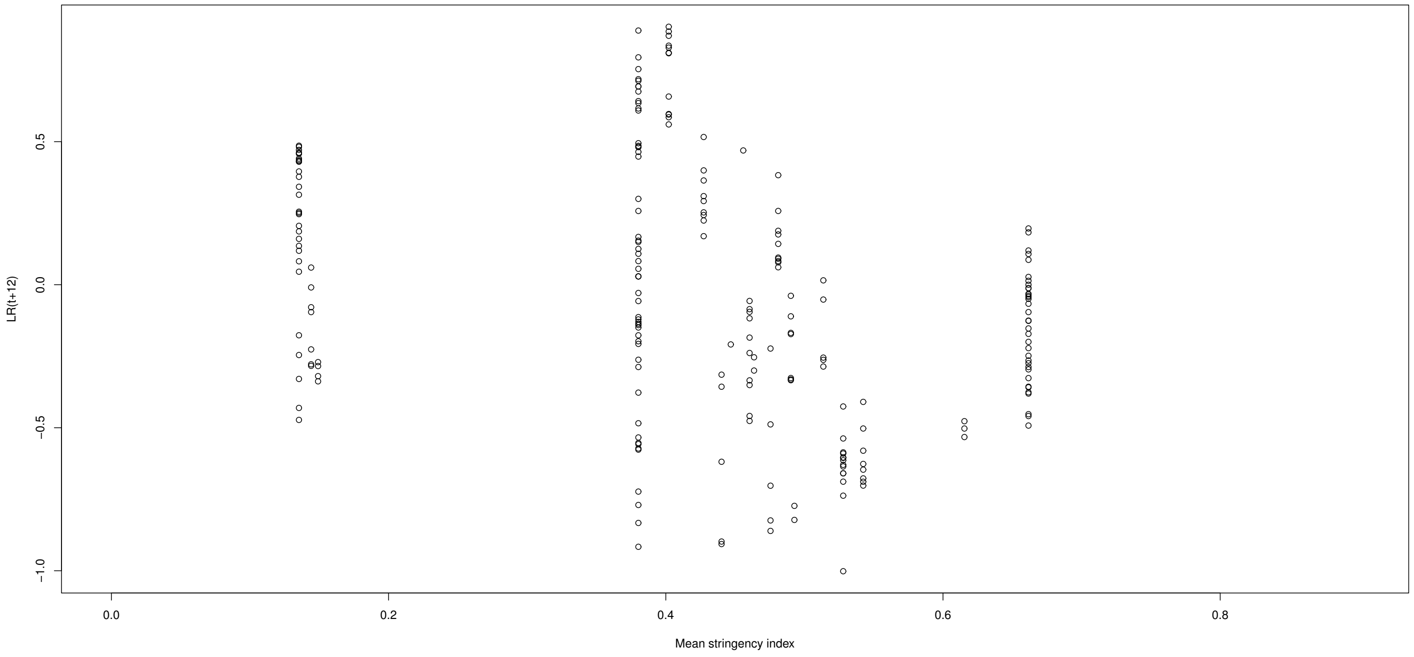

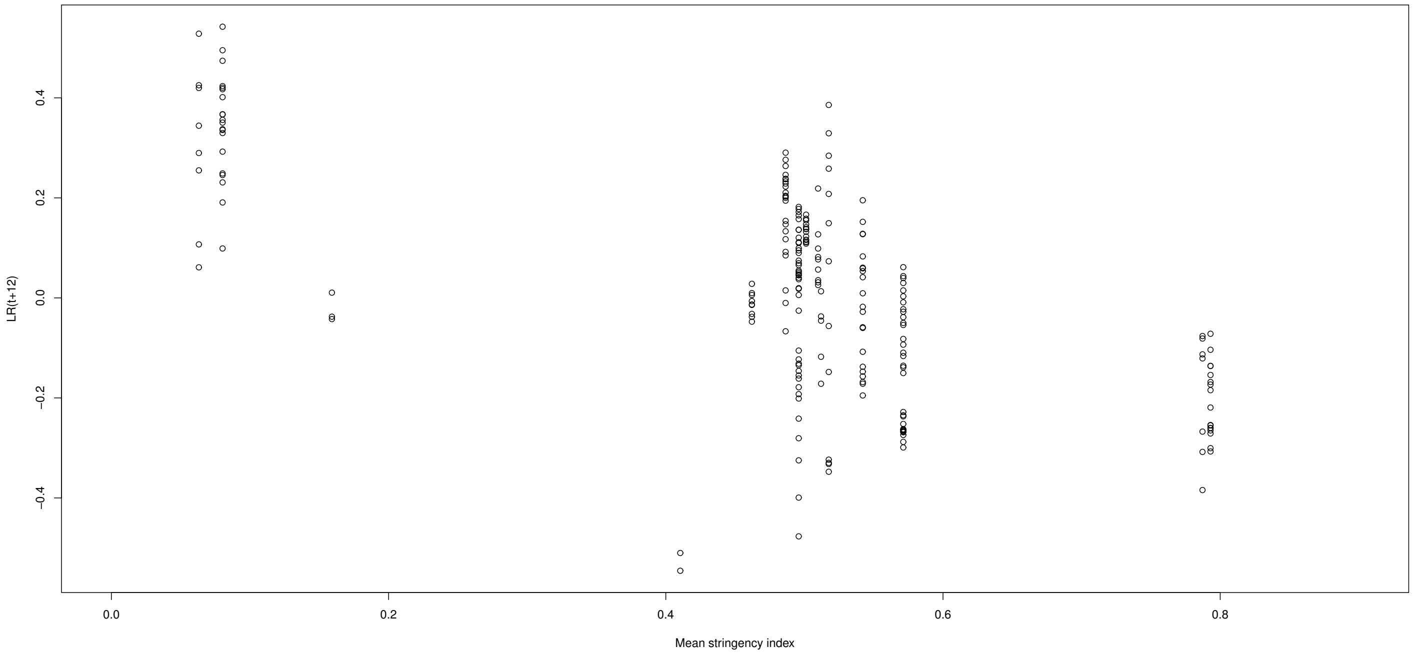

Supplementary Figure 14: Scatter plots for the mean strigency index and LR_{t+12} for León (top) and Lleida (bottom).

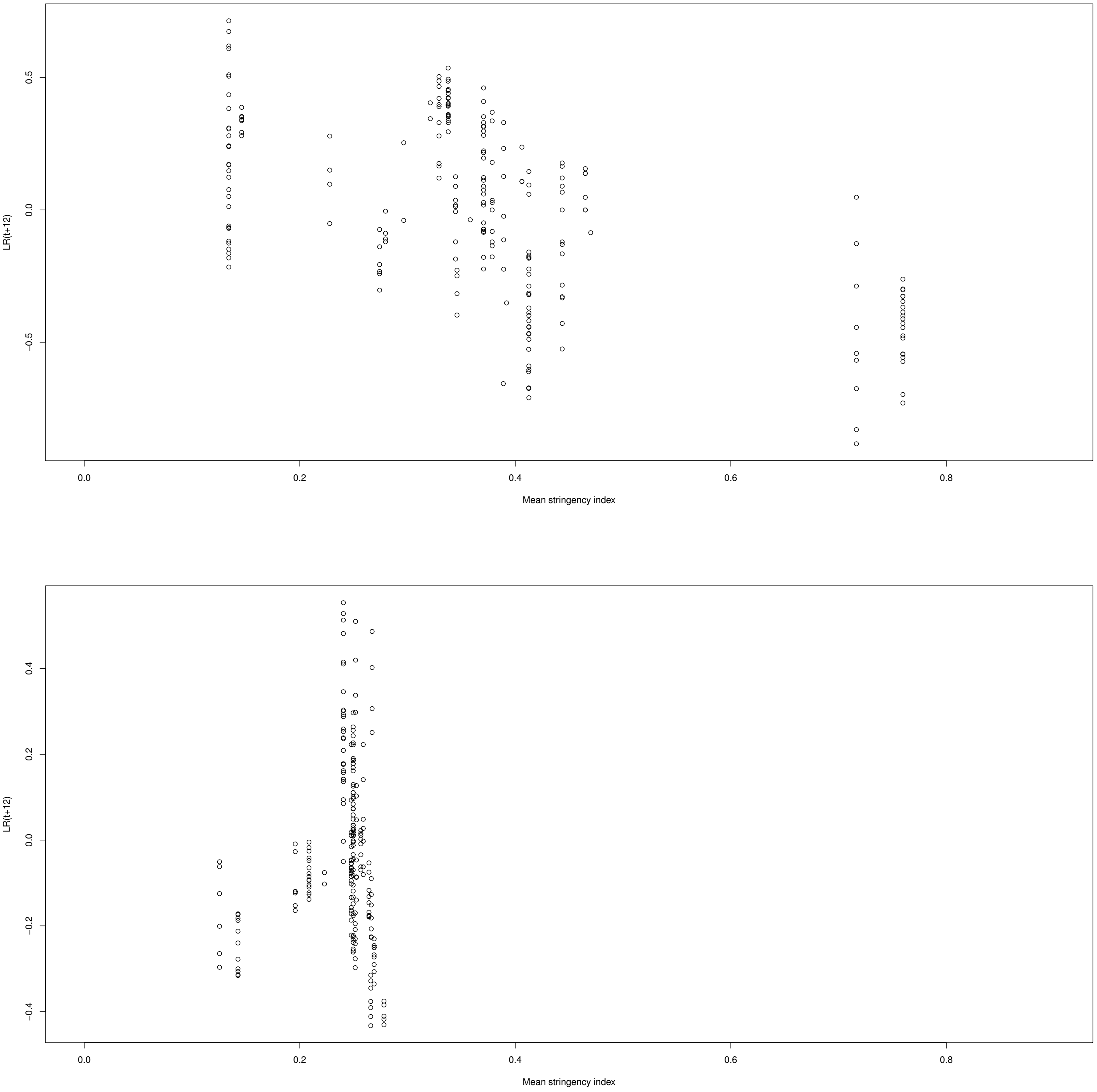

Supplementary Figure 15: Scatter plots for the mean strigency index and LR_{t+12} for Lugo (top) and Madrid (bottom).

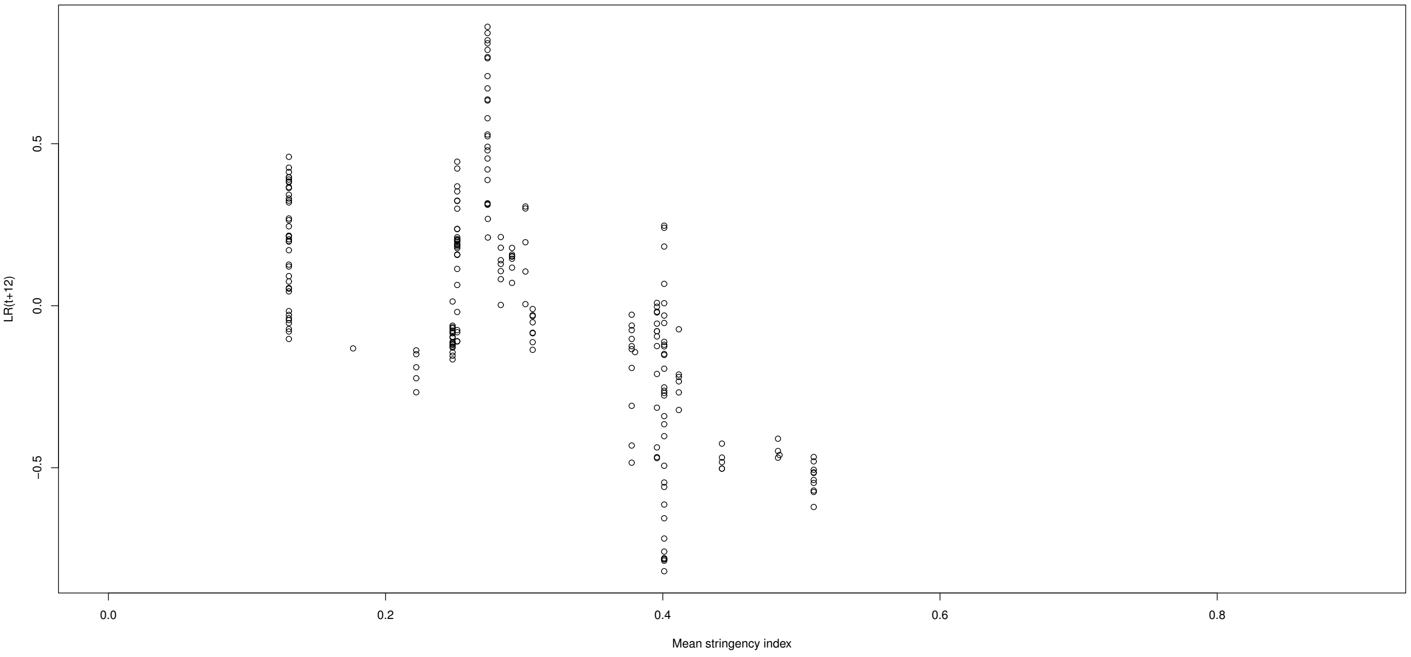

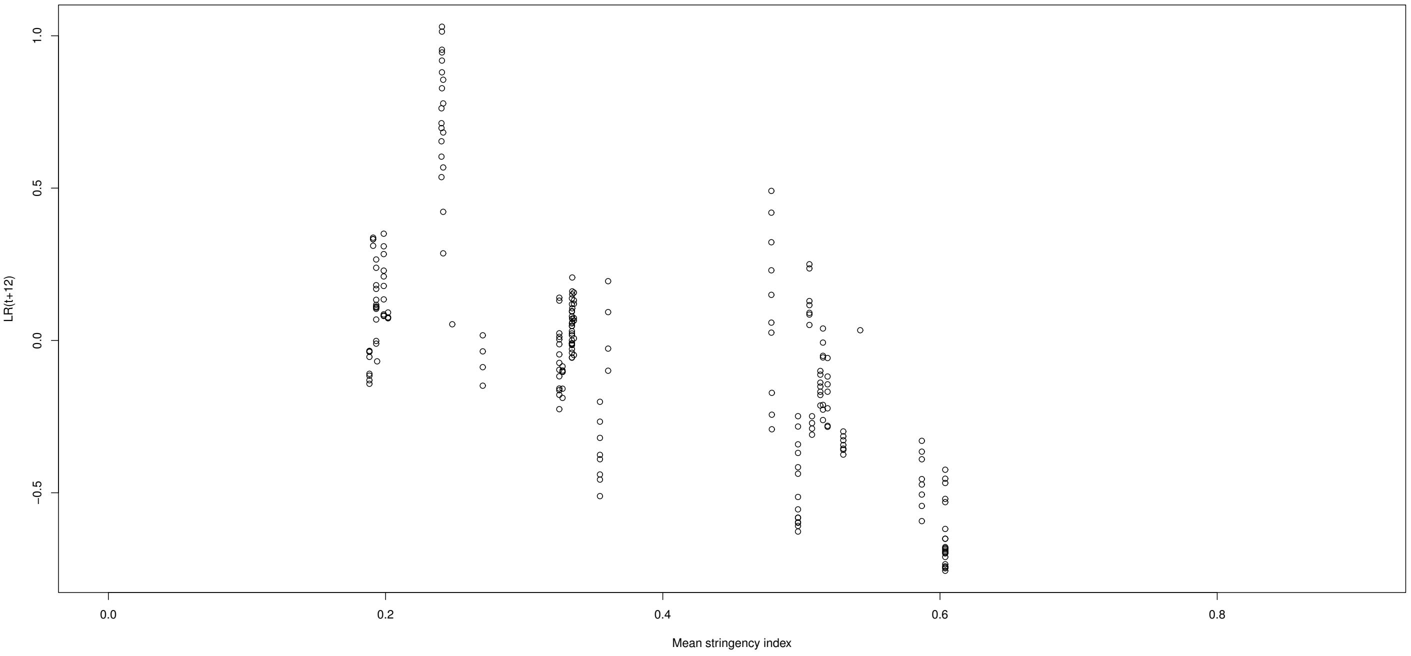

Supplementary Figure 16: Scatter plots for the mean strigency index and LR_{t+12} for Málaga (top) and Murcia (bottom).

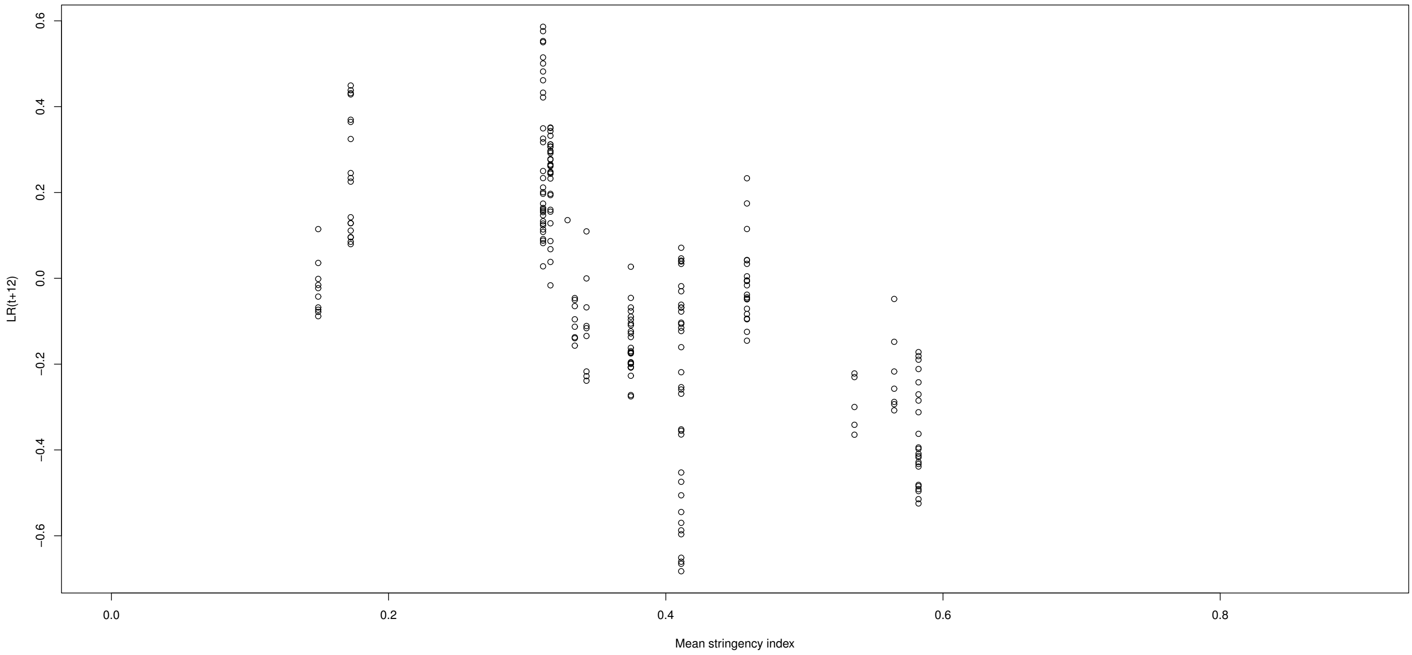

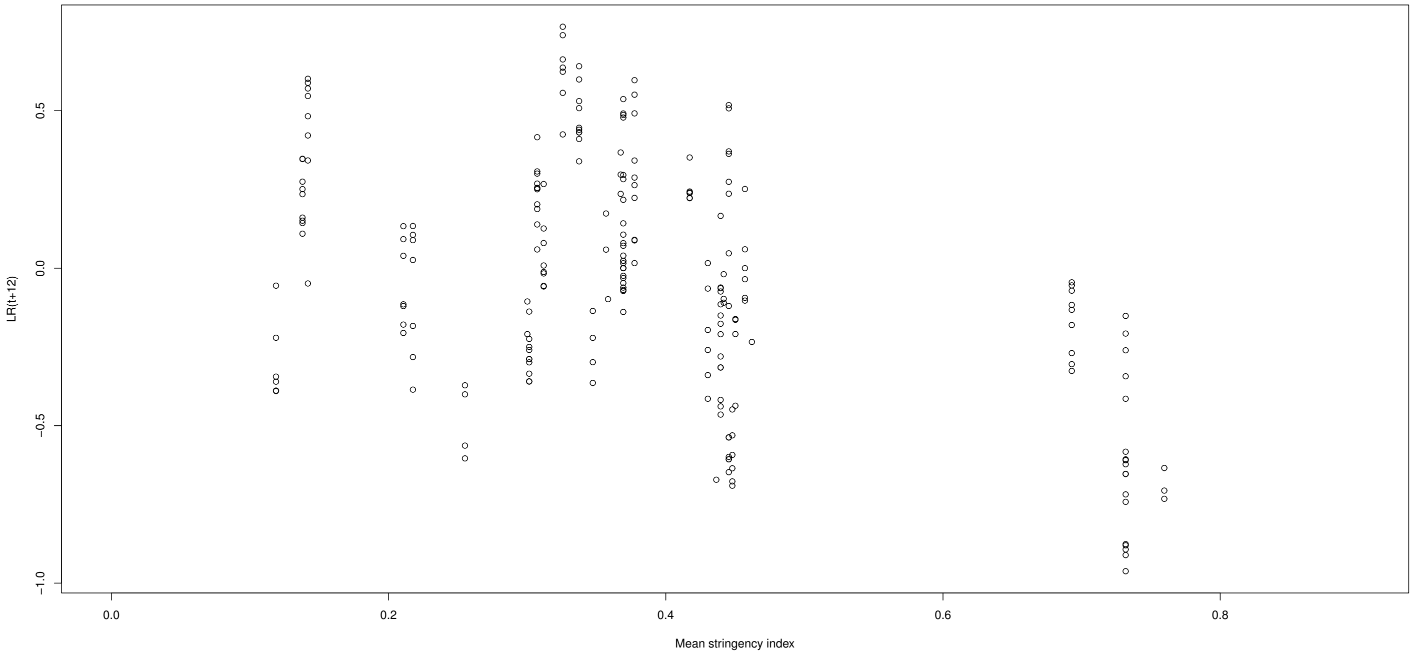

Supplementary Figure 17: Scatter plots for the mean strigency index and LR_{t+12} for Navarra (top) and Ourense (bottom).

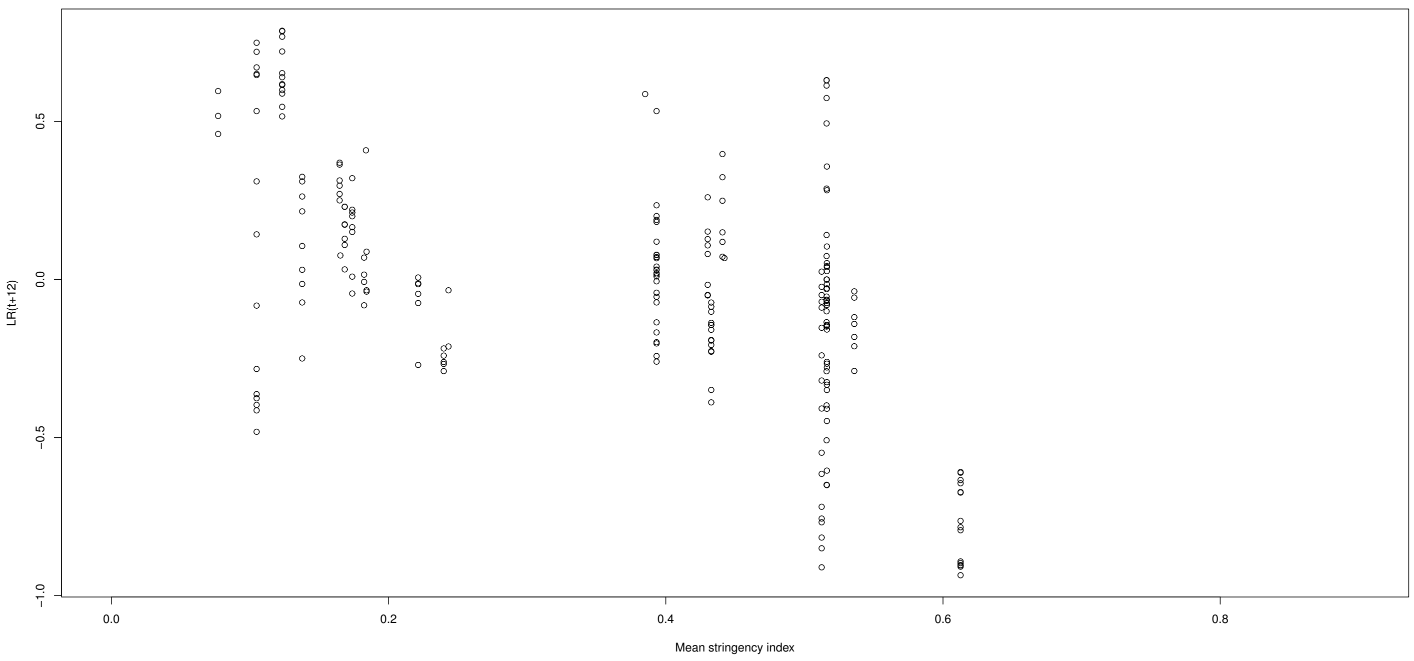

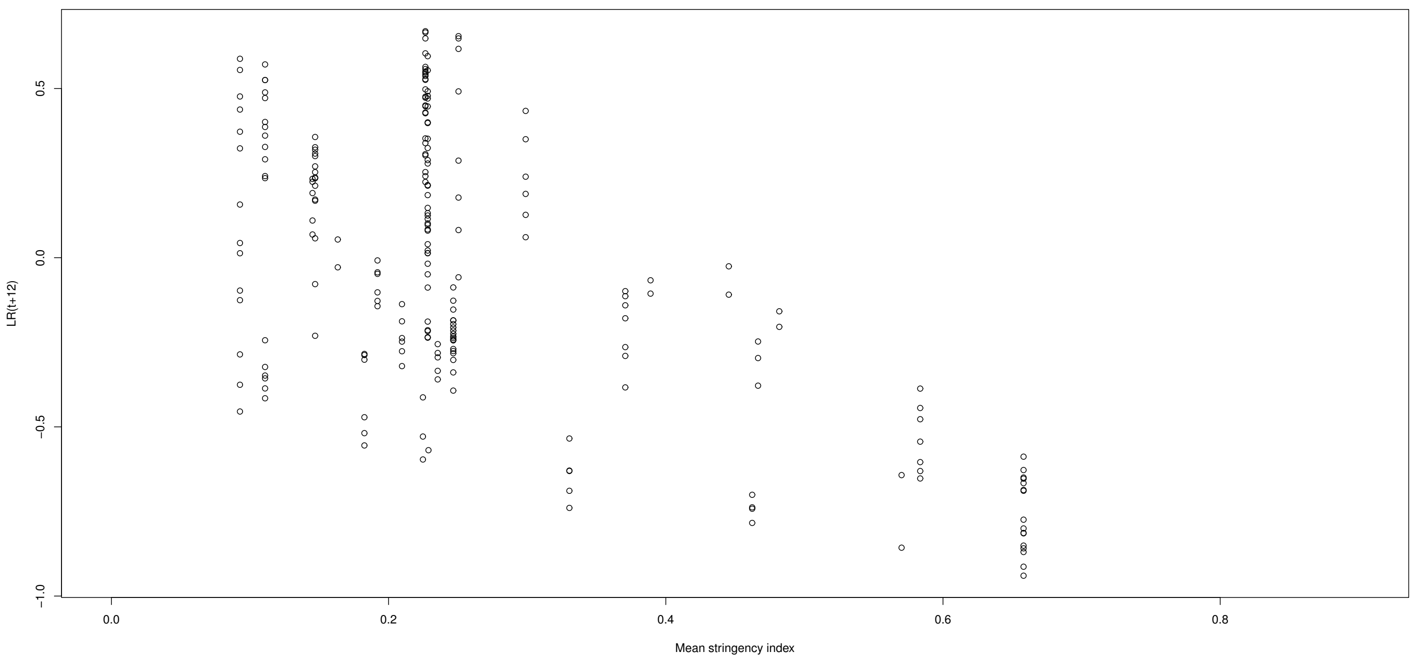

Supplementary Figure 18: Scatter plots for the mean strigency index and LR_{t+12} for Palencia (top) and Las Palmas (bottom).

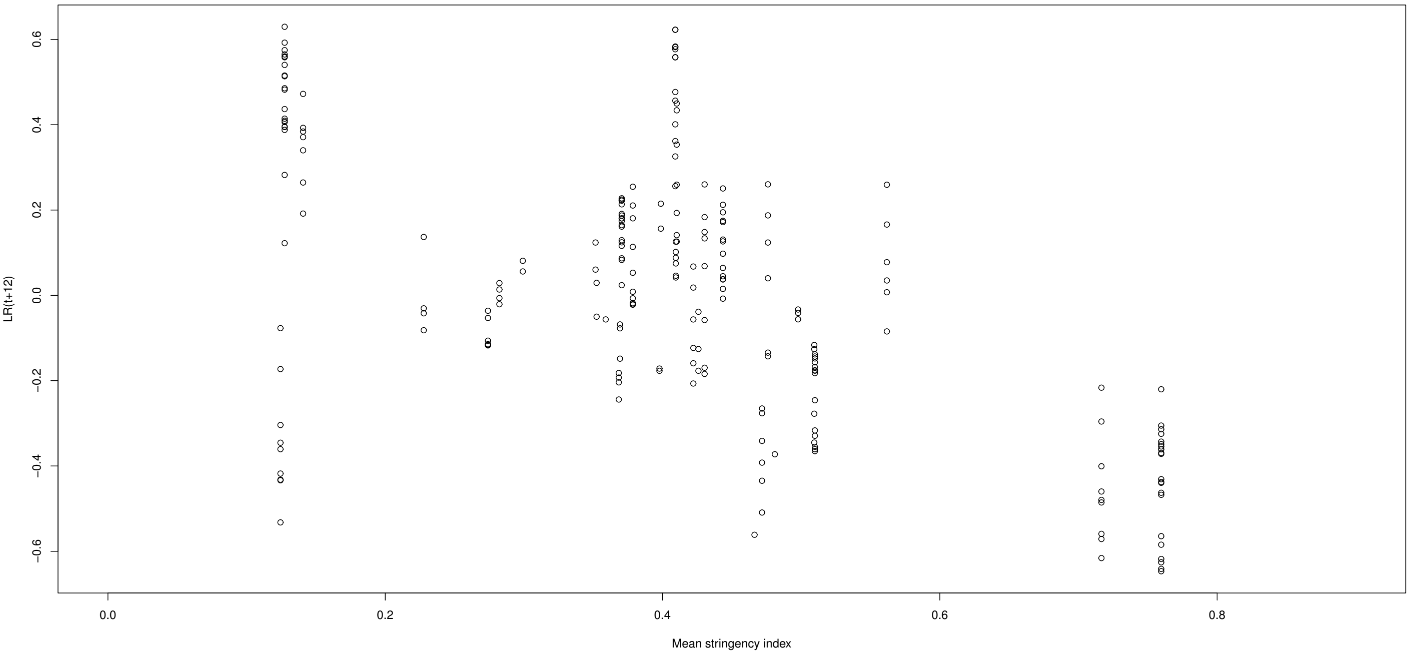

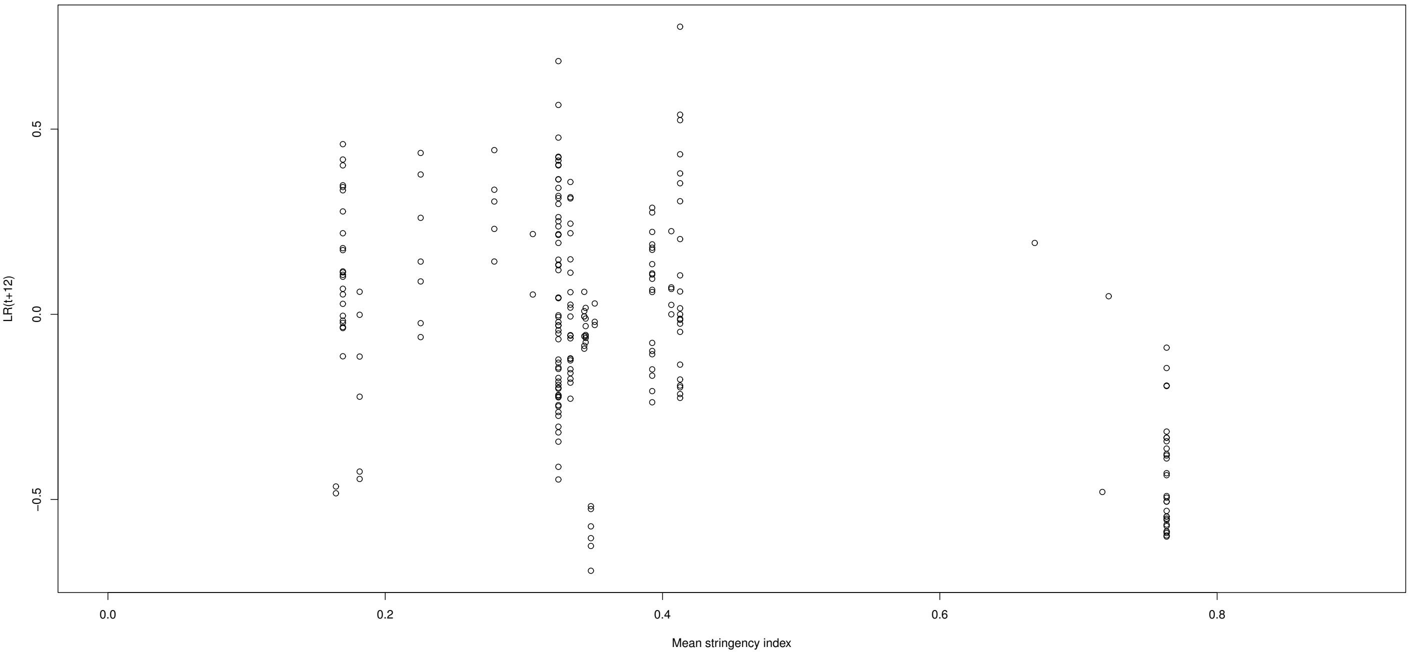

Supplementary Figure 19: Scatter plots for the mean strigency index and LR_{t+12} for Pontevedra (top) and La Rioja (bottom).

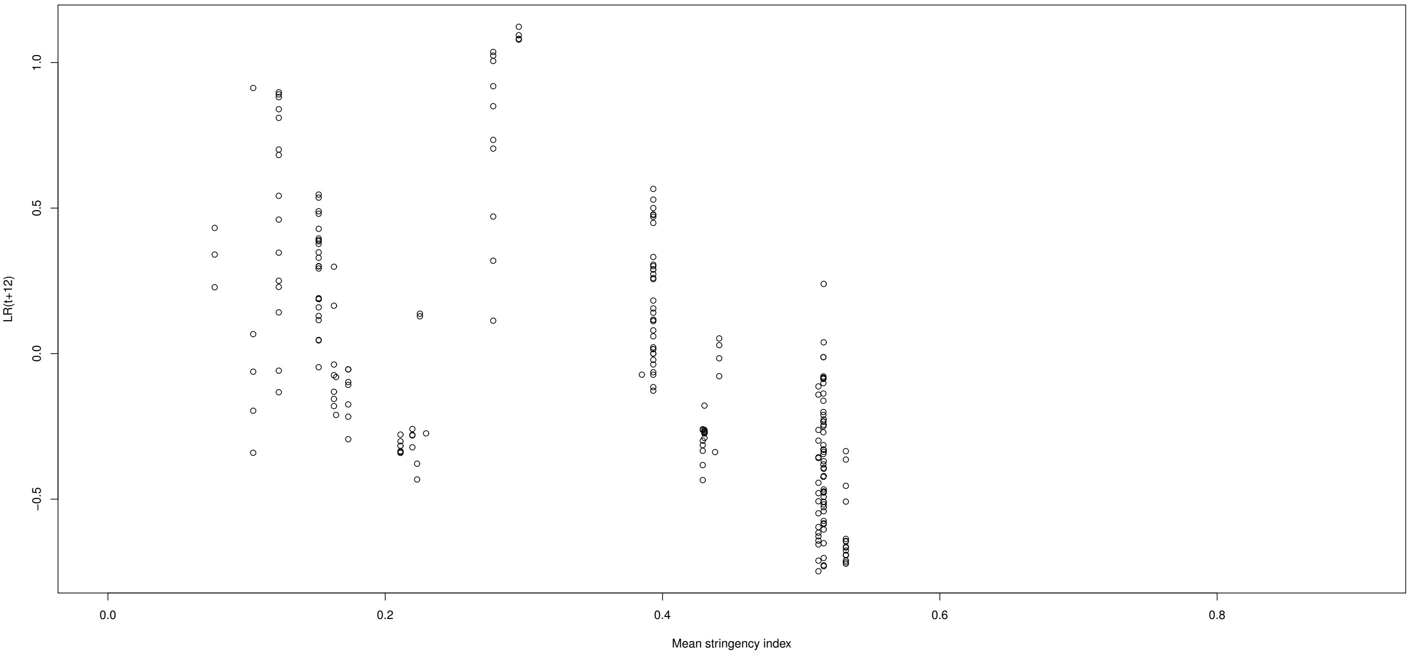

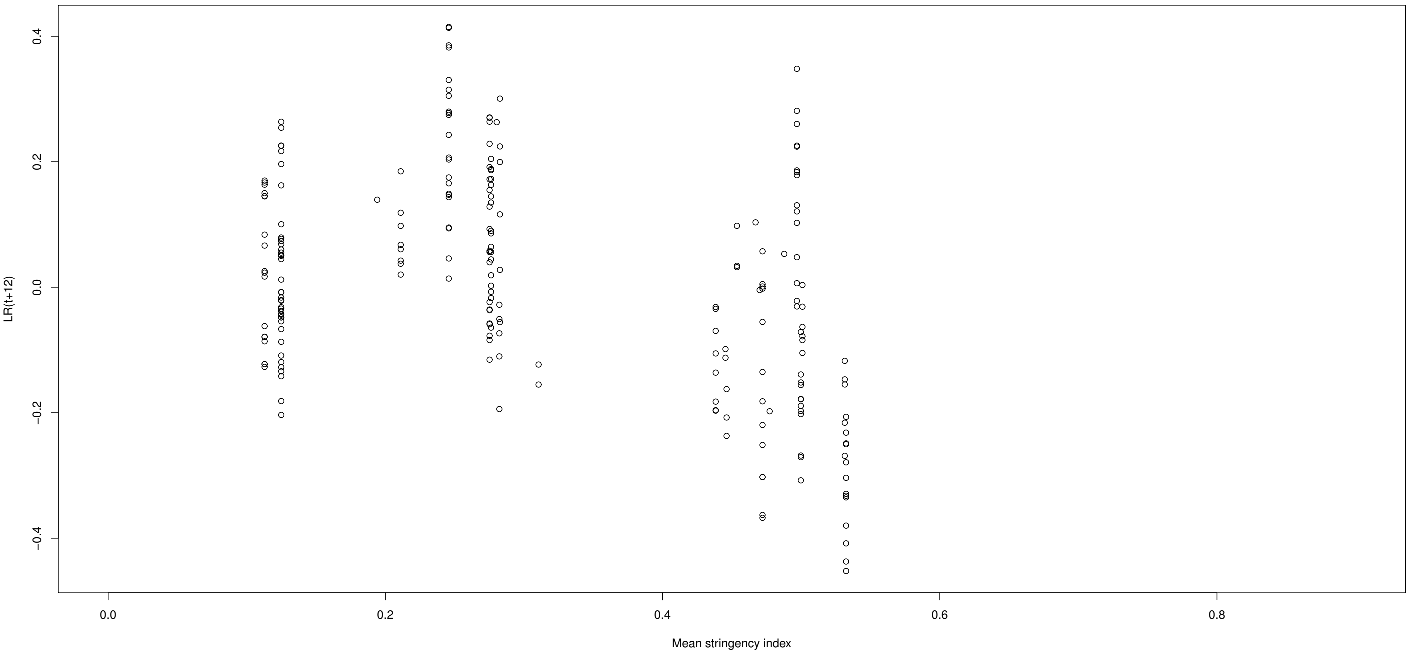

Supplementary Figure 20: Scatter plots for the mean strigency index and LR_{t+12} for Salamanca (top) and Santa Cruz de Tenerife (bottom).

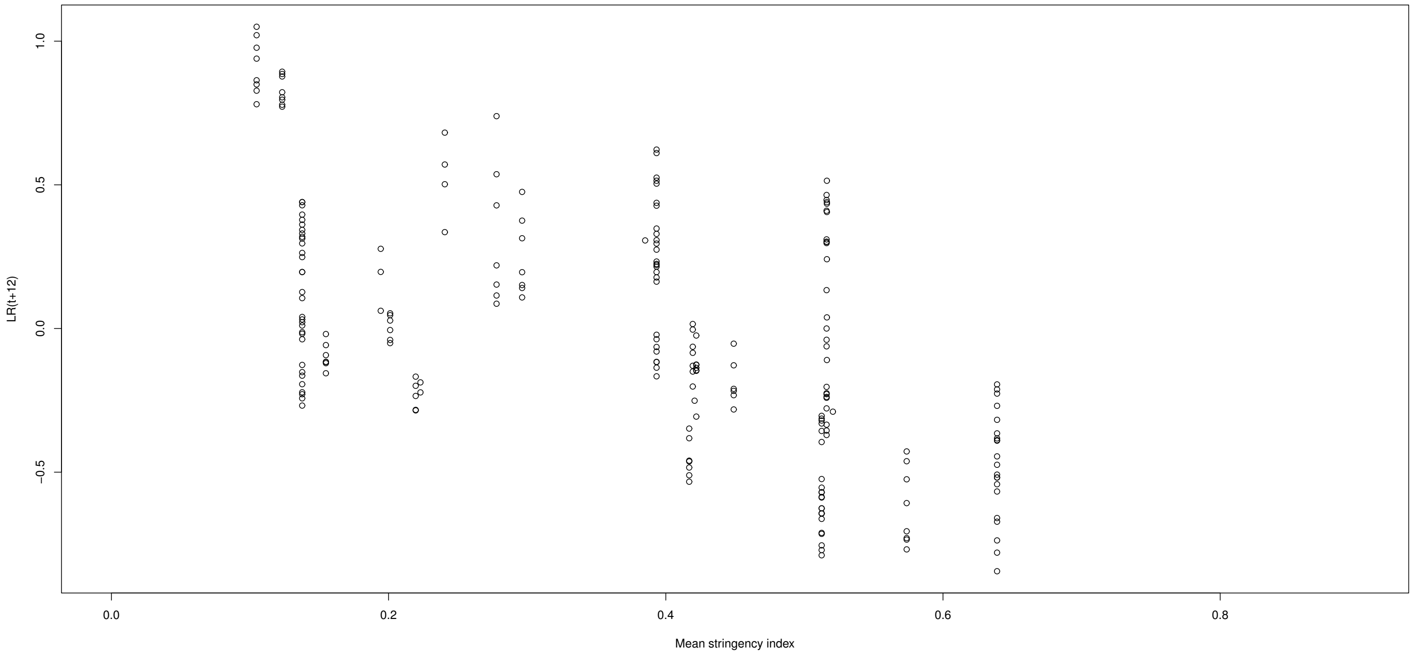

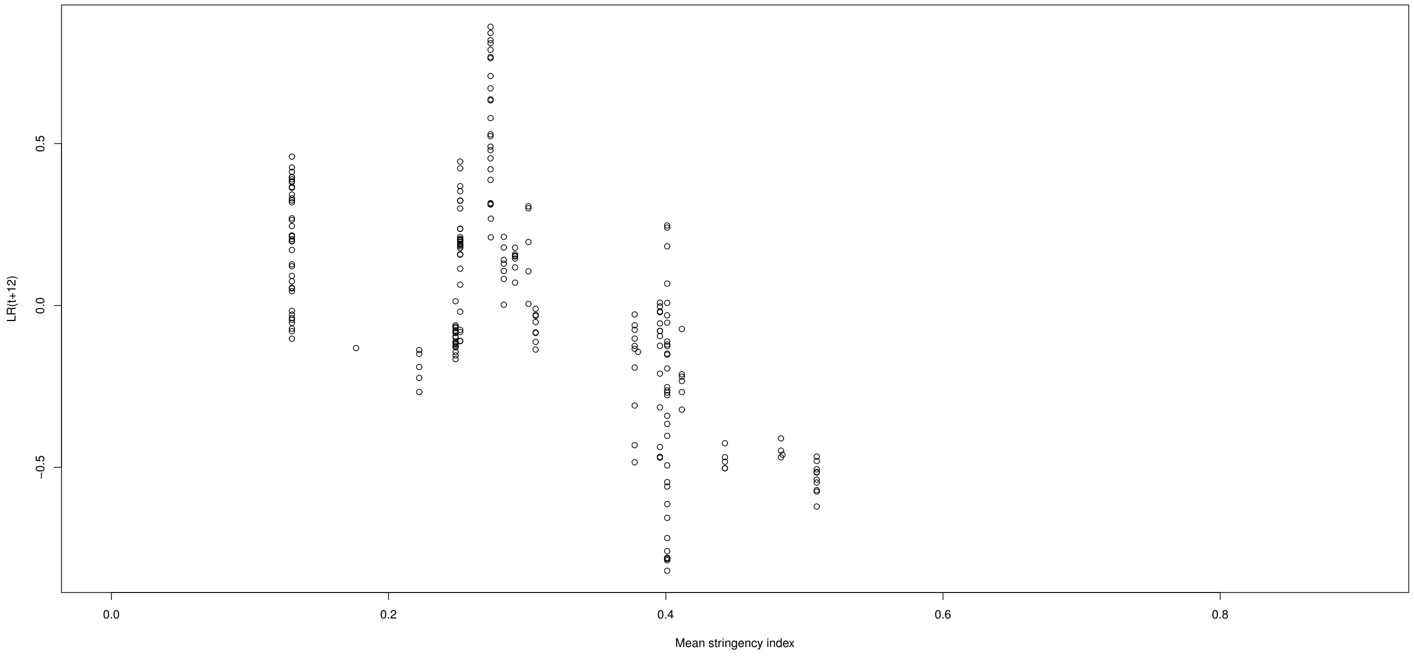

Supplementary Figure 21: Scatter plots for the mean strigency index and LR_{t+12} for Segovia (top) and Sevilla (bottom).

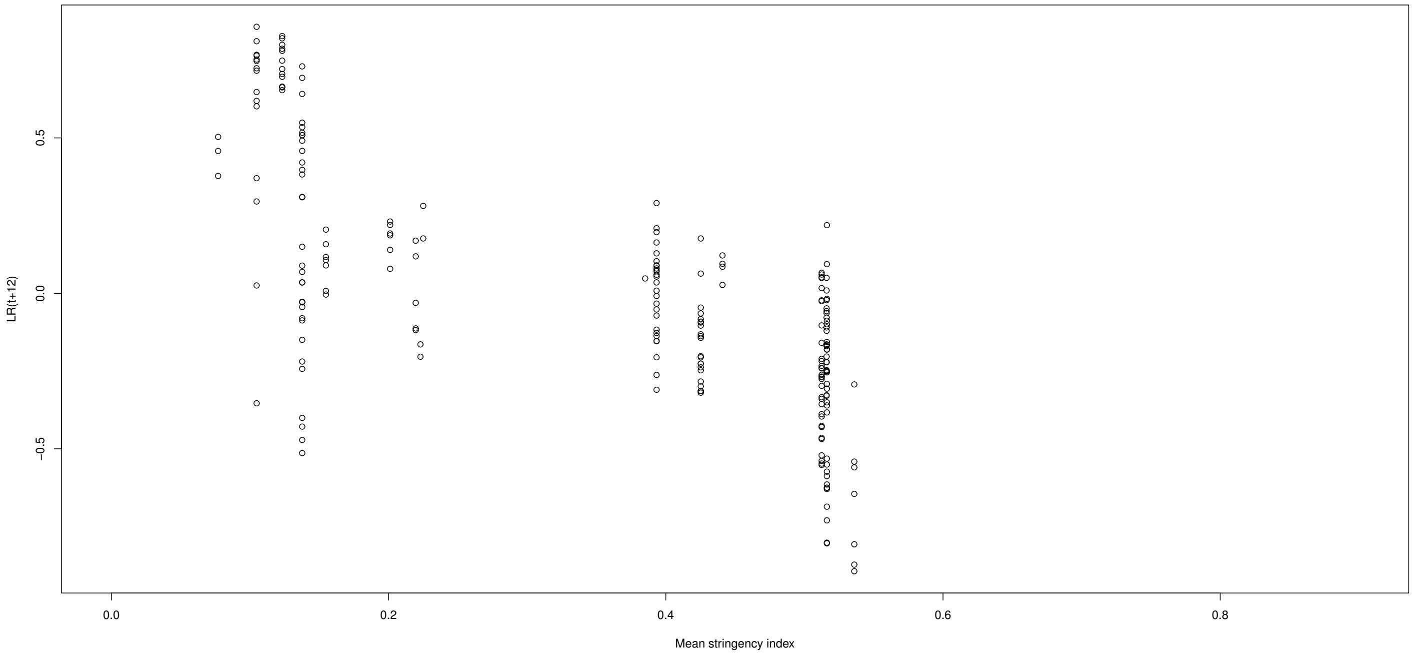

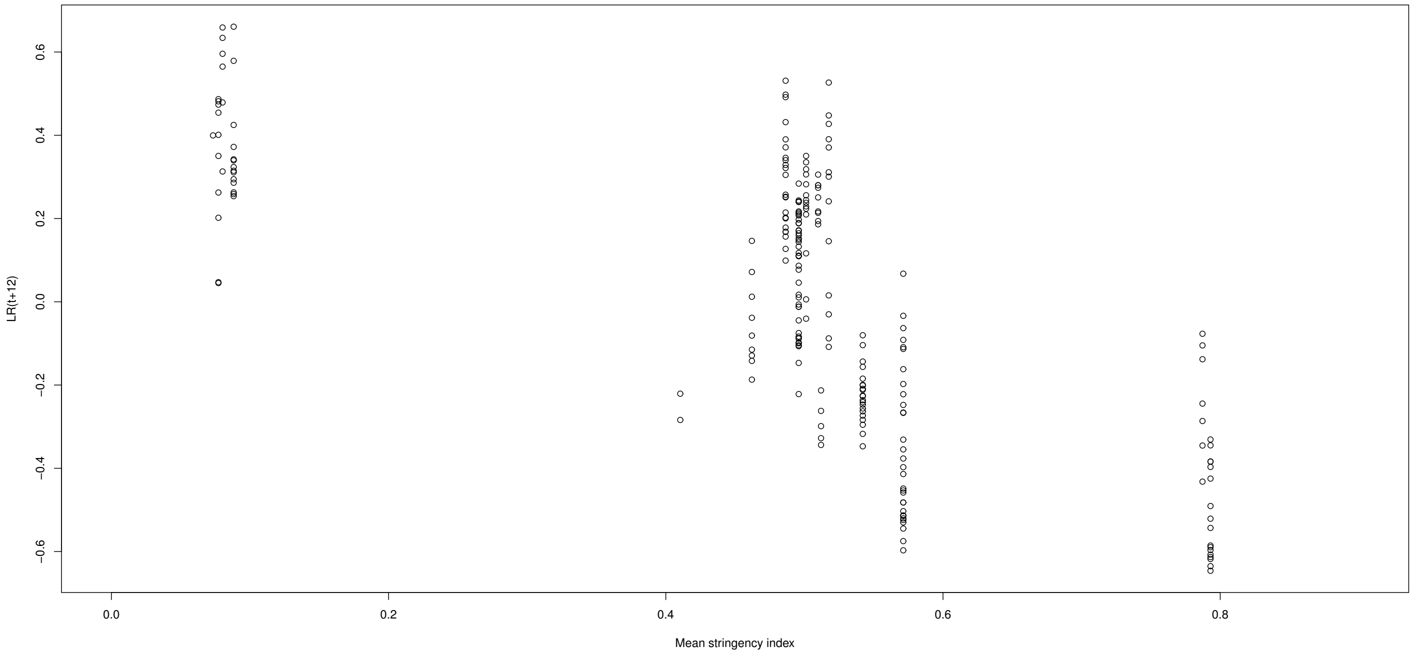

Supplementary Figure 22: Scatter plots for the mean strigency index and LR_{t+12} for Soria (top) and Tarragona (bottom).

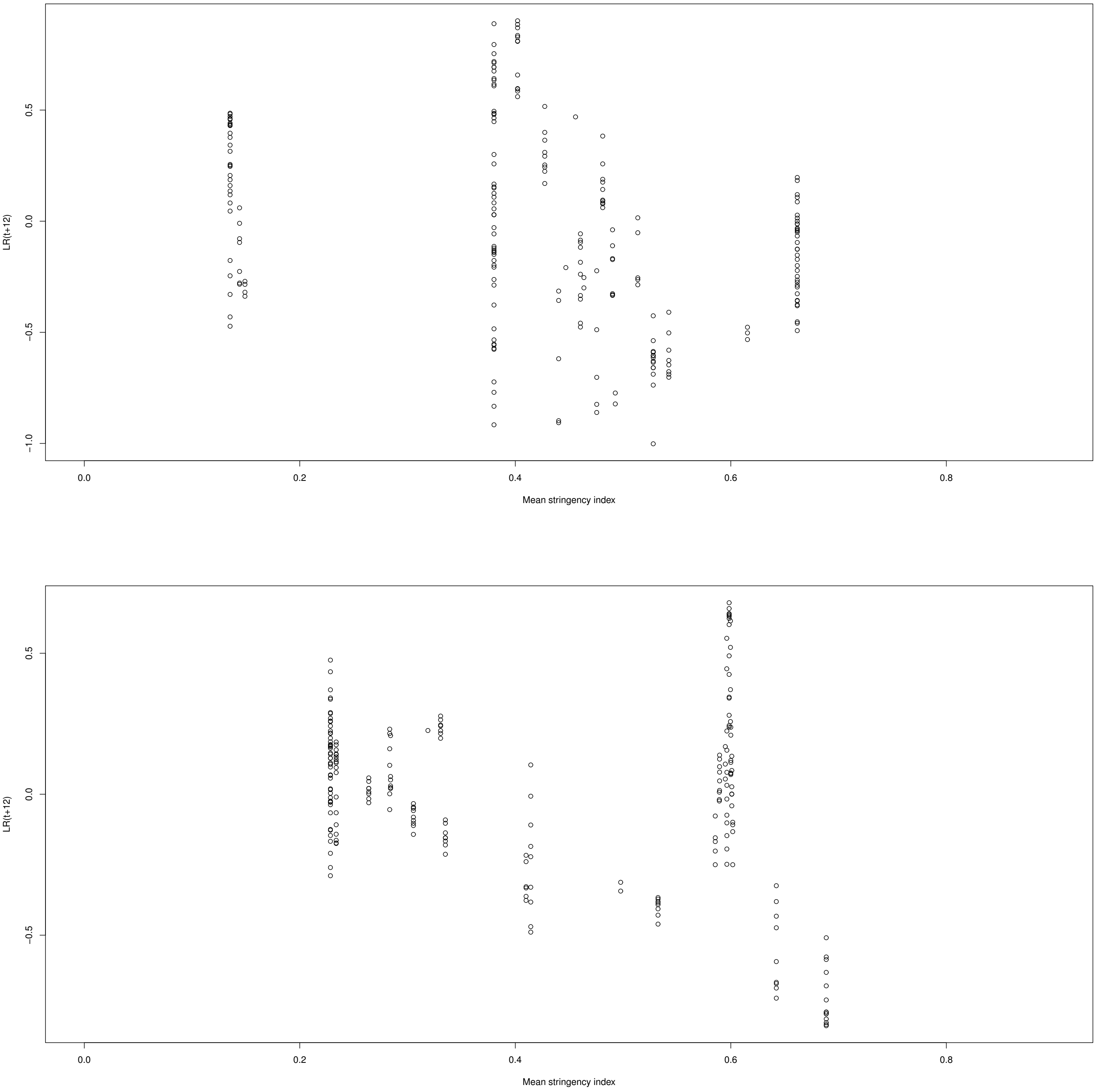

Supplementary Figure 23: Scatter plots for the mean strigency index and LR_{t+12} for Teruel (top) and Toledo (bottom).

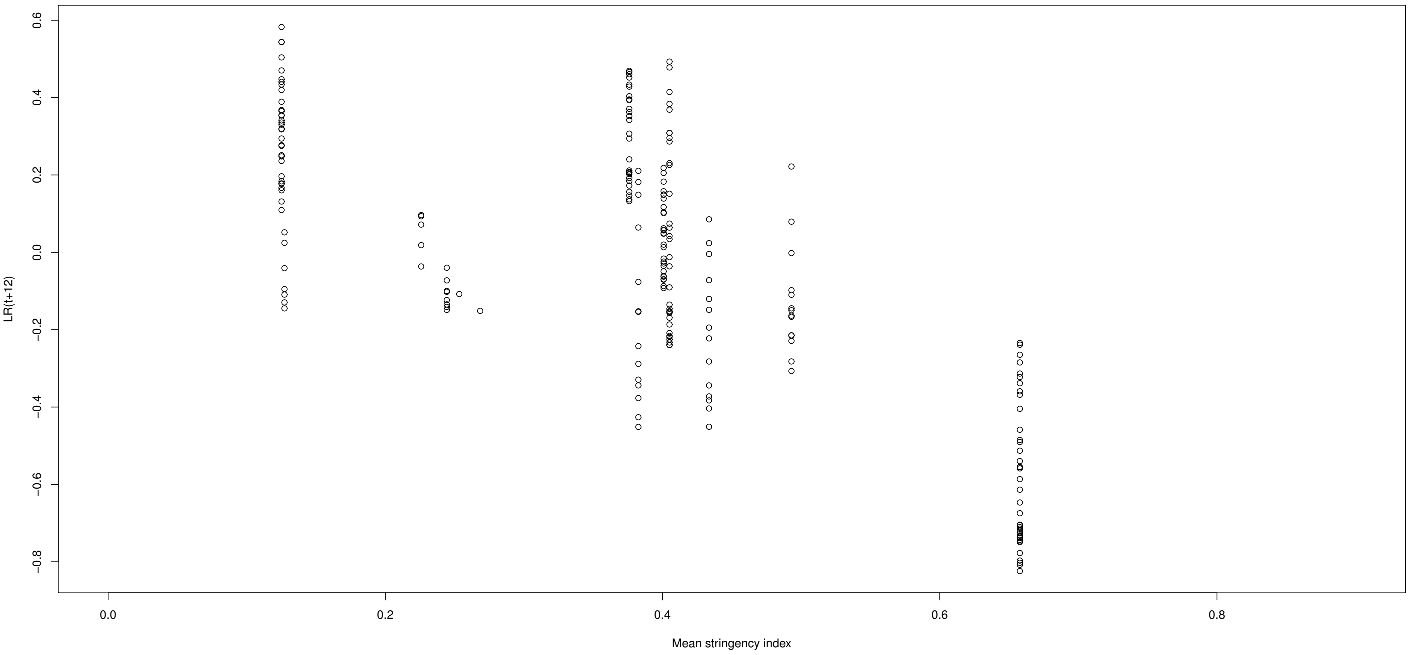

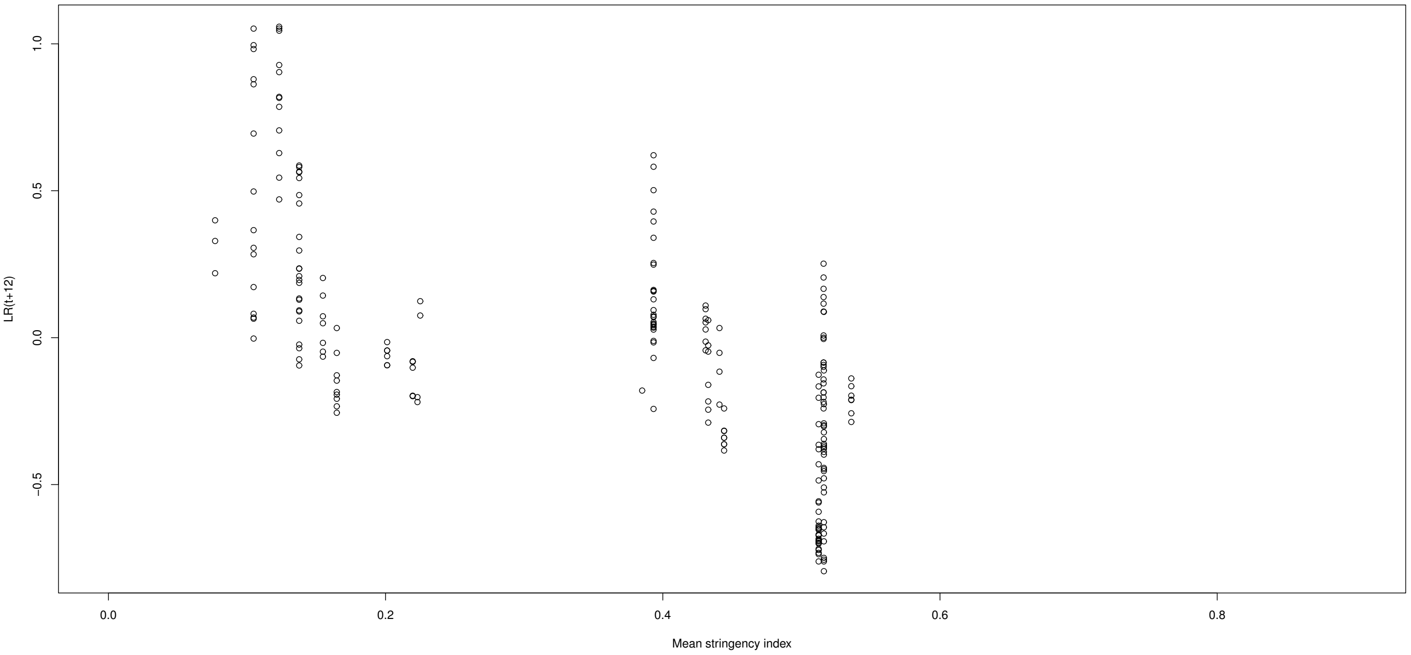

Supplementary Figure 24: Scatter plots for the mean strigency index and LR_{t+12} for Valencia (top) and Valladolid (bottom).

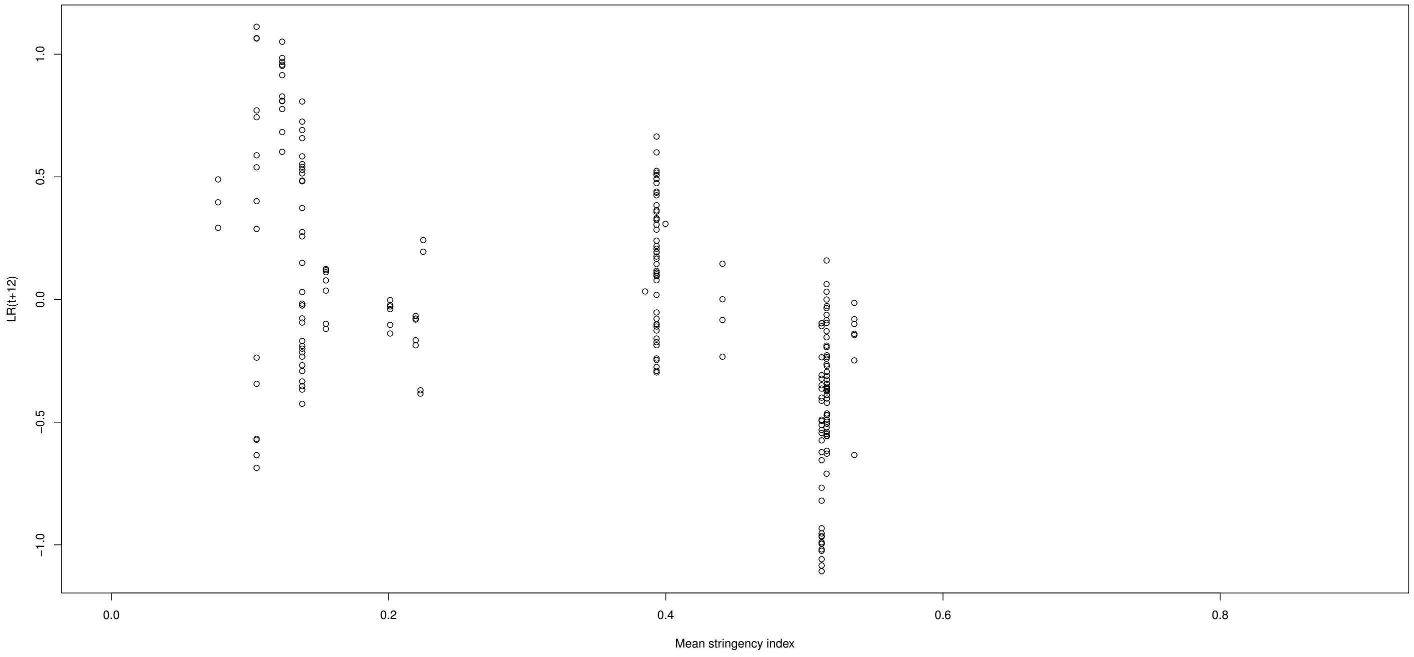

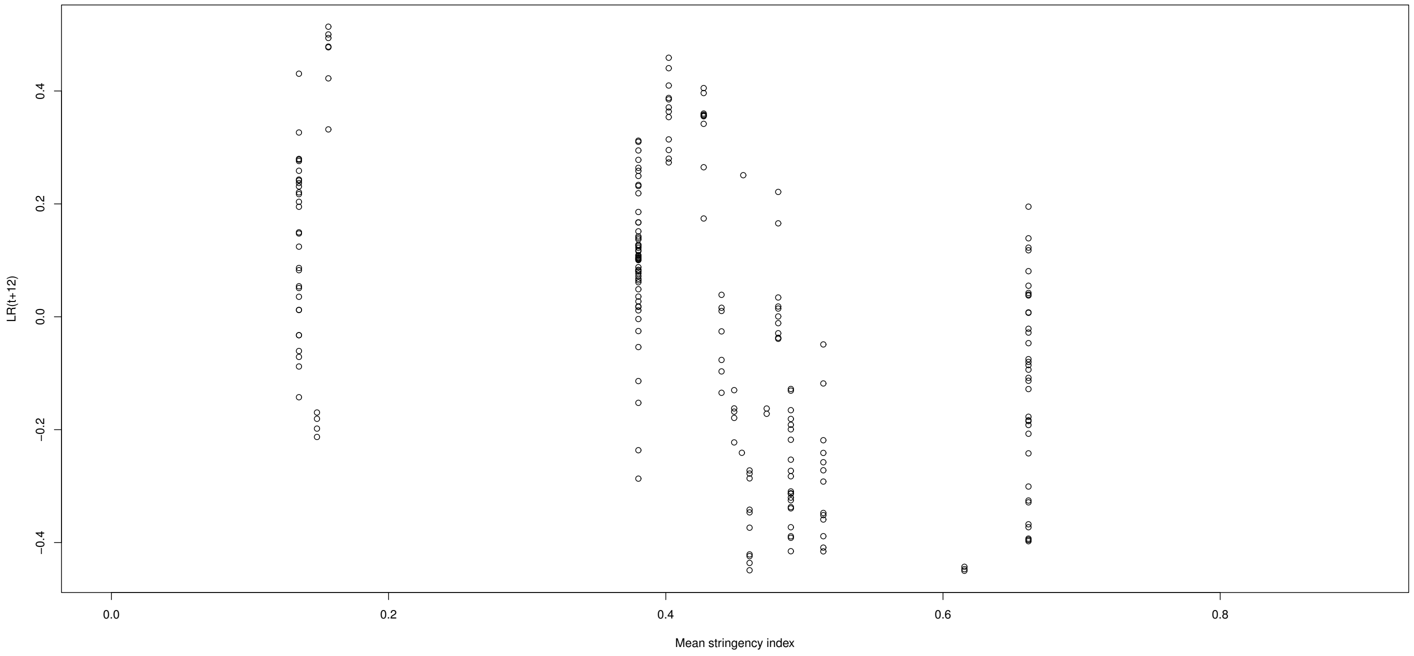

Supplementary Figure 25: Scatter plots for the mean strigency index and LR_{t+12} for Zamora (top) and Zaragoza (bottom).

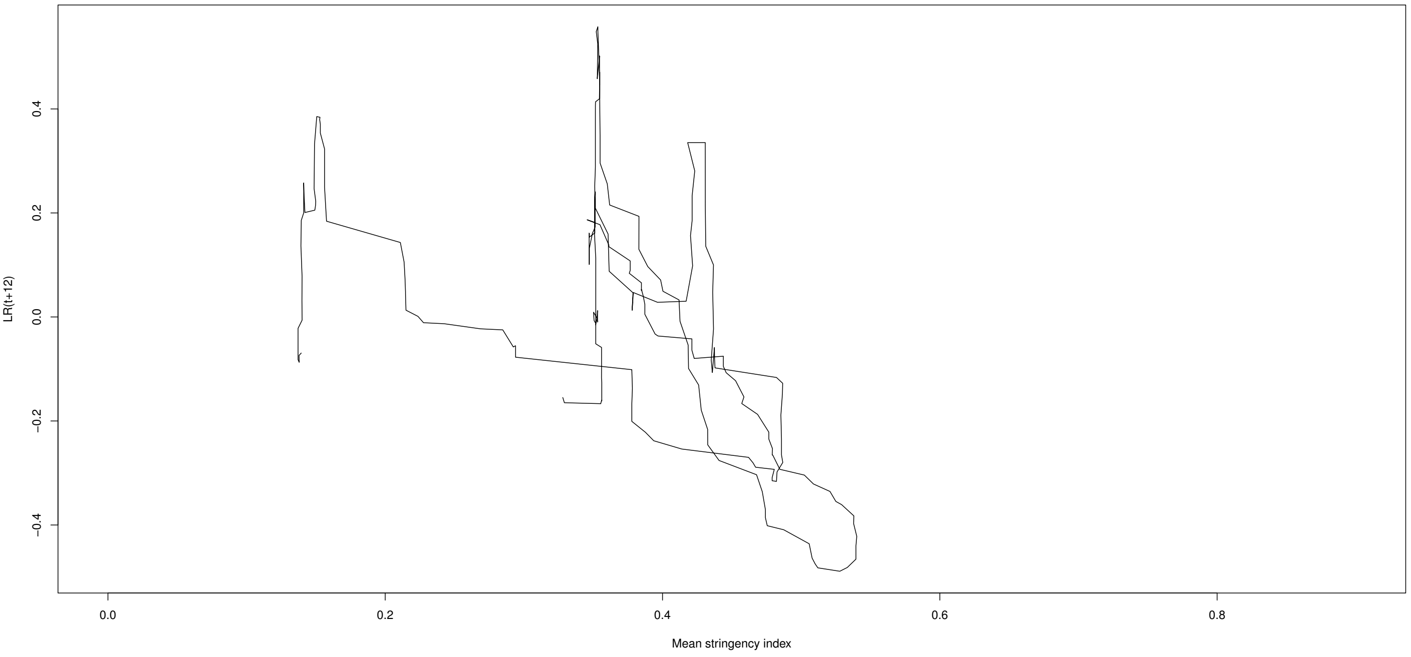

Supplementary Figure 26: Scatter plot for the mean strigency index and LR_{t+12} for Spain. Consecutive data are joined with segments to show evolution in time

Appendix VIII. Estimated coefficients for the hierarchical multiplicative model (HMM) considered.

	INTER	OUTSP	INSP	CULT	CERE	COMM	INRE	OUTRE	DIST	MOBI
A Coruña	0.97	1.09	0.91	0.88	1.02	1.21	0.82	1	0.85	1.19
Lugo	1.15	0.85	1.39	0.91	1.11	1.19	0.66	0.84	0.99	1.11
Pontevedra	1.02	2.20	0.51	1.01	1.26	0.84	0.81	1.02	0.90	0.90
Ourense	0.71	3.82	0.44	0.79	1.13	1.13	0.56	1.32	0.64	1.21
Asturias	0.37	0.91	1.26	0.95	0.46	1.02	0.63	1.17	1.07	1.34
Cantabria	0.83	1.25	0.94	0.74	0.68	1.02	1.27		0.88	0.95
Bizkaia	7.10	0.18			12.18	0.59	1.57	1.12	0.90	1.03
Guipuzkoa	1.55	0.64			2.59	0.88	0.59	1.38	0.88	1.07
Álava	0.73	1.04			1.34	0.81	0.64	1.19	0.91	1.08
Navarra	0.85	1.13	0.74	1.19	0.97	0.76	0.87	0.97	1.45	0.95
La Rioja	1.51	1.13	0.77	0.99	1.38	0.84	0.92	0.97	1.20	0.83
Huesca	0.66	0.28	4.53	1.48	1.02	2.64	0.57	0.73	0.37	1.06
Zaragoza	1.57	0.64	2.20	0.24	0.97	0.64	1.34	0.76	1.82	1.11
Teruel	0.55	4.06	0.33	3.29	0.73	2.18	0.65	1.51	0.14	1.43
León	0.63	4.26	1.08	0.04	0.73	1.27	0.74	1.03	0.71	1.09
Palencia	0.96	1.52	0.91	0.72	0.75	1.15	0.93	0.94	0.79	1.11
Burgos	0.97	1.49	1.32	0.77	0.82	0.79	0.80	0.78	0.79	1.09
Soria	0.91	1.60	1.12	0.53	0.73	0.95	0.90	0.77	0.87	1.12
Zamora	0.89	2.46	1.02	0.47	0.70	0.84	0.87	0.73	0.77	1.16
Valladolid	0.91	1.68	0.93	0.65	0.71	1.08	0.87	0.84	0.76	1.26
Salamanca	0.73	2.83	1.32	0.42	0.52	1.21	0.64	0.87	0.43	1.52
Ávila	0.44	4.48	11.59	0	0.75	0.72	0.15	1.20	0.83	1.08
Segovia	0.98	1.26	0.81	0.84	0.80	0.92	1.11	0.92	0.79	1.26
Madrid	0	0	1.08×10^{27}		9.81×10^{18}	0	0.52	0.60	0.94	0.97
Guadalajara	1.14	1.36	0.66	1	0.67	2.48	1.39	0.51	1.07	0.91
Cuenca	1.84	1.11	0.75	0.84	0.94	2.10	3.49	0.30	0.98	0.83
Toledo	0.91	1.22	0.92	1.07	0.49	4.26	0.58	0.74	1.08	0.84
Ciudad Real	0.97	1.08	0.77	1	1.32	1.08	1.12	0.69	0.86	1.02
Albacete	0.49	0.83	1.15	1.25	2.23	0.42	0.50	1.63	0.66	1.04
Castellón	0.58	0.26	1.55	0.91	2.66	0.44	1.08	2.03	1.40	0.51
Valencia	0.56	0.64	1.05	0.63	1.67	0.54	1.11	1.27	1.01	0.80
Alicante	1.14	0.66	1.13	1.99	1.58	0.56	0.87	1.28	1.21	0.68
Murcia	0.08	0.83	0.89	0	1.23	3.60	1.09	0.90	0.64	0.76
Huelva	5.19×10^{5}	0	1.46×10^{31}	0	0.74	0.59	1261.43	0	0.08	0.75
Sevilla	1.04	757.48	0	46.99	0.19	0.84	41.68	0.03	0.48	0.97
Cádiz	0	1.42×10^{19}	0	1.9×10^{11}	0.63	0.50	17.46	0.04	0.50	0.84
Córdoba	2.80	0.23	30.57	1.25	0.58	0.52	10.70	0.04	0.50	1.01
Málaga	2.33×10^{5}	0	4.47×10^{28}	0	0.27	1.55	281.46	0	0.18	0.91
Jaén	3.13	0.17	52.46	0.54	0.25	0.79	17.99	0.06	0.90	0.78
Granada	0.01	1.7×10^{15}	0	1.2×10^{8}	1.03	3.22	230.44	0.01	0.06	0.97
Almería	0	1.83×10^{25}	0	5.79×10^{13}	0.53	1.34	57.40	0.05	0.18	1
Cáceres	1.09	1.88	0.70	0.66	0.90	1.16	0.98	0.81	0.94	1.06
Badajoz	0.97	1.28	1.08	0.68	0.98	1.03	0.66	1.38	1.07	0.87
Illes Balears	0.51	0.18	1.20	0.45	1.93	0.33	0.73	3.86	0.56	1.54
Las Palmas	1.79	0.27	2.51	0.56	1.19	0.74	1.06	2.83	1.05	1.39
Santa Cruz	2.64	0.38	3.10	8.85	0.34	1.42	0.96	1.05	0.86	0.98
Barcelona	1.27	1.09	0.86	0.95	1.22	0.91	0.42	1.54	1.62	0.92
Girona	1.45	0.85	0.92	1.14	1.36	0.96	0.90	0.90	1.30	0.77
Lleida	0.90	1.16	0.87	0.80	1.13	0.93	0.40	1.97	1.08	1.14
Tarragona	1.52	1.13	0.77	0.99	1.38	0.83	0.92	0.97	1.21	0.83

Supplementary Table 62: Estimation of $\hat{\alpha}_i, \forall i \in \{0, \dots 9\}$ in the HMM considered $e^{LR_{t+12}} = \hat{\alpha}_0 \cdot \hat{\alpha}_1^{OUTSP} \cdot \hat{\alpha}_2^{INSP} \cdot \hat{\alpha}_3^{CULT} \cdot \hat{\alpha}_4^{CERE} \cdot \hat{\alpha}_5^{COMM} \cdot \hat{\alpha}_6^{INRE} \cdot \hat{\alpha}_7^{OUTRE} \cdot \hat{\alpha}_8^{DIST} \cdot \hat{\alpha}_9^{MOBI}$, with standarized explanatory variables.

Appendix IX. Percentage of significant coefficients (along provinces) for every stringency index using classical methods as well as methods for controlling the family-wise error rate (FWER) and the false discovery rate (FDR).

METHOD		FDR			FWER			CLASSIC		
α		0.01	0.05	0.1	0.01	0.05	0.1	0.01	0.05	0.1
**INTERCEPT**	$\hat{\beta}_0$	28.57	46.94	61.22	24.49	40.82	48.98	34.69	55.10	65.31
**OUTSP**	$\hat{\beta}_1$	53.06	65.31	71.43	46.94	59.18	65.31	55.10	69.39	79.59
**INSP**	$\hat{\beta}_2$	32.65	46.94	51.02	28.57	34.69	48.98	34.69	51.02	57.14
**CULT**	$\hat{\beta}_3$	36.73	44.90	55.10	32.65	36.73	44.90	38.78	48.98	55.10
**CERE**	$\hat{\beta}_4$	63.27	69.39	75.51	55.10	63.27	67.35	63.27	77.55	77.55
**COMM**	$\hat{\beta}_5$	38.78	51.02	59.18	34.69	42.86	53.06	40.82	57.14	61.22
**INRE**	$\hat{\beta}_6$	40.82	59.18	61.22	36.73	48.98	59.18	46.94	61.22	69.39
**OUTRE**	$\hat{\beta}_7$	51.02	57.14	63.27	46.94	55.10	59.18	55.10	65.31	75.51
**DIST**	$\hat{\beta}_8$	46.94	61.22	71.43	42.86	55.10	61.22	53.06	69.39	79.59
**MOBI**	$\hat{\beta}_9$	34.69	48.98	61.22	30.61	44.90	46.94	42.86	55.10	67.35

Supplementary Table 63: Percentage (%) of times $H_0 : \hat{\beta}_i = 0, \forall i \in \{0, \ldots 9\}$ is rejected, taking into account FWER, FDR and classic method, as well as the MLR given by $LR_{t+12} = \hat{\beta}_0 + \hat{\beta}_1 OUTSP + \hat{\beta}_2 INSP + \hat{\beta}_3 CULT + \hat{\beta}_4 CERE + \hat{\beta}_5 COMM + \hat{\beta}_6 INRE + \hat{\beta}_7 OUTRE + \hat{\beta}_8 DIST + \hat{\beta}_9 MOBI$. Three values for α are considered: $\alpha \in \{0.01,0.05,0.1\}$.
